# 2D Materials Powering Neuromorphic Intelligence

**DOI:** 10.1007/s40820-026-02253-1

**Published:** 2026-06-24

**Authors:** Jamal Kazmi, Waqas Ahmad, Muhammad Naqi, Yawar Abbas, Aumber Abbas, Peijian Wang, Mohd Ambri Mohamed, Federico Rosei, Zhiming M. Wang, Hongwei Song

**Affiliations:** 1 Department of Physics, College of Sciences, Shanghai University, Shanghai, 200444 People’s Republic of China; 2 Laboratory of 2D Optoelectronics and Nanoelectronics (L2DON), State Key Laboratory of Quantum and Functional Materials, Department of Materials Science and Engineering, Southern University of Science and Technology, 1088 Xueyuan Vlvd, Shenzhen, 518000 People’s Republic of China; 3 School of Engineering, Liverpool John Moores University, Liverpool, L3 3AF UK; 4 Electronic and Nanoscale Engineering Division, James Watt School of Engineering, University of Glasgow, Glasgow, G12 8QQ Scotland, UK; 5 Department of Mechanical Engineering and Interdisciplinary Research Center for Hydrogen Technologies & Carbon Management, King Fahd University of Petroleum and Minerals (KFUPM), 31261 Dhahran, Saudi Arabia; 6 Key Laboratory of Carbon Materials of Zhejiang Province, College of Chemistry and Materials Engineering, Wenzhou University, Wenzhou, 325035 People’s Republic of China; 7 Materials Science and Engineering, National University of Singapore, Singapore, 138602 Singapore; 8 Institute of Microengineering and Nanoelectronics (IMEN), Universiti Kebangsaan Malaysia (UKM), 43600 Bangi, Selangor Malaysia; 9 Department of Chemical and Pharmaceutical Sciences, University of Trieste, Via Gorgerin 1, 34127 Trieste, Italy; 10 The School of Physics, University of Electronic Science and Technology of China, Chengdu, 611731 People’s Republic of China; 11 Shimmer Center, Tianfu Jiangxi Laboratory, Chengdu, 641419 People’s Republic of China

**Keywords:** 2D neuromorphic computing, Machine-learning integration, Artificial synaptic devices, Transition metal dichalcogenides, Quantum-inspired neuromorphics

## Abstract

The exponential demand for energy-efficient and adaptive computing architectures drives the evolution of artificial intelligence (AI) and machine learning (ML). Neuromorphic computing, inspired by biological neural networks, overcomes the limitations of traditional von Neumann architectures, including high energy consumption and limited scalability. The introduction of two-dimensional (2D) materials, such as transition metal dichalcogenides, hexagonal boron nitride, black phosphorus, and tellurene, enables neuromorphic devices with unprecedented control over electronic and optoelectronic properties. These materials exhibit atomic-scale thickness, high carrier mobility, and tunable bandgaps, facilitating synaptic behaviours such as spike-timing-dependent plasticity and paired-pulse facilitation. This review describes the integration of 2D materials into neuromorphic systems, highlighting applications in wearable electronics, brain–machine interfaces, and quantum neuromorphic platforms. In wearable and edge computing, 2D-based devices enable localized, ultra-low-power data processing. In brain–machine interfaces, they enhance signal transduction and neural interfacing. Quantum effects in 2D materials further enable hybrid quantum–classical neuromorphic architectures for high-dimensional computational tasks. Despite significant advances, challenges in reproducibility, scalability, and stability remain. Addressing these limitations through innovations in synthesis and defect passivation is essential for practical application. This review underscores the transformative potential of 2D-material-based neuromorphic computing for energy-efficient AI.

## Highlights

- Two-dimensional materials enable energy-efficient neuromorphic computing through gate tuneable bandgaps, fast switching kinetics, and compatibility with spiking neural networks for adaptive learning.
- Emerging memory devices leveraging 2D materials—including resistive, ferroelectric, and phase-change systems—mimic synaptic plasticity to revolutionize neuromorphic architectures.
- Future advances in thin-film synthesis, defect engineering, and quantum-inspired designs will unlock scalable, sustainable 2D neuromorphic systems for healthcare, edge AI, and quantum computing.

## Graphical abstract

**Figure:**
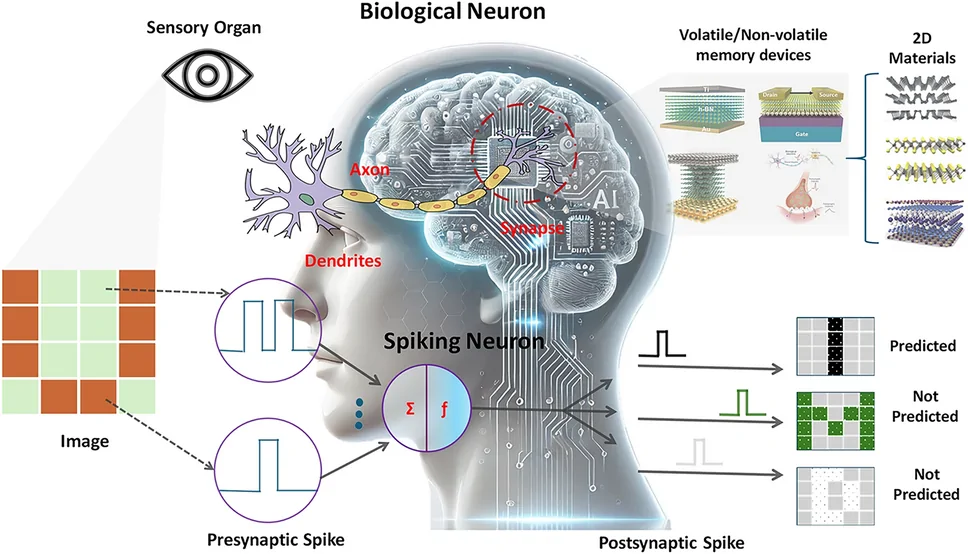
Integration of 2D materials into neuromorphic computing architectures offers a promising pathway toward energy-efficient and adaptive systems that bridge biological learning mechanisms with machine intelligence

## Introduction

### Background

The rise of Artificial Intelligence (AI) is prompting an increasing demand for neuromorphic computing, particularly in-memory technologies, driven by revolutions in algorithms and architectures aimed at replicating the processing efficiency of biological systems [1–3]. Neuromorphic computing integrates analogue computation, data storage, and biological emulation, enabling hardware that operates in a brain-like manner [4–19]. Memristors are key building blocks of neuromorphic platforms because they enable synaptic-like behaviour and energy-efficient memory storage. In contrast, conventional von Neumann architectures separate memory and processing units, creating substantial latency and energy costs for large-scale, data-intensive tasks such as Internet of Things (IoT) applications [20–22].

The term “neuromorphic”, first introduced by Carver Mead in the 1980s [23], refers to electronic systems designed to emulate the structure and function of biological neural networks. Early studies focused on analogue electronic circuits that replicated neuronal behaviour [24], while modern neuromorphic platforms have evolved into large-scale systems capable of brain-inspired sensing, memory, and information processing [25]. This overview provides insights into how neuromorphic systems bypass the von Neumann bottleneck and the energy-intensive demands of digitization.

**Fig. 1:**
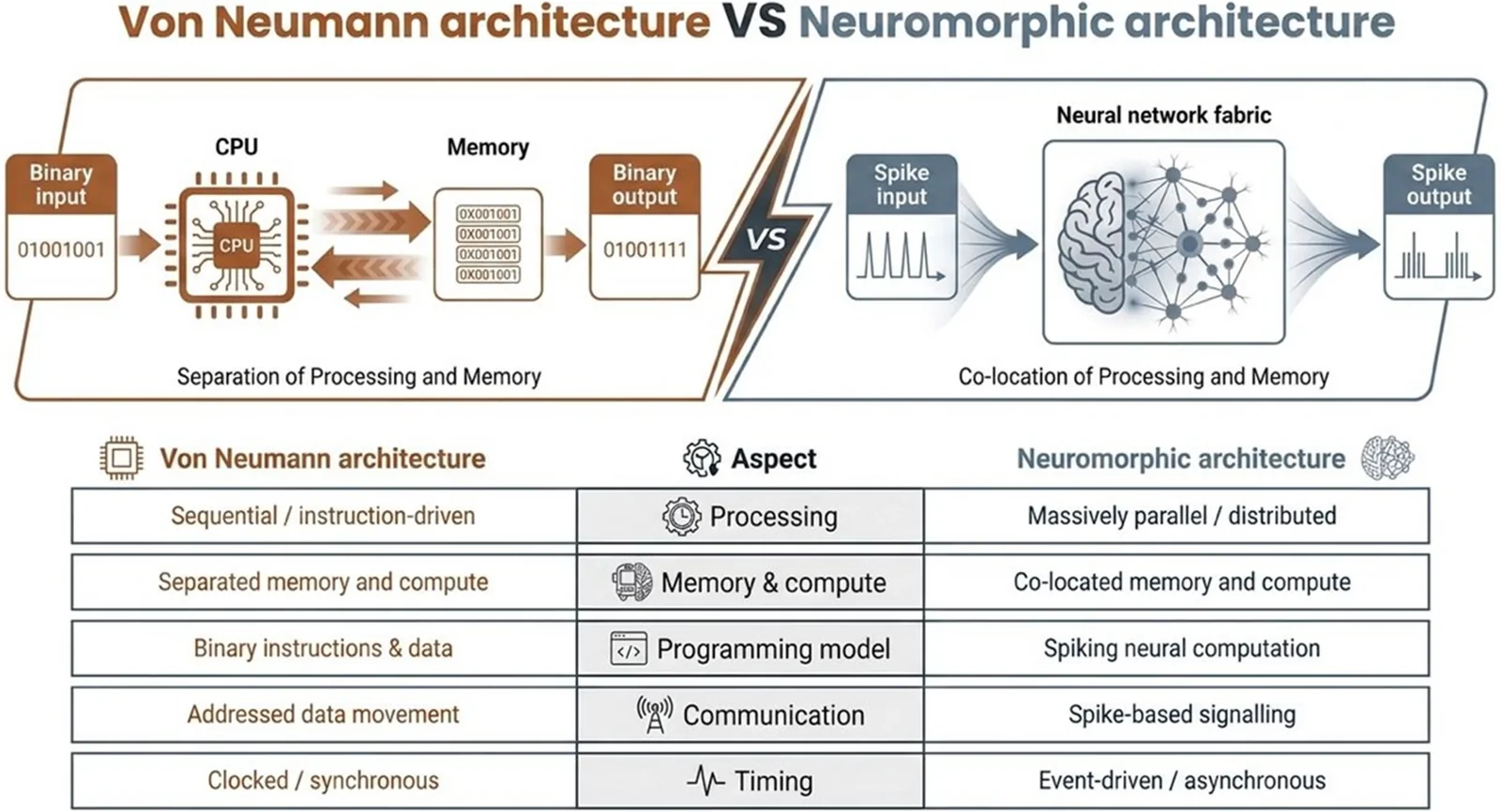
Comparison of the von Neumann architecture with the neuromorphic architecture

Figure 1 illustrates the fundamental shift from the traditional von Neumann architecture to neuromorphic computing, emphasizing the key distinctions in processing and memory integration. In contrast, neuromorphic architectures emulate the massively parallel and event-driven nature of biological neural networks, where processing and memory are co-located within artificial synapses and neurons [26–28]. This structural advantage enhances energy efficiency while simultaneously enabling real-time adaptation and learning, making neuromorphic systems highly suitable for next-generation artificial intelligence applications [2, 29–31].

Biological systems highlight the extraordinary inefficiencies of conventional central processing units (CPUs) [32]; the human brain operates at roughly 20 watts, using only 1 to 100 femtojoules per synaptic operation. The human brain operates at approximately 20 W while consuming only 1–100 fJ per synaptic event, highlighting the remarkable energy efficiency of biological information processing compared with conventional CPUs [1, 33–35]. Neuromorphic research can broadly be divided into three interconnected areas: neuromorphic engineering, neuromorphic computing, and neuromorphic devices [36, 37].

Hardware implementations rely heavily on two-terminal and three-terminal memory devices that dynamically adjust their resistance based on past states to emulate synaptic plasticity (e.g. long-term potentiation and short-term plasticity) [21, 22]. Two-terminal devices—such as resistive switching memristors (RSM), phase-change memristors (PCM), and ferroelectric memristors—are generally preferred over three-terminal transistor-based architectures due to their structural simplicity, low fabrication cost, and excellent compatibility with high-density integration and crossbar array configurations [38–42]. When integrated with CMOS circuitry and neural network algorithms, these devices support tasks such as pattern recognition, image analysis, and reinforcement learning. In such devices, resistance variations mirror synaptic weight adjustments, allowing hardware neural networks to be trained via algorithms like backpropagation [43, 44].

**Fig. 2:**
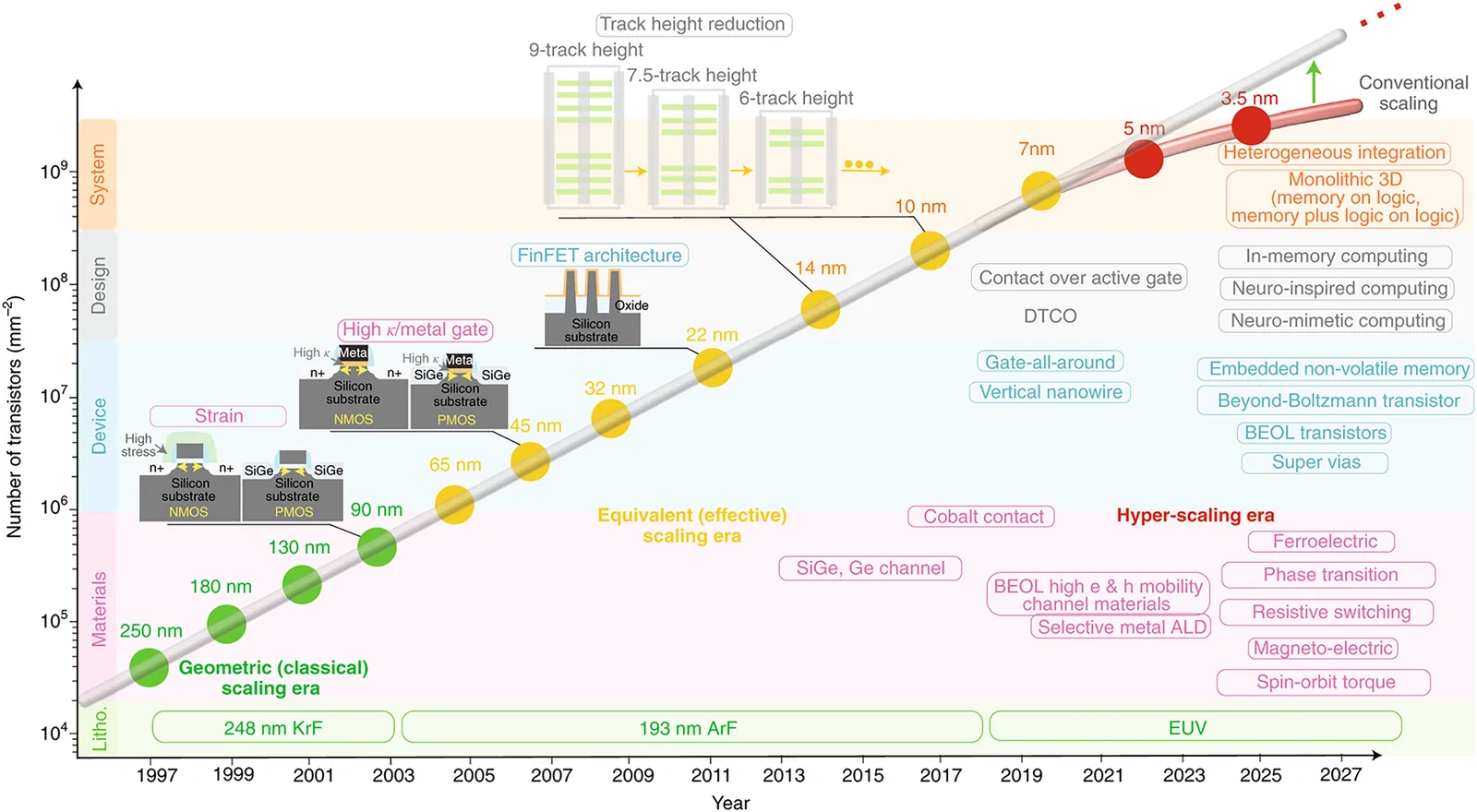
Scaling trends in electronic device density, transitioning through geometric scaling, equivalent scaling, and hyper-scaling eras. Innovations in materials, architectures, and integration enable functional diversification for next-generation electronics. Litho., lithography; BEOL, back-end-of-line; ALD, atomic layer deposition. Reproduced with permission from Ref. [56]

Three-terminal synaptic devices use the gate terminal to modulate channel conductance between the source and drain, thereby mimicking synaptic weight regulation. Examples include charge-trapping, floating gate, gate-tuneable memories, and memtransistors [45–54]. Despite these advances, variability in device architecture and non-uniform characteristics limit scalability and efficiency in large-scale applications like natural language processing and visual computing. However, variability in device structure and switching characteristics still limits scalability in large-area neuromorphic systems [55]. On the other hand, as illustrated in Fig. 2, the historical trajectory of CMOS technology scaling, from early geometric shrinking to advanced heterogeneous integration, has boosted transistor density and simultaneously guided the transition toward more sophisticated device concepts, including embedded non-volatile memory and in-memory computing platforms. This transition toward heterogeneous integration, embedded memory, and in-memory computing has created opportunities for unconventional materials and architectures, including 2D materials for neuromorphic hardware [56, 57].

Within this landscape, 2D materials, particularly transition metal dichalcogenides (TMDs), hexagonal boron nitride (h-BN), black phosphorus (BP), tellurene and other systems are emerging as promising candidates. Their atomically thin structure and unique electrical and optical properties provide tuneable bandgaps, mechanical stability and flexibility, making them ideal for both two-terminal and three-terminal devices [58–61]. The tunability of these 2D materials enables the development of optoelectronic neuromorphic systems, such as light-responsive and photo-tuneable synapses, essential for high-performance, energy-efficient computing [62]. For example, Zhang et al. [63] demonstrated a black arsenic-phosphorus phototransistor with polarization-sensitive neuromorphic behaviour, including paired-pulse facilitation and long-term plasticity, highlighting the promise of anisotropic 2D materials for optoelectronic neuromorphic systems. Given this, the scalable fabrication of high-quality, large-area 2D materials is essential for achieving uniform and reliable neuromorphic devices. Recent advances in solution processing, doping, and wafer-scale synthesis have further improved their performance and manufacturability [64, 65].

Inspired by brain-like computation, these technologies integrate memory and processing (in-memory computing, IMC) to overcome the von Neumann bottleneck. Hardware-based ANNs facilitate IMC, providing efficient platforms for machine-learning algorithms, while spiking neural networks (SNNs), which encode information in spike patterns, show promise for continuous real-time sensory processing in applications like edge computing (refers to performing data processing locally, near sensors or devices, instead of in remote cloud servers, thereby reducing latency and energy use in real-time neuromorphic operations), personalized medicine, and IoT.

Neuromorphic hardware development can broadly be categorized into three stages:

(i) *Current Large-Scale Neuromorphic Systems* Today’s hardware is primarily CMOS-based, using digital or mixed-signal designs. Examples include IBM’s TrueNorth, Intel’s Loihi, Tianjic, and ODIN, where neuron and synapse functions are emulated via CMOS transistors, capacitors, and SRAM memory. Researchers are actively using these systems to explore AI algorithms [66].
(ii) *Non-Volatile Memory Technologies* Recently, non-volatile memory (NVM) technologies such as RRAM, PCM, ferroelectric memory (FeRAM), FeFET, and MRAM have emerged as compact, low-power elements for synaptic nodes and neuron models, with potential to replace SRAM. Initially developed for data storage, these NVMs are now explored for neuromorphic computing. Hybrid CMOS/NVM circuits, as well as charge-based NVMs like flash and NRAM, are also under investigation for IMC. Additionally, technologies such as ferroelectric tunnel junctions (FTJ) and three-terminal electrochemical RAM (ECRAM) are being explored for IMC and neuromorphic applications [67].
(iii) *Future Memristive Materials and Emerging Computing Concepts* Current research is expanding into advanced materials, including 2D materials, organic compounds, perovskites, nanotubes, and nanowire networks, along with spintronic devices (e.g. domain-wall memory, race-track memory, skyrmions) and metal-insulator transition devices (e.g. VO_2_-based systems) [68–70]. These technologies remain in the proof-of-concept stage, mostly demonstrated at the single-device level or within small circuit blocks, where hardware–software simulations showcase their potential. Research also includes photonic component integration, paving the way for neuromorphic photonic processors on silicon photonic platforms with co-integrated optical memory and phase-change materials [71–73].

### Motivation

The rapid evolution of data-centric applications, particularly AI, is fundamentally reshaping technological landscapes across multiple fields of research. As AI and machine learning expand into new fields, they are driving unprecedented levels of research, development, and practical deployment. These advancements promise innovative solutions to longstanding challenges in sectors such as scientific research, education, transportation, urban planning, healthcare, and virtual environments. However, the pursuit of enhanced AI performance often overlooks critical aspects such as energy consumption and environmental impact. Considering the dependence of AI on extensive computing infrastructure, there is an urgent need to balance performance with energy efficiency, to ensure sustainable growth [1–3, 20, 22, 43].

**Fig. 3:**
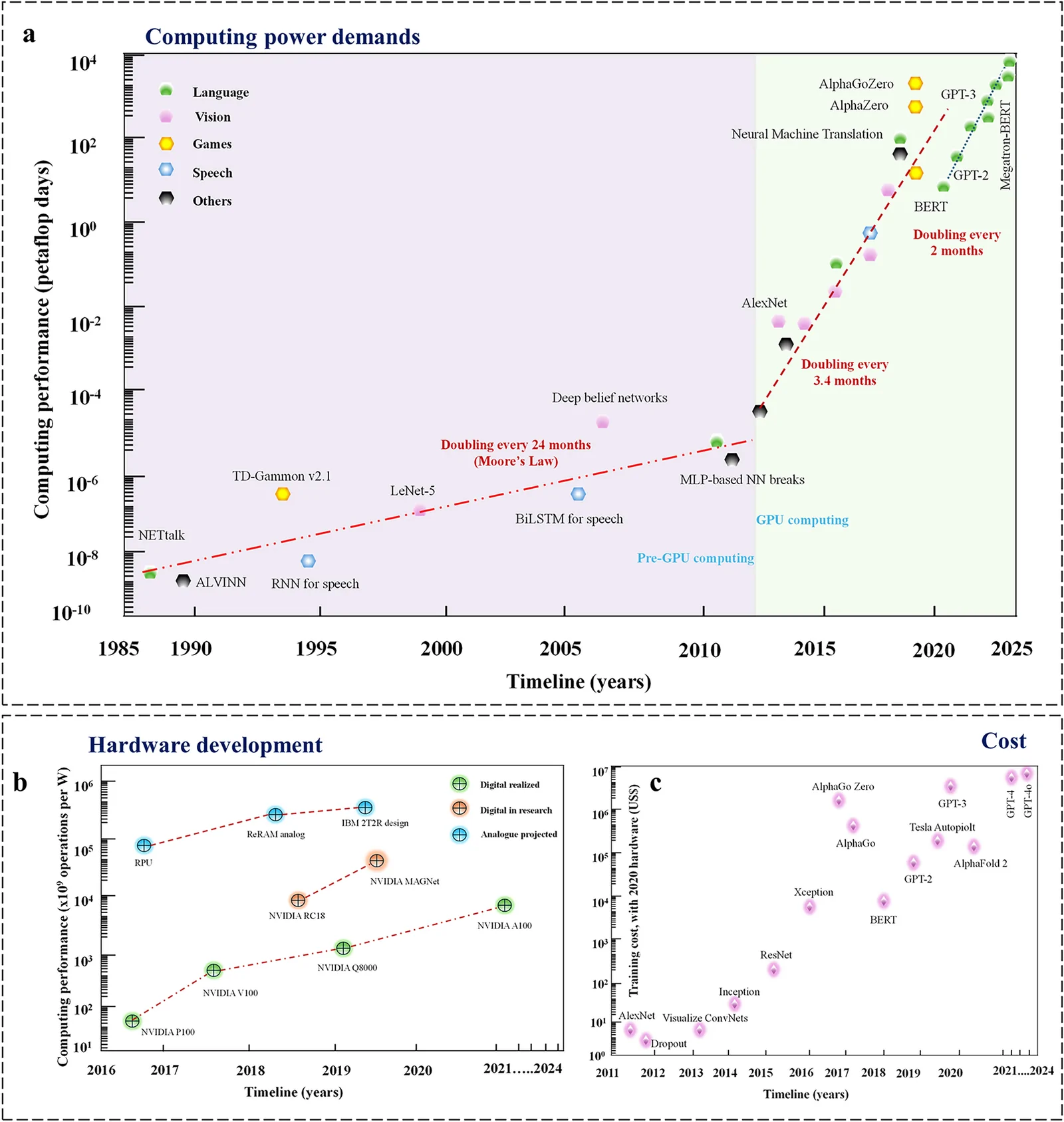
**a** The growth in computing power demand over the last four decades, measured in petaflops per day. Up until 2012, the demand doubled every 24 months, but this interval has now shortened to roughly every two months. Different application domains are indicated by the colour key. **b** Advancements in AI hardware efficiency over the past eight years, showing a more than 300-fold improvement due to cutting-edge solutions. Ongoing research and development efforts aim to achieve even greater gains. **c** Rise in AI model training costs since 2011, illustrating an exponential increase that is unsustainable in the long term

The rapidly increasing computational demands associated with large-scale AI tasks underscore the limitations of current hardware systems, highlighting the need for alternative approaches that can support further advancements in AI while remaining energy-efficient (Fig. 3). Figure 3a shows a dramatic rise in computing power requirements, quantified in petaflops per day, with computational needs doubling every two months—a rate that far surpasses traditional improvements dictated by Moore’s law. This exponential demand challenges the limits of semiconductor scaling, necessitating architectural innovations and co-designed hardware–software systems. For instance, NVIDIA’s GPUs achieved a 317-fold performance improvement between 2012 and 2024, though this came at the cost of increasing power consumption from roughly 25 to 320 W over the same period (Fig. 3b). While impressive gains have been made at the research and development stage [74, 75], it is increasingly evident that conventional computing alone cannot meet the long-term demands of AI, especially when considering the high costs associated with training complex models (Fig. 3c) [66].

When resistive switching (RS) devices are arranged in a crossbar configuration [76–78] conceptually, these devices resemble synapses in biological systems, where the two electrodes act as axon and dendrite connections, while the conductance of the switching layer represents the synaptic weight. When the crossbar network receives input pulses with amplitudes proportional to the values of an input vectorx, the current through each crosspoint cell reflects the multiplication of the input ${x}_{\mathrm{i}}$ and the conductance ${G}_{\mathrm{i}\mathrm{j}}$ of that cell. The total current (or charge) collected at column j is given by $I_{{\mathrm{j}}} = \mathop \sum \limits_{i} x_{{\mathrm{i}}} G_{{\mathrm{i}}}$, as per Kirchhoff’s current law and Ohm’s law. This parallel processing capability enables vector–matrix multiplication within the memory, eliminating the need for extensive data movement and greatly enhancing energy efficiency [55, 79].

In RS devices, the switching behaviour arises from ion redistribution within the switching layer, often involving oxygen vacancies (${V}_{\mathrm{o}}$) or metal cations. When a voltage is applied, ${V}_{\mathrm{o}}$ may drift in the direction of the field, potentially causing localized heating that accelerates drift and diffusion. Even after stimulation ceases, ion diffusion can continue, leading to complex dynamics that reflect the device’s memory of past inputs. Like biological synaptic plasticity, the conductance of RS devices can be fine-tuned by controlling voltage pulses, enabling them to replicate both short- and long-term synaptic behaviours [21, 79, 80]. This ability to emulate the cellular and molecular dynamics of neurobiological circuits forms the basis for artificial neural networks capable of advanced learning functions, positioning RS devices as a pivotal technology in neuromorphic computing.

There is a surge of global funding between traditional digital AI and neuromorphic computing technologies. Investments in conventional AI are substantial, with countries like China and the USA allocating billions to advance AI capabilities. For instance, China has committed nearly $8 billion to both civil and military AI, while the USA invested $973 million in civil AI alone in 2020. Other countries, such as the UK, France, and Germany, have made similarly substantial commitments to digital AI. However, comparable investments in neuromorphic technology remain sparse, primarily due to its nascent stage and lack of widespread strategic focus [81–83]. Despite the projected growth of the neuromorphic chip market from $22.7 million in 2021 to $550.6 million by 2026, funding still trails far behind that of digital AI and quantum computing. This funding gap highlights the need for increased support if neuromorphic technologies are to realize their potential in addressing AI’s rising energy demands [84].

To systematically address these challenges and objectives, the remainder of this review provides a comprehensive framework that explicitly links 2D material properties, device architectures, machine-learning modes, and target applications. Section 1.3 maps the scope and objectives of the review, ensuring a cohesive analysis of current limitations and establishing a clear roadmap for the future of 2D-material-enabled neuromorphic hardware.

### Scope and Objectives

The scope of this review is anchored in the exploration of emerging 2D materials, particularly focusing on their integration into machine learning-enabled systems for neuromorphic computing applications. Over the last five years, considerable advancements have been reported in using 2D materials for developing artificial synapses and neuromorphic devices. The review aims to provide an in-depth analysis of these advancements, highlighting the latest synthesis techniques, material properties, and device architectures that facilitate the integration of 2D materials into systems exploiting machine learning.

The necessity for this review is underscored by the critical limitations of traditional von Neumann architectures, particularly concerning energy inefficiency and scalability challenges. Current neuromorphic systems aim to address these limitations by exploiting 2D materials’ distinct properties, which hold promise for achieving low-energy, high-efficiency processing. This review thus examines how the integration of 2D materials into neuromorphic platforms could provide a viable path toward sustainable and scalable AI applications.

Key objectives of this review include:

- **Evaluation of 2D Material Properties for Neuromorphic Applications** This review will detail the intrinsic properties of emerging 2D materials that make them promising candidates for neuromorphic computing. It will examine how these properties, such as tuneable bandgaps, high mobility, and structural flexibility, enable the development of artificial synapses capable of mimicking biological functions like LTP and STP. Understanding these properties is essential for designing devices that can replicate the dynamic processes found in biological neural systems and mapping 2D materials’ neuromorphic capabilities to known ML models (e.g. STDP for SNN, analogue gain for CNN, memory states for RNN).
- **Integration with Machine-Learning Frameworks** Rather than providing a general overview of neuromorphic machine learning, this review delivers a 2D material- and device-specific analysis. We will explore how the unique physical dynamics of 2D materials are structurally integrated to execute and optimize specific machine-learning algorithms directly in hardware, thereby paving the way for adaptable, highly energy-efficient AI systems.
- **Advancements in Synaptic Device Architectures** We aim to provide an in-depth overview of recent progress in synaptic device architectures, including optoelectronic synapses, memristors, and phase-change memory. We will highlight how 2D materials have been incorporated into these configurations to achieve faster processing speed, higher efficiency, and scalability. The use of light-based signals in optoelectronic synapses is considered a breakthrough for achieving faster data processing and response times in neuromorphic systems.
- **Scalability and Integration Techniques** A key challenge in neuromorphic computing is the scalable synthesis and integration of high-quality 2D materials. This review will discuss recent advancements in chemical vapour deposition (CVD), atomic layer deposition (ALD), and other synthesis techniques that facilitate wafer-scale integration of 2D materials.
- **Benchmarking, Limitations, and Future Prospects** To establish a systematic structure and ensure overall coherence, this review introduces a comprehensive summary framework (Table 1) that explicitly links “material properties–device architecture–learning mode–application”. This framework serves as the structural backbone of the review, guaranteeing that individual performance metrics are contextualized within broader system-level goals. Despite significant progress, challenges such as material stability, defect management, device-to-device variability, endurance limitations, and performance optimization remain. This review will critically assess these limitations, proposing strategies such as advanced defect engineering, interface optimization, heterostructure design, and protective encapsulation to enhance device longevity and stability. Furthermore, the prospects of 2D-material-based neuromorphic devices will be discussed in the context of emerging applications, including autonomous systems, healthcare monitoring, wearable technologies, brain–machine interfaces, self-powered intelligent systems, and quantum-inspired neuromorphic computing. Particular emphasis will also be placed on benchmarking different material systems and device architectures in terms of energy consumption, switching speed, retention, endurance, scalability, and compatibility with machine-learning paradigms such as SNNs, CNNs, and RNNs. Among the different classes of 2D materials, TMDs are attractive because of their tunable bandgaps, strong optical response, and defect engineering flexibility, while h-BN is particularly promising for dielectric integration because of its low leakage current and high breakdown strength. BP offers superior carrier mobility and anisotropic transport, although its poor environmental stability remains a major challenge. Emerging materials such as tellurene, silicene, and other Xenes provide high mobility and multifunctional properties, but their synthesis and large-area integration are still relatively immature. Therefore, a critical comparison of material properties, scalability, reliability, and CMOS compatibility is essential for identifying the most suitable platforms for neuromorphic applications.

**Table 1:** Systematic framework of 2D-material-enabled neuromorphic computing

Material system & key properties	Device architecture	Machine learning mode/Algorithm	Target application
**TMDs (e.g. MoS**_**2**_**, WSe**_**2**_**)** Tuneable bandgap, strong optical response, structural flexibility, excellent defect engineering	**Optoelectronic synapses/memtransistors** Light-responsive multiterminal devices; photo-tuneable synapses	**Convolutional neural networks (CNNs) & SNNs** Analogue weight updates; Spike-Timing-Dependent Plasticity (STDP) for visual data	**Neuromorphic vision** Image recognition, optical parallel processing, and smart edge-computing sensors
**Hexagonal boron nitride (h-BN)** Large bandgap, high breakdown strength, ultra-low leakage current, excellent dielectric	**Two-terminal memristors (RSMs)** Resistive switching crossbar arrays; vertical metal–insulator-metal (MIM) structures	**Deep neural networks (DNNs)** Supervised learning via backpropagation; reliable deterministic switching	**High-density in-memory computing** Large-scale data storage, pattern recognition, and robust cloud-based AI accelerators
**Black phosphorus (BP) & alloys** Superior carrier mobility, strong anisotropic transport, broadband optical absorption	**Polarization-sensitive phototransistors** Anisotropic synaptic devices; multiterminal architectures (e.g. b-AsP)	**Spiking neural networks (SNNs)** Direction-selective learning; Long-Term Plasticity (LTP); paired-pulse facilitation	**Advanced optical sensing** Polarization-resolved imaging, 3D target reconstruction, and directional motion detection
**Emerging xenes (e.g. Tellurene)** High mobility, multifunctional piezoelectric/thermoelectric properties, high mechanical flexibility	**Flexible 3-terminal memtransistors** Gate-tuneable synapses; flexible substrate integration	**Recurrent neural networks (RNNs)** ime-series data processing; reinforcement learning; continuous state mapping	**Wearable & bio-integrated tech** Continuous healthcare monitoring, self-powered intelligent systems, and brain–machine interfaces

## Fundamentals of Neuromorphic Computing

### Principles and Architecture

Neuromorphic systems are structured to embody computational models inspired by the human brain, where the interconnection of neurons and synapses enables parallel, efficient, and adaptive information processing. At the core of these architectures lies a spiking-based communication model, in which information is conveyed through discrete, temporally precise events known as action potentials or “spikes”. This spike-based signalling, governed by principles such as STDP, allows neuromorphic networks to adapt dynamically by modulating synaptic strengths in response to the temporal correlation between pre- and post-synaptic spikes. Such a model permits local learning and adaptation, enabling neuromorphic systems to adjust to input patterns in real time and develop functionality that mimics experience-dependent learning in biological neural networks [85].

Architecturally, neuromorphic systems implement STDP through specialized hardware components, including two-terminal resistive switching devices and three-terminal transistor-based memory elements. These devices embody synaptic weights, where adjustments in conductance mirror synaptic potentiation or depression. The direct mapping of computation within memory structures, often referred to as in-memory computing, circumvents the data movement bottleneck characteristic of von Neumann architectures, thereby achieving heightened computational efficiency. In neuromorphic arrays, spike-based data processing inherently minimizes energy consumption by localizing computations, making these architectures particularly well-suited for edge computing, real-time sensory systems, and adaptive control applications.

By aligning hardware operations with principles observed in biological networks, neuromorphic architectures emulate brain-like efficiency and simultaneously introduce scalable platforms for computational tasks that conventional digital processors struggle to handle. The principles underpinning these systems represent a radical shift, moving beyond traditional standards of clock-driven computation toward asynchronous, event-based processing that holds promise for next-generation artificial intelligence and adaptive learning systems.

### Integration of Machine Learning

The convergence of neuromorphic computing and ML represents a paradigm shift in computational science, particularly in the pursuit of systems capable of adaptive learning and energy-efficient data processing. While traditional ML models have primarily been developed and optimized on digital, von Neumann-based architectures, their computational demands are increasingly unsustainable as model complexity grows. Neuromorphic systems offer a promising alternative, where the intrinsic properties of biological inspiration, such as event-driven processing and in-memory computation, provide a foundation for developing hardware-accelerated machine learning that is both highly efficient and adaptable [86]. Importantly, not all ML-assisted neuromorphic demonstrations represent the same degree of hardware realization. In this review, studies are distinguished into five categories: (i) software-level or numerical simulations, where device behaviour is modelled computationally; (ii) device-informed simulations, where experimentally measured device parameters are incorporated into ANN, CNN, RNN, or SNN frameworks; (iii) hardware-assisted inference, where trained models are executed using neuromorphic hardware; (iv) hybrid experimental/computational systems, where device measurements are combined with external training or inference algorithms; and (v) true in situ or on-chip learning, where synaptic weights are updated directly within the hardware itself. This classification is important for distinguishing proof-of-concept algorithm validation from genuine hardware-level learning in 2D-material-based neuromorphic systems.

#### Neuromorphic Adaptations for ML Algorithms

Recent advances have enabled the mapping of machine-learning (ML) models, especially spiking neural networks (SNNs), onto neuromorphic hardware to exploit event‑driven, asynchronous computation. Unlike dense, clock‑based computation used in conventional neural networks, SNNs communicate via sparse spikes, significantly reducing energy associated with data movement and computation.

Quantitative evaluations show substantial energy efficiency gains in neuromorphic hardware relative to GPU implementations for specific workloads. For example, memristor crossbar neural accelerators can reduce energy per inference by an order of magnitude or more compared to GPUs [87]. Event‑driven processors such as Intel’s Loihi show reductions in energy per synaptic operation relative to GPU implementations for spiking tasks (Davies et al., “Loihi: A neuromorphic manycore processor with on‑chip learning”) [88]. Similarly, IBM TrueNorth demonstrated low power consumption in spiking benchmarks, emphasizing the energy benefit of event‑driven designs [89]. In particular, device characteristics such as STDP, spike-frequency adaptation, and short-term or long-term plasticity are highly relevant for SNN implementation because they directly emulate temporal spike processing and synaptic weight updating. For example, MoS_2_- and h-BN-based synaptic devices have demonstrated STDP behaviour that can be directly mapped onto SNN frameworks for pattern recognition and event-driven sensing. In addition, most early demonstrations of SNN implementation in neuromorphic systems rely on software-based simulations or hardware-assisted inference rather than fully on-chip training. Therefore, the reported recognition accuracies often depend strongly on the dataset, model architecture, and external training protocol used [90, 91].

#### On-Chip Learning for Real-Time Adaptation

One of the compelling aspects of integrating ML with neuromorphic systems is the ability to perform on-chip learning. Conventional ML implementations often separate the training and inference phases due to the prohibitive computational costs associated with real-time training on von Neumann architectures. Neuromorphic computing, however, supports continuous, local learning through mechanisms such as STDP and other forms of Hebbian learning, allowing synaptic weights to update dynamically in response to temporal patterns in data. This approach enhances the adaptability of neuromorphic systems in real-world, unpredictable environments and reduces the need for data transmission, an energy-intensive process in traditional systems. Recent research demonstrates that integrating reinforcement learning frameworks directly onto neuromorphic chips enables agents to adapt to new tasks and environmental changes without requiring external computational resources [92]. These local learning mechanisms are particularly important for 2D-material-based neuromorphic devices, where conductance modulation, retention behaviour, and synaptic plasticity can directly support adaptive on-chip learning. In contrast to hardware-assisted inference, true on-chip learning requires direct updating of synaptic weights within the device itself. Such demonstrations remain relatively limited in 2D-material-based systems because of challenges associated with device variability, retention, and non-ideal conductance updates [93, 94].

#### Efficient Implementation of Deep-Learning Architectures

Neuromorphic systems support event‑driven processing and can adapt many deep-learning architectures to this paradigm. For CNNs, analogue conductance modulation and linear weight updates are vital for multiply‑accumulate accuracy. Material systems such as ferroelectric synapses and optoelectronic devices demonstrate gradual conductance change and long retention suitable for CNN inference [95]. Recent neuromorphic architectures emulate such temporal dynamics using internal delay lines, spike integration, or even photonic memory components. By exploiting sparsity, local connectivity, and in-memory computation, these models have been adapted to neuromorphic systems, achieving comparable accuracy with substantially lower energy demands. Techniques such as quantization, pruning, and hybrid SNN-ANN (artificial neural network) models have been instrumental in translating deep-learning tasks to neuromorphic platforms. Studies from recent years indicate that neuromorphic implementations of CNNs for image recognition and RNNs for time-series prediction achieve state-of-the-art efficiency, setting new benchmarks for power consumption and latency in embedded and IoT applications [1, 2, 29–31, 33–37, 86, 95–98]. However, these reported efficiencies are often obtained from hybrid hardware–software demonstrations or device-informed simulations rather than fully integrated hardware systems. Therefore, recognition accuracies should be interpreted in the context of the dataset used, the network architecture, the training method, and whether the results were experimentally demonstrated or numerically simulated.

Building on these advancements, recent developments have introduced the integration of diffractive processing units (DPUs) within RNN frameworks, further enhancing the efficiency of optoelectronic computing [99]. The diffractive recurrent neural network (D-RNN) architecture exemplifies this approach, where each layer incorporates memory states through diffractive optical elements that modulate and retain temporal information across sequences. This structure supports real-time adaptability, making it particularly suitable for dynamic applications like video sequence analysis and human action recognition [95]. Although the D-RNN demonstrates high sequence recognition accuracy, the reported performance is primarily based on a hybrid optoelectronic-computational framework rather than fully autonomous hardware-level learning.

The experimental results from the D-RNN demonstrate impressive sequence accuracy on benchmark datasets, including the Weizmann and KTH databases. For instance, in the Weizmann dataset, categories 0 to 9 represent actions such as bending, jumping, and running, while the KTH dataset includes actions like boxing, jogging, and hand-waving. Through this architecture, the D-RNN was able to achieve high sequence recognition accuracy across both datasets. By integrating optoelectronic components within deep-learning architectures, the D-RNN model sets a new benchmark for energy efficiency and performance in neuromorphic computing, highlighting the potential of hybrid approaches that combine optics and electronics to advance the field of adaptive neuromorphic learning [36, 98, 100]. These examples highlight the importance of hardware–algorithm co-design, where material properties and device functionalities are tailored for specific ML tasks.

#### Machine-Learning Models Optimized for Neuromorphic Hardware

As ML and neuromorphic computing continue to intersect, there is growing interest in developing algorithms explicitly optimized for neuromorphic hardware. The concept of spike-based backpropagation has gained traction, where training occurs directly within spiking networks, sidestepping the need for conventional gradient-based methods that are computationally taxing on neuromorphic systems. Emerging algorithms such as spike-timing-dependent error backpropagation (STDEBP) and temporal contrastive divergence enable efficient weight updates in spiking networks, preserving the biological plausibility and energy advantages of neuromorphic hardware. These algorithms have demonstrated near-equivalent performance to traditional backpropagation for various cognitive tasks, positioning them as foundational tools for neuromorphic-based ML models [37]. The development of such algorithms is particularly important for 2D-material-based neuromorphic hardware, as these devices often exhibit nonlinear switching, multilevel conductance states, and stochastic behaviour that differ from conventional digital systems. Despite these advances, many spike-based training algorithms are still validated using numerical simulations or simplified device models. Demonstrating these algorithms directly in large-scale 2D-material-based hardware remains an important challenge for the field.

#### Applications and Future Directions

The integration of neuromorphic hardware with ML algorithms opens transformative possibilities in applications that require adaptive, real-time processing and minimal energy consumption. In healthcare, for instance, neuromorphic-ML systems could enable continuous, low-power monitoring of physiological signals, adapting dynamically to detect anomalies. In autonomous vehicles and drones, neuromorphic architectures could facilitate fast, energy-efficient perception and decision-making, enhancing safety and responsiveness. The potential for large-scale deployment in smart cities, environmental monitoring, and wearable technology continues to drive research in this area, with recent developments indicating rapid progress toward fully integrated neuromorphic-ML ecosystems that balance computational power with energy efficiency [34, 36, 37]. Overall, these emerging applications demonstrate how neuromorphic hardware, machine-learning algorithms, and 2D material properties can be integrated within a unified framework for adaptive and energy-efficient computing.

## Overview of Emerging 2D Materials for Neuromorphic Applications

### Transition Metal Dichalcogenides: Characteristics and Applications in Neuromorphic Devices

Among the category of 2D materials, 2D transition dichalcogenides are unique systems with exceptional structures of MX_2_, where X is the chalcogen atoms [101]. MX_2_ consists of a transition metal layer sandwiched between two chalcogen layers, with M between two chalcogen atoms. In the monolayer of TMDs, the constituent atoms of chalcogens are covalently bonded with the transition metal, forming the structure X-M-X, whereas in the bulk, these monolayers stack together through weak van der Waals (vdW) interactions, forming a layered crystal structure [46, 102]. Based on the combination of transition metals with chalcogen, the 2DTMDs exhibit metals (NbS_2_, T1S_2_), semimetals (PolTe_2_, PfTe_2_) and semiconductors (MoS_2_, WS_2_) [103–105]. This wide range of conductivity of 2DTMDs provides an excellent and unprecedented platform to fabricate and complete all 2D TMDs-based memristors and synaptic devices. In addition, the polymorphs of semiconducting 2D TMDs, such as 2H-, It-, and 1 T’—MOS_2_ with different structures, further lay down a platform for the active layer/switching layers in memristor and neuromorphic devices [100]. For a memristor and neuromorphic device to qualify as an artificial synapse, it must show analogue changes in resistance, analogously in its vertical structure (as a memristor) and lateral structure as a memtransistor [106–109].

The resistance of 2D TMDs can be modulated by movement or displacement of a chalcogen vacancy, heterojunctions doping and alloying transition metal atoms [110]. Hence, these materials serve as excellent active layers for memristors and neuromorphic devices because their atomically thin geometry allows precise control of defect formation and ion migration pathways, while their tunable bandgaps and high carrier mobility enable stable resistive switching and efficient synaptic modulation [110]. The digital and analogue switching in WSe_2_-based devices was achieved by applying higher and lower voltages, respectively, which has been proved to be caused by the migration and accumulation of Se vacancies. In another report, the reversible modulation of MOS_2_ films that consist of local 2H-1 T’ phase position is controlled by the input of Li^+^ Ion with an electric field. Where an increase/decrease in local Li^+^1a contraction leads to the transition between 2H (semiconductor) and 1 T’ phase [110], the memtransistor devices of WS_2_-based neuromorphic devices have demonstrated synaptic operation at high temperatures [111]. The device showed endurance greater than 100 cycles with pulse programmable memory ranging over six orders of magnitudes (10^–12^–10^−6^A). The 2D TMDS conveyed exceptional artificial photoelectric synaptic behaviour. Another study [112] demonstrated the photoelectric synapse’s ability to mimic the human visual system by employing Lewis’s acid-doped semiconducting tungsten diselenide (WSe_2_). The device consumed around 0.1 fJ per synaptic operation, a value approximately 100 times lower than the energy required for a single synaptic event in the human brain (≈10 fJ)—and also lower than that of most presently reported WSe_2_-based synaptic devices. To demonstrate the chalcogenide base in memory computing concepts, Mingjun et al. [112] fabricated an ultrathin optomemristor: The Ge-doped selenide photoactive cavities could emulate synapses with 3-factor Hebbian plasticity and dendrites with shunting inhibition. The heterojunction of these 2D TMDs also plays a key role in demonstrating non-volatile memory and synaptic simulations. Huang et al. [112] reported a planar optical and electrical tuneable memristor based on a Sn/WS_2_ van der Waal heterostructure. The device demonstrated a unipolar switch with R_on_/R_off_ = 10^6^, with durable endurance and retention superior to individual films of ReS_2_ and WS_2_. The device showed further switching modulation by optical illumination, which was believed to be caused by the interlayer charge transfer. Sattar et al. [113] report the first integration of MXene (Ti_3_C_2_/V_2_C) with MoS_2_ to create vertical memristors, achieving low SET voltages (0.6 V), excellent endurance (3000 cycles), and retention (> 10^3^ s). The study demonstrates a 5 × 5 memristor array with 18 reproducible devices and showcases synaptic potentiation and depression behaviour. This paper significantly advances the understanding of MXene-MoS_2_ heterostructures in scalable and high-performance memristors, and is particularly relevant to our discussion on TMD-based memristors and their potential for large-scale integration. Bhunia et al. [114] present a MoTe_2_-based synaptic-bridge memristor fabricated using a solution-processed 2H-MoTe_2_:PVA (3:1) composite, which demonstrates stable bipolar switching with minimal voltage variation over 125 cycles. The device emulates essential synaptic behaviours including STDP, SNDP, LTP, and LTD, along with higher-order functionalities such as Pavlovian learning and Morse code recognition. The authors further evaluate the device’s neuromorphic potential through simulations using MLP and CNN frameworks, achieving competitive image classification accuracy on the CIFAR-10 dataset. This paper expands our TMD section by introducing a less-explored MoTe_2_-based system [115].

**Fig. 4:**
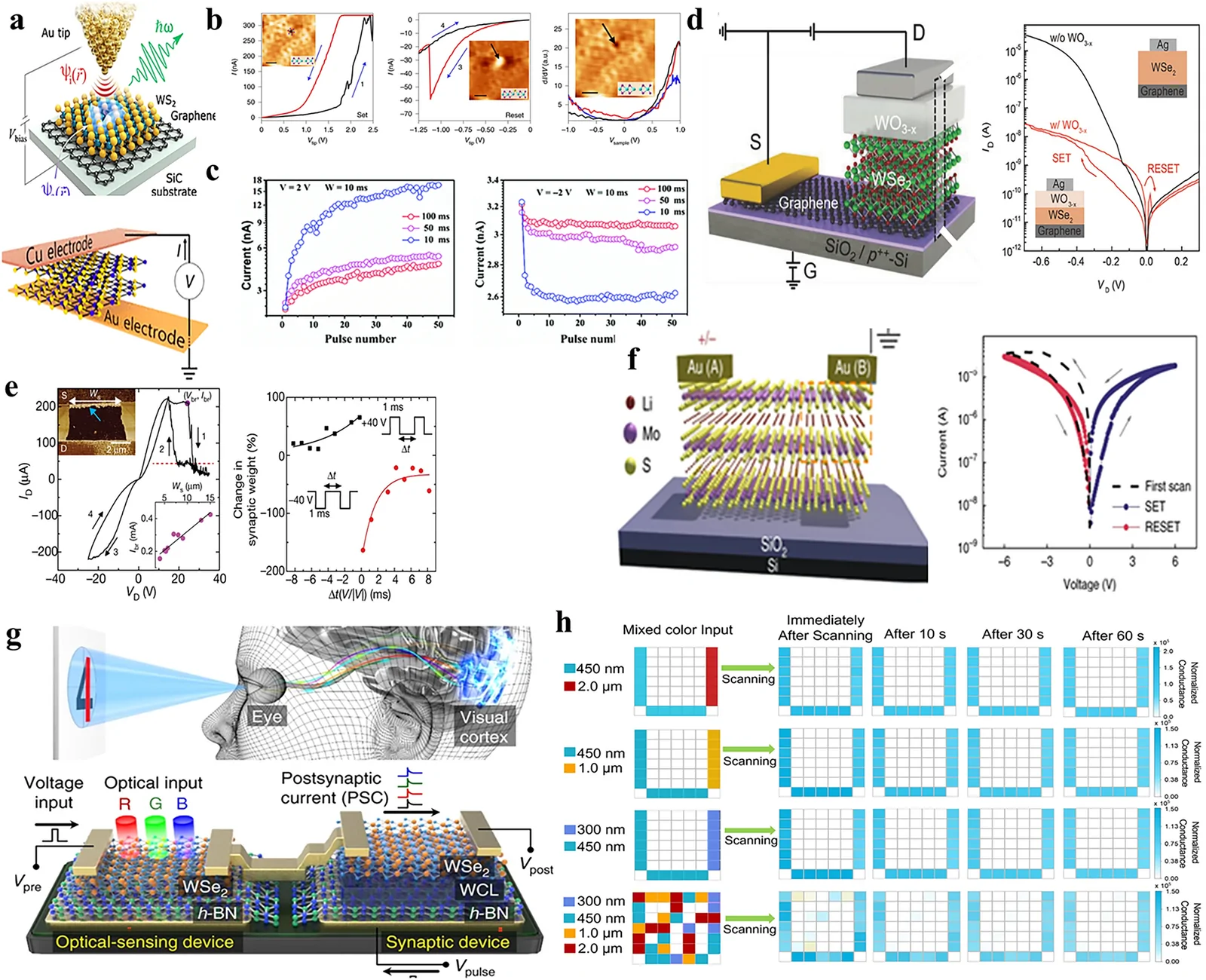
Structure and synaptic characteristics of 2D TMDs. **a** Schematic illustration of TMD layers with possible intrinsic defects and their applications in synaptic applications. Reproduced with permission from Ref. [116–120]. **b** Observation of critical role played by single sulphur vacancy (VS_2_) in an MoS_2_ atomic sheet memristor by scanning tunnelling microscopy. Reproduced with permission from Ref. [121]. **c** Synaptic characteristics exhibited by two-dimensional layers of WSe_2_ nanosheet. Reproduced with permission from Ref. [122]. **d** Schematic illustration of Ag/WO_x_/WSe_2_/graphene heterostructure barrister with corresponding I–V characteristics well-suited to mimic neuronal-based synapse. Reproduced with permission from Ref. [123]. **e** Atomic force microscope topographic images of polycrystalline monolayer MoS_2_-based memtransistor, its electrical characteristics and the spike time dependent plasticity achieved by the device. Reproduced with permission from Ref. [124]. **f** Schematic diagram of synaptic device and phenomenon depicting the controlled 2H-ITʹ phase transition ion modulated MoSe_2_-based device, its I–V characteristics and conductance change through pulse programming. Reproduced with permission from Ref. [125]. **g** Optical neural synaptic device consists of hybrid structured h-BN/WS_2_ and testing of dataset for single and coloured images. Reproduced with permission from Ref. [29]. **h** Self-denoising with mixed colour input of MoS_2_ transistor with p-Si/PtTe buried electrodes. Reproduced with permission from Ref. [96]

Figure 4 illustrates the synaptic applications of devices based on 2D TMDs. The flexible nature of these materials, combined with their intrinsic defects—such as chalcogen vacancies, doping through substitution, grain boundaries, and multistacking capabilities or heterostructure formation enabled by dangling bonds—significantly enhances their versatility. These properties make 2D TMDs suitable for a wide range of device applications, including memristors, transistors, and photodetectors, as demonstrated in Fig. 4a [116–120]. The Chalcogen vacancies play vital roles in the switching behaviour of 2D TMDs when they act as switching layers of memristors. Hus et al. [24] prepared the monolayer on MoS_2_ on the freshly prepared gold surface. During the scanning tunnelling microscopy/scanning tunnelling spectroscopy (STM/STS) of the MoS_2_ monolayer, the gold layer acted as conducting substrate and bottom electrode to reveal the transport phenomenon during in situ characterization. Figure 4b [121] shows that when a voltage bias of 1.8 V is applied, the device switches from a high-resistance to a low-resistance state—a process known as the set event. This transition occurs because gold ions migrate from the substrate and occupy sulphur vacancies in the MoS_2_ lattice, thereby forming a more conductive path. The reverse bias voltage of −1.1 V removed the gold atom from the location of sulphur vacancy, and the MoS_2_ atomic sheet returned to its high-resistance state. The synaptic plasticity of CVD-grown WSe_2_ is shown in Fig. 4c, in which Lu et al. [122] demonstrated the typical bipolar switching characteristics. The newly fabricated Ag/WSe_2_/Ag structured memristors showed high endurance (6 × 10^3^) and retention up to 3.6 × 10^4^. The WSe_2_-based memristor successfully emulated the biological synaptic functions when the input pulses of different voltage heights and intervals were used as excitatory pre-synaptic pulses. The ability of the device to mimic the long-term and short-term plasticity and their inter-transition confirms the possible use of a WSe_2_-based memristor as an artificial neuron.

The 2D TMDs also contribute to the new class of artificial synaptic architecture. Huh et al. [123] demonstrated a synaptic barristor—a type of barrier transistor—based on an Ag/WO_x_/WSe_2_/graphene heterostructure, as shown in Fig. 4d. In this device, the Schottky barrier at the metal–semiconductor interface is tunable by an external electric field, allowing modulation of current flow in a manner analogous to biological synaptic plasticity. To further emulate biological learning, 2D TMDs have also been employed in three-terminal memristive devices—known as memtransistors—where a gate electrode modulates the channel conductance. Polycrystalline MoS_2_-based memtransistors exhibited excellent gate-tunable resistance states and demonstrated long-term potentiation (LTP) and depression (LTD) behaviours, as shown in Fig. 4e [124]. Moreover, a six-terminal MoS₂ transistor demonstrated gate-tunable hetero-synaptic functionality—capabilities such as multiinput signal integration and correlated plasticity—that cannot be realized in conventional two-terminal memristors. From the device modelling, in situ scanning probe microscopy and cryogenic charge transport measurements, it is revealed that the conductance variation is caused by the bias-induced motion of MoS_2_ defects. The local phase transition of 2D TMDs from the 2H-1 T′ phase also played a vital role in demonstrating the synaptic function, as shown in Fig. 4f [125]. Due to the controlled barrier height of the ultrathin van der Waal heterostructure by an external field, the device demonstrated the brain’s synaptic plasticity. Zhu et al. showed that the migration of Li^+^ can be controlled by the externally applied electric field in the planar structured Au/Li_x_MoS_2_ (~ 40 nm thick)/Au device. The increase and decrease in Li-ion concentration via the local ion migration results in the transition between the 1 T′ (metal) phases and 2H (semiconductor). The devices showed gradual increases (potentiation) and decreases (depression) in conductance when stimulated with 100 positive and 100 negative voltage pulses (± 4 V, 1 ms each), mimicking the learning and forgetting processes of biological synapses. The heterojunction of 2D TMDs also demonstrated the optical-sensing functions, as shown in Fig. 4g [29]. The heterostructure of h-BN/WSe_2_ successfully demonstrated the colour/colour mixed pattern recognitions of human vision. More appealingly, the device operated with a low voltage peak of 0.3 V and consumed 66 fJ per spike. More than 90% of colour pattern recognition is similar to the colour blindness test. A 7 × 7 array of phototransistors with a bilayer of MoS_2_ showed robustness to optical noise due to the interplay between long-term and short-term potentiation [96]. Figure 4h illustrates that the 7 × 7 array of MoS_2_-based synapses could still recognize the letter “U” even when the input pattern was distorted by coloured noise—demonstrating the array’s ability to automatically filter out optical interference, a behaviour referred to as self-denoising.

TMD-based neuromorphic devices are among the most extensively studied because they combine tunable bandgaps, strong optical absorption, and defect-mediated switching. However, their practical deployment remains limited by grain-boundary-induced variability, non-uniform vacancy distributions, and challenges in wafer-scale growth. Compared with h-BN and BP, TMDs provide better switching tunability and optical functionality, although their device-to-device reproducibility still requires significant improvement.

### Hexagonal Boron Nitride: Its Role as an Insulator and Gate Dielectric in 2D-Material-Based Devices

For the development of post-Moore electronic devices, 2D materials have been the central attraction since the rise of graphene [126–130] due to their layer-dependent optoelectronic and electrical properties with exceptional mechanical flexibility. Among these, 2D hexagonal Boron nitride (h-BN) with a structure resembling graphene has demonstrated excellent electronic properties when used either as a standalone monolayer or as part of van der Waals heterostructures combined with other 2D materials.

Bulk BN exhibits exceptional chemical and thermal stability, mechanical rigidity, and a low dielectric constant, making it attractive for diverse applications such as electronic packaging, neutron-detecting ceramic fillers, and water purification [131–133]. Boron nitride exists in three crystalline forms: cubic (C-BN), wurtzite (W-BN), and hexagonal (h-BN) [134, 135]. C-BN and W-BN both feature tetrahedral coordination through *sp*^3^ hybridization of B and N, with W-BN consisting of atomically close-packed B and N layers. In contrast, h-BN adopts an *sp*^2^-hybridized honeycomb layered structure. Within each h-BN monolayer, the highly polarized B–N covalent bond (length 0.145 nm) is strong, while adjacent layers interact via weak van der Waals forces with an interlayer distance of 0.334 nm [136, 137]. The large band gap (~ 6 eV), high breakdown strength, atomically thin nature, and excellent chemical and thermal stability of h-BN position it as an outstanding active medium for memristors and neuromorphic applications. Moreover, its defect-free van der Waals interface, low leakage current, and high dielectric strength offer excellent gate dielectric capabilities for memristors and synaptic devices [138]. Tej et al. [73] fabricated an atomically thin 2D h-BN-based memristor with ultra-short switching speed in the range 120 ps ~ 3 ns. Xe et al. [139] demonstrated hardware implementation of dot-product operations and analogue functions—ubiquitous in machine learning—using arrays of h-BN memristors. Wafer-scale h-BN memristors have also been fabricated via chemical vapour deposition. In another approach, Zhu et al. [140] reported inkjet-printed h-BN memristors, albeit with high device-to-device variability.

The inherent device-to-device variation—referring to the natural differences in switching thresholds and current levels among individual devices—introduces randomness that can be exploited for data encryption and hardware-based security in neuromorphic systems. The multilayer (5–7 layer) h-BN has been used as a switching medium in the memristive high-performance synapse by Shi et al. [141]. The memristive device successfully exhibited long-term and short-term plasticity when operating in volatile and non-volatile regimes.

The use of h-BN has been extended to its applications as a channel material in field-effect transistor-based synaptic devices. Shen et al. [142] prepared the layered h-BN using an industry-compatible method with chemical vapour deposition. In the fabricated transistor, the h-BN layer formed a clean, defect-free interface with the 2D semiconductor, minimizing charge trapping and hysteresis. Such stable interfaces enable reliable synaptic weight modulation in neuromorphic transistors, making h-BN a promising dielectric for mimicking brain-like signal transmission. Yan et al. [143] presented a Moiré Synaptics transistor based on an asymmetric bilayer graphene/h-BN-based Moiré heterostructure. Its bidirectional threshold sliding was suitable for implementing input-specific adaptation in neuromorphic hardware.

**Fig. 5:**
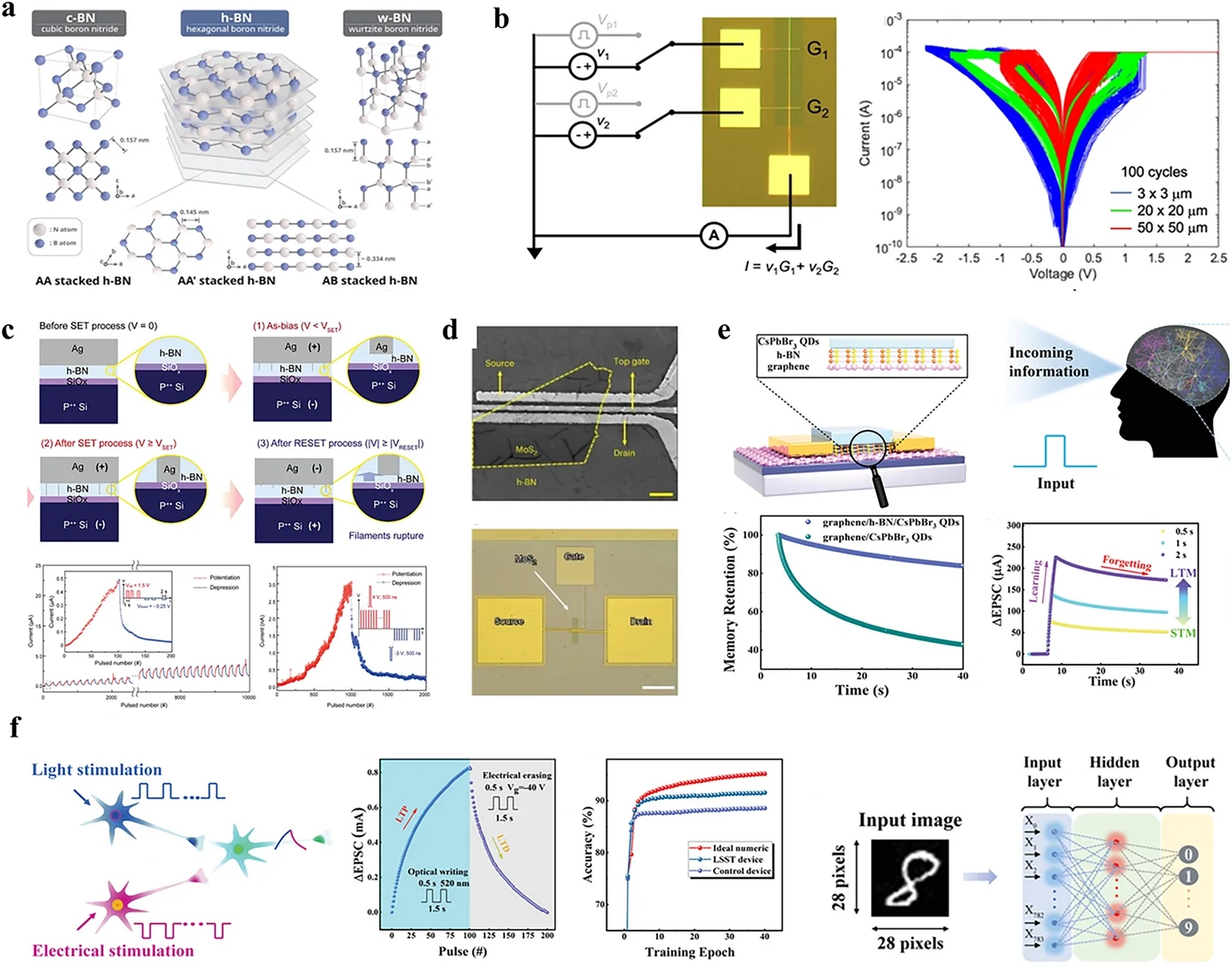
Structure and neuromorphic application of h-BN and its heterojunction devices. **a** Schematics of crystal structure of BN and the structural polytypes of h-BN. Reproduced with permission from Ref. [144]. **b** Top view optical image of h-BN-based crossbar array with its HRTEM cross-section visualizing the conducting paths. The 100 cycles of I–V characteristics of three different devices with different active areas and three different cases of pulse programming of the array. Reproduced with permission from Ref. [139]. **c** Proposed switching mechanism of the attojoule h-BN-based memristor and its potentiation and depression performance for the neuromorphic applications. Reproduced with permission from Ref. [145]. **d** h-BN dielectric for the transistor with its transfer curves in the dark and light environment. Reproduced with permission from Ref. [142]. **e** Light stimulated h-BN/graphene base synaptic transistor with its ability to train on the date and synaptic function for the artificial visual perception system. Reproduced with permission from Ref. [146]. **f** Optical writing performance for the pattern recognition of LLST device with 520 nm wavelength for the writing. Reproduced with permission from Ref. [146]

Figure 5a displays the crystalline structure of BN, which includes *c*-BN, h-BN and *w*-BN. For further insights, Fig. 5a compares the crystalline structures of cubic BN (c-BN), hexagonal BN (h-BN), and wurtzite BN (w-BN), highlighting their distinct bonding configurations and stacking sequences [144]. Xie et al. fabricated an Au/h-BN/Au array of memristors for analogue-based machine learning. The h-BN was grown by CVD, and different sizes of devices (3, 20, and 50 μm) were fabricated, as shown in Fig. 5b [139]. The different active areas of the memristor demonstrated reliable bipolar 100 I–V cycles with different set and reset voltages corresponding to the high-resistance state (HRS) and low-resistance state (LRS) of the device. The h-BN memristor array demonstrated the dot product, which is crucial for machine learning and neuromorphic hardware. The cross-sectional TEM shows a high-resolution image of Au/h-BN/Au with local defects contributing to the formation of conductive paths while demonstrating an ultra-low-energy memristor capable of analogue (continuously tunable) resistance modulation. Kim et al. designed a h-BN-based memristor structure metal-insulated-semiconducting (MIS) memristor as shown in Fig. 5c [145]. In this architecture, h-BN is facilitated as a switching layer, while the SiO_x_ on the Si substrate contributes to suppressing the current. The nanosecond fast memristor attained multilevel resistance states, resulting in the best candidacy in these devices for the next-generation neuromorphic application. Shen et al. employed h-BN as a dielectric of the transistor with different gate electrodes of high cohesive energy—platinum and tungsten, as shown in Fig. 5d [142]. For this purpose, the h-BN was grown using CVD and MoS_2_ was transferred to h-BN as a channel material. The transfer characteristics of the transistor were very responsive for gate voltages ranging from 0 to 4.0 V.

In contrast, the response distinguishes I_D_–V_D_ characteristics in the dark and under illumination, confirming its correct use as a photodetector and photo synapse. Han et al. [146] demonstrated the light-stimulated synaptic transistor for artificial visual perception with an ultrathin carrier regulator layer of h-BN into a graphene-based hybrid transistor. The light-stimulated synaptic transistor device demonstrated an ultra-high PPF index (nearly 196%) in the graphene/h-BN/perovskite heterostructure structure. To demonstrate the impact of h-BN on the ultra-high PPF index, another device without h-BN was fabricated in stacking graphene/perovskite structure. Benefiting from the high optical response, the various synaptic functions such as STM, LTM, short-term plasticity, and long-term potentiation have been achieved by LSST, as shown in Fig. 5e [146]. The LLST device further performed pattern recognition, in which optical writing was performed using a wavelength of light of 520 nm with a width of 0.5 s. In contrast, the electrical erasing of the device was carried out by V_g_ = − 40 V with a pulse width of 0.5 s and an interval of 1.5 s, as shown in Fig. 5f [146]. Compared with other 2D materials, h-BN is particularly attractive for neuromorphic devices because of its excellent dielectric properties, low leakage current, high breakdown strength, and strong CMOS compatibility. Nevertheless, the formation of conductive filaments in h-BN is often difficult to control, leading to variability in switching voltage and conductance states. Achieving large-scale uniformity therefore remains an important challenge.

### Black Phosphorus: Anisotropic Properties and Their Impact on Synaptic Behaviour

2D materials are derived from layered crystal structures. In these materials, the atoms within each layer are strongly bonded through covalent or ionic interactions (in-plane bonding), while weak van der Waals forces hold adjacent layers together (out-of-plane interactions) [134, 147, 148]. 2D materials with exceptional anisotropic properties have been studied extensively following the isolation of BP in 2014 [149–151]. In BP, atoms exhibit strong anisotropy within the crystal plane, resulting in direction-dependent electronic, thermal, and optical properties [152–154]. Motivated by this discovery, other layered 2D materials with similarly pronounced anisotropic characteristics have been explored in depth [155–157].

Thanks to its flexibility, compatibility with other 2D materials, and optical anisotropy, BP has been extensively used in the development of electronic devices, including photodetectors, memristors, transistors, and related applications [158–160]. Motivated by BP’s promising performance, various other anisotropic 2D materials have also been investigated for similar device applications [161].

Zhu et al. [30] developed a BP-based electronic device featuring a photodetector with multiple layers of BP serving as conductive channel. The unique photonic behaviour of this device enabled the demonstration of synaptic functions at significantly reduced power consumption. A fully connected optoelectronic neural network utilizing this technology achieved an impressive recognition accuracy of 94%.

Zhuo et al. [162] fabricated a transparent memristor based on BP nanosheets coated with polystyrene (PS) and sandwiched between indium tin oxide (ITO) electrodes. The device exhibited over 75% optical transmittance between 350 and 1100 nm and maintained stable bipolar resistive switching without an initial forming step, demonstrating its suitability for transparent and light-modulated neuromorphic electronics. Such a device could be beneficial in applications where transparency and optical modulation are critical.

Tan et al. [163] successfully demonstrated the first BP-based synaptic transistor in 2016. This device effectively emulated complex synaptic behaviours, such as LTP, LTD, and STDP. The synaptic behaviour was primarily attributed to charge transfer between the thin native oxide layer (~ 2 nm) on BP and the BP conductive channel. This advancement marked a significant milestone in neuromorphic device research using BP.

**Fig. 6:**
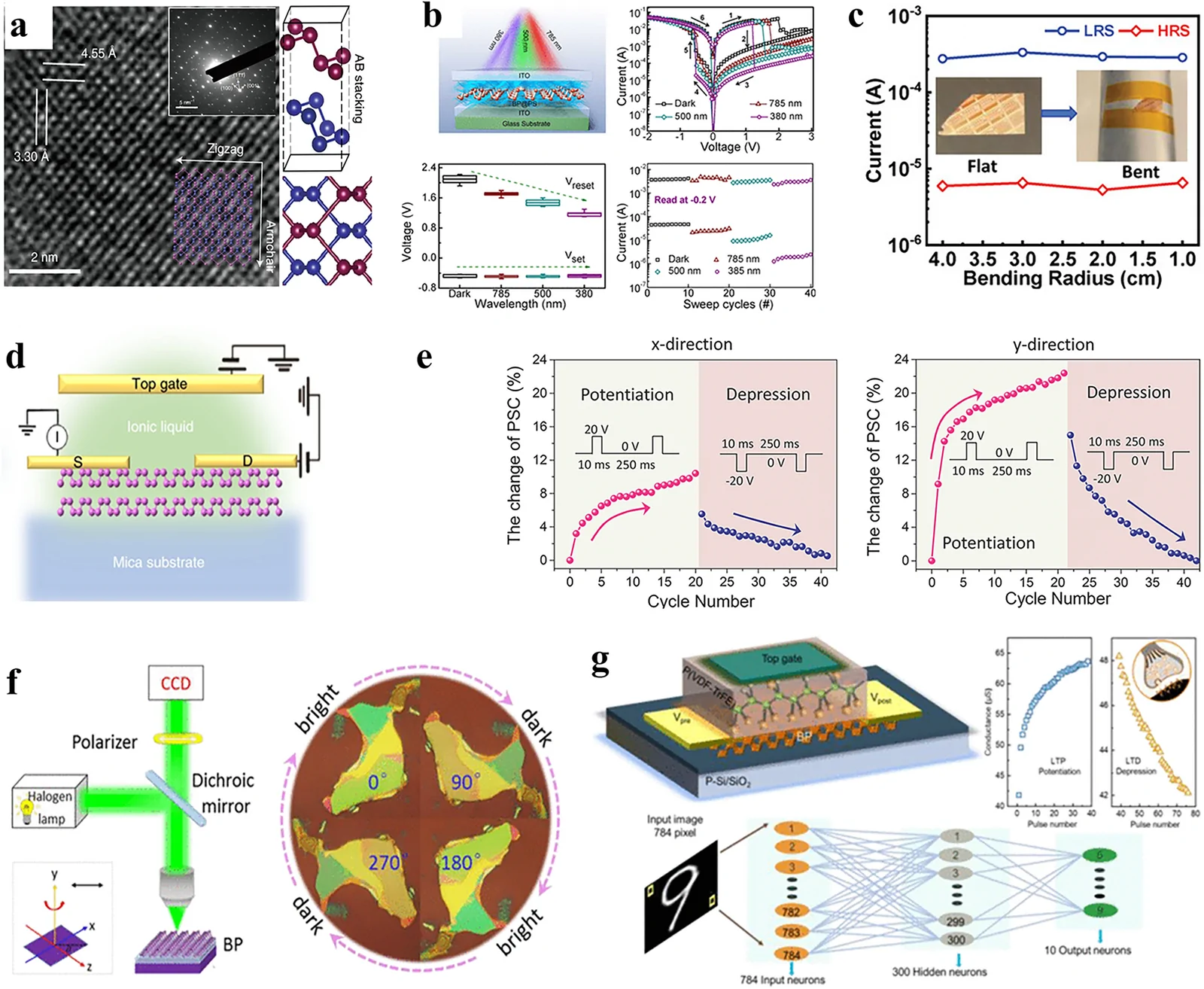
**a** Plan and cross-sectional HRTEM image of the large-scale-grown few-layer 2D BP. The plan view matches with the orthorhombic symmetry and cross-section layers showing the unique puckered structure. Reproduced with permission from Ref. [164]. **b** Light modulated memristive characteristics of BP. The BP as a switching material demonstrated the enhanced switching performance for the shorted wavelengths of light. Reproduced with permission from Ref. [162]. **c** Flexible optoelectronic synapse base on the BP for the visual perception application. It successfully demonstrated the PPF index variation and post-synaptic current response for the appropriate input light stimuli. Reproduced with permission from Ref. [165]. **d** Schematic image of the BP-based transistor. Reproduced with permission from Ref. [164]. **e** Anisotropic synaptic behaviour of the first fabricated BP-based transistor with distinct potentiation, depression and STDP for the x and y-direction of the device. Reproduced with permission from Ref. [163]. **f** Sensitive optical anisotropy of the BP-flakes in the visible region. Reproduced with permission from Ref. [166]. **g** Heterostructure of BP/(PCVDF-TrFe), with its transfer curves, synaptic function and synaptic illustration for the pattern recognition. Reproduced with permission from Ref. [167]

Figure 6 provides an overview of BP structures, highlighting their anisotropic properties and applications in neuromorphic memristors designed to emulate brain functions. Figure 6a [164] illustrates the defect-free atomic arrangement of an eight-layer BP film. The lattice parameters align well with an orthorhombic crystal structure and exhibit AB stacking. The cross-sectional high-resolution transmission electron microscopy (HRTEM) image clearly reveals multiple BP layers, each displaying the characteristic puckered configuration composed of double atomic layers. Additionally, a vertical stacking architecture for a transparent memristive device has been realized using an ITO/BPA@PS/ITO structure.

The BP nanosheets have been coated with polystyrene, which serves as a switching layer for the memristor. Figure 6b [162] demonstrates the switching characteristics of the device without externally irradiating light of wavelength 380, 500, and 785 nm. The resistance of the device in its high-resistance state was strongly dependent on the illumination wavelength. When exposed to higher-energy (shorter-wavelength) light, the reset voltage decreased due to enhanced photocarrier generation in the BP layer, indicating that the device’s switching characteristics are highly sensitive to optical excitation.

The BP-based bilayer heterojunction BP/HfO_x_ was employed to develop a flexible optoelectronic neuromorphic device, as illustrated in Fig. 6c [165]. This device exhibited stable synaptic functionality under bending conditions with a radius of curvature as small as 1 cm. When exposed to varying intensities of light pulses (dark, 32, 48, 57, and 68 mW cm^−2^), the device demonstrated a significant photoinduced synaptic effect (PSE), highlighting its potential for applications in flexible optoelectronic neurons.

These results underscore the promising capabilities of BP-based devices for optical sensing and synaptic functionalities in artificial vision systems and wearable technologies. Furthermore, BP serves effectively as a channel material for synaptic devices, as schematically depicted in Fig. 6d [164]. Figure 6e [163] presents the electrical and synaptic properties of the pioneering synaptic device that employs BP as the receptor and drain components within a neuromorphic transistor. The device successfully replicates anisotropic synaptic behaviours such as potentiation, depression, and STDP, demonstrating its capability to emulate the complex electrical anisotropy found in biological neural networks.

The in-plane optical anisotropy of BP in the visible region of the electromagnet spectrum has been demonstrated in Fig. 6f [166]. The optical anisotropy of BP is used to find the refractive indices in the visible region (480 to 650 nm), suggesting the possibility of designing BP-based novel optical devices. The optical anisotropy of BP flake was explored and further demonstrated the Hebbian learning rule, i.e., STDP, using these synaptic devices [168]. To investigate the optical anisotropy of BP, two perpendicular pairs of electrodes were fabricated along its principal crystallographic directions—namely the armchair (x-axis) and zigzag (y-axis)—allowing independent measurement of photocurrent responses along each axis. The optical neuromorphic device clearly showed the potentiation for 280 nm high and depression for the wavelength of 365 nm. The opposing polarity photon response of BP has three distinct applications, including integrated Boolean operations, optical initiation of synaptic functions and distinguishing between UV-A and UV-B radiations.

Figure 6g [167] illustrates a ferroelectric synaptic transistor fabricated by integrating 2D BP with a flexible ferroelectric copolymer, poly(vinylidene fluoride-trifluoroethylene) (P(VDF-TrFE)). The synaptic transistor has excellent mobility of 900 cm^2^ v^−1^ s^−1^ with an on/off current ratio of 10^3^ and operation energy of around 40 fJ. The high mobility of the 2D BP channel was believed to contribute to the signal transmission within the artificial synapse and hence reduced the power consumption, whereas the conductance change was favourable for in-memory computing. Compared with TMDs and h-BN, BP offers higher carrier mobility and strong anisotropic transport, making it especially attractive for direction-sensitive neuromorphic devices and optoelectronic synapses. However, its poor ambient stability and rapid oxidation remain major obstacles for practical deployment. Advanced encapsulation and passivation strategies are therefore necessary for large-scale integration

### Tellurene and Other Novel Materials: Emerging Tellurium-Based and Other Newly Discovered 2D Materials Relevant for Neuromorphic Computing

While h-BN, BP, and 2D TMPs have emerged as promising materials exhibiting excellent switching behaviour, the exploration of alternative systems remains crucial for advancing semiconductor technology. Inspired by the remarkable anisotropic properties of BP, mono-elemental 2D materials (Xenes), such as tellurene, arsenene, and silicene have been studied in depth [169, 170]. Unlike other established 2D materials, the electronic properties of Xenes strongly depend on their elemental composition and thickness. By carefully adjusting thickness from bulk to nanoscale, insulating, semiconducting, or metallic properties can be precisely tuned [170].

In particular, Xenes offer layer-dependent bandgap tuning [171], a key requirement for developing neuromorphic devices that imitate synaptic functions [172–175]. Another significant advantage of Xene materials is their ability to introduce and control defects, which enhances their performance as memristive devices in neuromorphic applications. Additionally, Xenes demonstrate strong optical absorption spanning ultraviolet (UV) to near-infrared (NIR) wavelengths, making them ideal candidates for optoelectronic applications. Consequently, Xenes have become an exciting class of materials exhibiting memristive and synaptic behaviour.

Tellurene films, specifically, are considered promising due to their moderate bandgap, air stability, and nearly symmetrical carrier mobility for electrons and holes, making them suitable for fabricating memristors and transistors. Qiu et al. [176] successfully fabricated an n-FET based on atomic-layer-deposited tellurene films and observed nearly symmetrical operational characteristics compared to tellurene-based p-FETs. Grazianetti et al. [177] demonstrated the integration of carefully engineered silicene into field-effect transistors. To protect silicene from air exposure, they employed an Al_2_O_3_ capping layer, ensuring stability and without affecting device performance.

The bottom-gate FET with silicene as channel material demonstrated charge transport properties comparable to those of graphene. High-quality solution-processed 2D tellurium (tellurene) crystals with precisely tunable thicknesses, ranging from monolayers to tens of nanometers, have also been developed. Tellurene’s van der Waals layered structure exhibited strong in-plane anisotropy typical of other 2D materials. Devices fabricated with tellurene showed remarkable air stability for up to two months, an ON/OFF ratio of 10^6^, and carrier mobility reaching approximately 700 cm^2^V^−1^ s^−1^, positioning them as promising candidates for synaptic transistor applications.

Guo et al. [178] employed plasma-assisted defect engineering to introduce controlled vacancies in tellurene nanosheets, enabling modulation of carrier concentration and photoresponse. This tunable defect density allowed the device to emulate optical synaptic behaviour, where the synaptic weight could be dynamically adjusted through light intensity and electrical stimuli. The memristive and neuromorphic switching characteristics in nanoscale 2D bismuth selenide (BiSe) crystals also show that the switching behaviour was governed by Bi-ion concentrations in Bi-rich regions. Compliance current-controlled multilevel switching indicated the potential for thin BiSe devices to act as artificial neurons. Li et al. [179] explored advanced channel materials, extending the device scale to approximately 3 nm, consistent with Moore’s Law.

Organic 2D materials have also emerged as promising active layers for memristors and memtransistors, exhibiting neuromorphic behaviour [133, 180]. Wang et al. reported an optical synaptic device employing 2D pentacene within the heterostructure Au/pentacene/PMMA/CsPbOBr_3_-QDs/SiO_2_/Si, demonstrating excellent excitatory post-synaptic currents (EPSC) under optical stimulation [181]. Liu et al. [182] fabricated highly reproducible and reliable memristors using ultrathin films of a 2D imine polymer (2DP). Their Ag/(2DPBTA + PDA)/ITO device exhibited exceptional flexibility, with stable performance after up to 500 bending cycles and thermal stability in the range of 50 to 400 °C. The resistive switching behaviour demonstrated a film-thickness-dependent ON/OFF ratio ranging from 10^2^ to 10^5^, with data retention up to 8 × 10^4^ s.

**Fig. 7:**
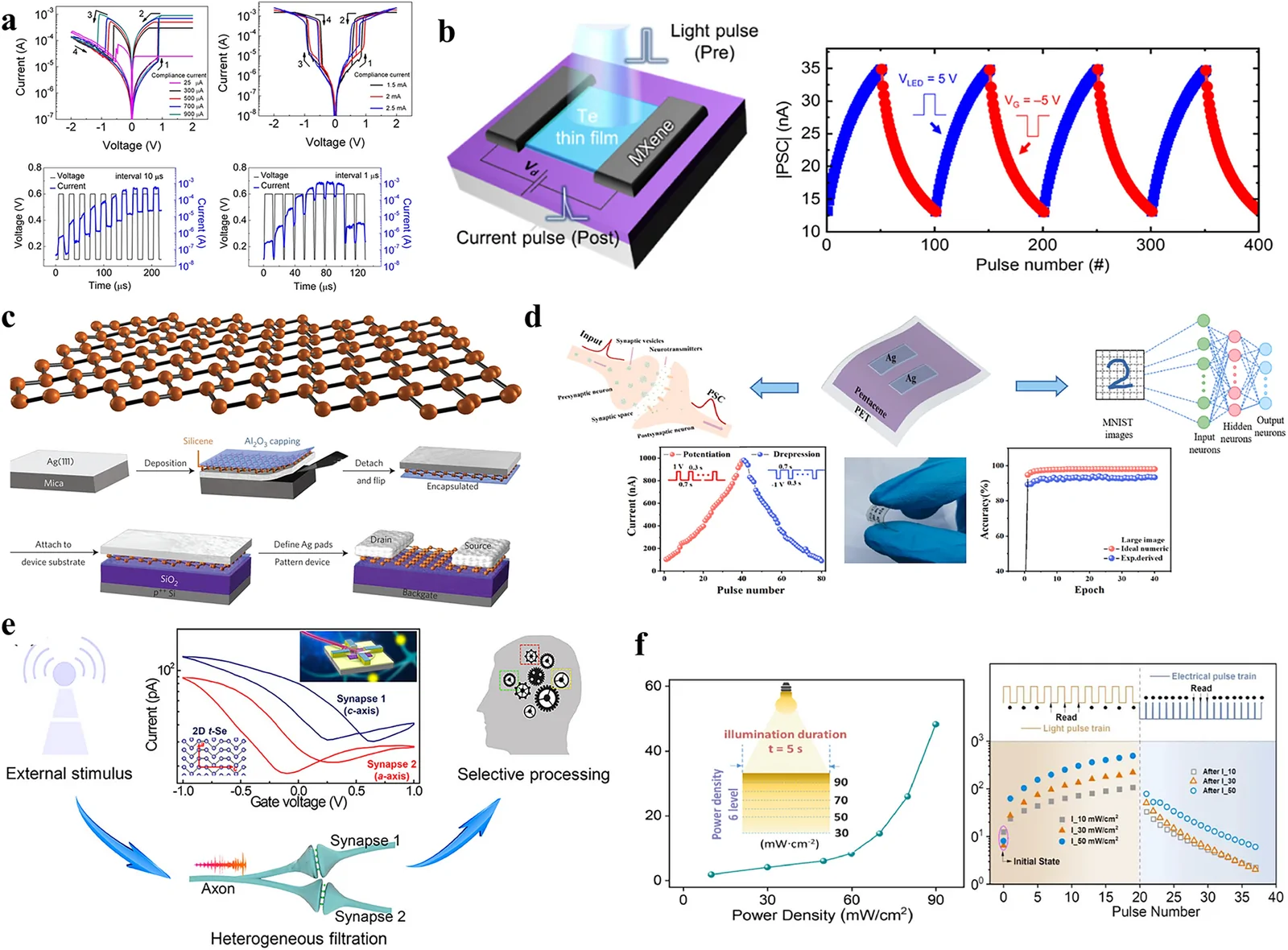
Performance of memristive and neuromorphic devices fabricated by tellurene and other emerging 2D materials. **a** All Te-based memristor showing the unprecedented non-volatile and volatile switching for higher and lower compliance currents and synaptic response for an appropriate input voltage pulse. Reproduced with permission from Ref. [183]. **b** Te-based optical synaptic device for the reservoir computing and pattern recognition. The light pulses were used to emulate the synaptic functions. Reproduced with permission from Ref. [184]. **c** Silicene (2D-Si)-based transistor fabricated by synthesis-transfer-fabrication process Reproduced with permission from Ref. [185]. **d** Trigonal selenium nanosheet channel transistor with the unique electrical anisotropy. The anisotropic weight modulation well suited for the temporal filtering ability. Reproduced with permission from Ref. [186]. **e** Flexible Ag/pentacene/SiO_2_/Si structure device demonstrating the digital and analogue switching for the higher and lower voltage sweeps respectively. The device in its analogue switching regime demonstrated the PPF index, potentiation and depression for the appropriate voltage input stimuli. Reproduced with permission from Ref. [187]. **f** TIPS-pentacene-based optical phototransistor array retina to mimic bionic eye, which demonstrated unique potentiation for the different intensities of light irradiated on it. Reproduced with permission from Ref. [188]

Figure 7 summarises the excellent contribution of tellurene and other emerging 2D materials as switching layer/channel materials in memristors and memtransistors for neuromorphic applications. Yang et al. [183] developed all Te-based crossbar array memristors, as shown in Fig. 7a. The device exhibited unique switching behaviour with volatile switching for the low operating current (compliance current) and non-volatile memory for the higher compliance current. The current-controlled Te filament formation was the fundamental reason for the non-volatile electrochemical switching and volatile switching caused by the disruption of the Te filament by joule heating. By controlling the interval between the pulse trains of width 10 µs and height ± 0.6 V, the device demonstrated a transition from long-term to short-term plasticity and vice versa and low-pass filter application. In another report, Te film was used as active layer of the artificial photonic synapse base reservoir computing by Jo et al., as shown in Fig. 7b [184]. This back-gated photonic transistor with Te as channel materials consisting of MXenes electrode was employed as a photonic synapse. Light pulses were used as pre-synaptic stimuli, producing a transient increase in photocurrent analogous to the excitatory post-synaptic current (EPSC) observed in biological neurons. This analogy reflects the device’s ability to emulate neural excitation, where optical inputs strengthen the synaptic conductance in an artificial synapse. Hence, Te is an emerging material for the synaptic device that operates from an electrical/photonic input.

The 2D silicon called silicene, an analogue of graphene, has emerged while seeking more efficient 2D materials for application in intelligent electronic devices. The sensitive surface of silicene and its Dirac band structure offers potential applications to influence the future of intelligent electronic devices like synaptic memristors and memtransistors. Tao et al. [185] reported a silicene-based transistor (Fig. 7c) fabricated using the synthesis-transfer-fabrication process, in which the silicene employed a channel and Ag as a source and drain. The I–V curves with zero gate voltage showed nearly linear I_d_−V_d_ for very low voltages, and I_d_−V_g_ transfer curve characteristics displayed the ambipolar hole-electron symmetry. While searching for the better choice in 2D materials, Qin et al. [186] implemented trigonal Selenium (t-Se) as a channel for their fabricated synaptic transistor, which presents the excellent anisotropic response of filtering behaviour to the same stimulus and hence potential synaptic device as shown in Fig. 7d. The low conductivity of the t-Se channel caused an extremely low energy consumption of 10 pJ with strong anisotropy along the different directions/axes of the film. By the intrinsic homogeneity of electrical conductivity, the t-Se nanosheet transistor demonstrated prominent anisotropy in the synaptic weight modulation and temporal filtering ability.

Organic 2D materials and their thin films have unique properties like biocompatibility. Han et al. [187] reported a flexible organic pentacene thin film-based memristor for neuromorphic computing. The Ag/pentacene/SiO_2_/Si structured memristor demonstrated digital and analogue switching for applying higher and lower voltage sweeps. The SET and RESET voltages for bipolar switching was 4.10 and 4.50 V, respectively, whereas the gradual increase and decrease in the current were observed for a successive voltage sweep of ± 0.5 V, as shown in Fig. 7e. In the low voltage region, the device demonstrated synaptic functions like potentiation, depression and PPF index when stimulated by voltage pulses of a height ± I.0 V, 0.3 s width and 0.7 s interval. In another effort, Zhang et al. [188] analysed the bionic eye of the TIPS-pentacene channel-based phototransistor array retina, as shown in Fig. 7f. They reported that the device effectively mimics human retinal function by exhibiting strong broadband photosensitivity (covering 380–740 nm), low power consumption (~ 3 nW optical, ~ 400 pW electrical) and biocompatible integration enabled by its organic semiconductor composition, allowing safe interfacing with biological tissues for potential bio-neuromorphic and retinal prosthetic applications. It replicates synaptic behaviours such as paired-pulse facilitation, spike-rate-dependent plasticity, and light-triggered memory processes, enabling visual sensing, memory, and in-sensor processing in a flexible, fully organic, dual-gated transistor structure. Hou et al. [189] proposed two-terminal optical synapses with pyrenyl graphdiyne/graphene/PbS quantum dot heterostructure exhibiting extraordinary flexibility for wearable electronics. The excitatory post-synaptic current and inhibitory post-synaptic currents were triggered in this artificial optical synapse by irradiation of light with wavelengths of 450 and 980 nm. The device maintained its maximum and minimum conductance (*G*_max_ and *G*_min_) for 1000 bending cycles. Hence, the organic material heterostructured device can act as optical synapse for wearable neuromorphic computing. Although tellurene, silicene, trigonal selenium, and other emerging 2D materials exhibit promising neuromorphic characteristics, most remain at an early proof-of-concept stage. Compared with more established materials such as MoS_2_ and h-BN, these systems still face greater challenges in synthesis reproducibility, environmental stability, and wafer-scale integration. Nevertheless, their unique transport and optoelectronic properties make them attractive for future multifunctional neuromorphic systems.

## Fabrication Techniques and Device Configurations

### Growth Methods: Chemical Vapour Depositions, Molecular Beam Epitaxy, and Solution-Based Synthesis Techniques Used from 2D Materials

**Fig. 8:**
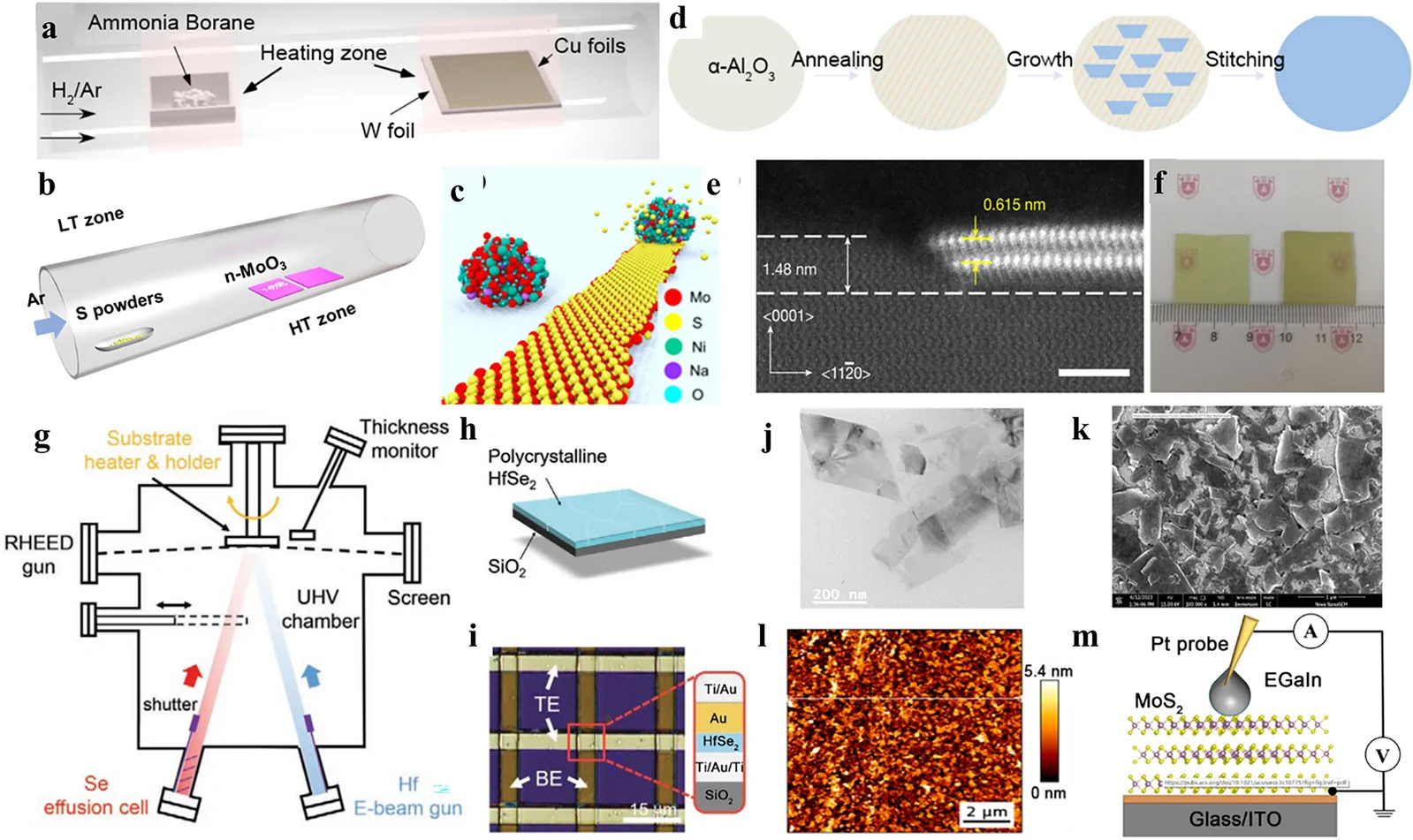
**a** Schematic illustration of experimental setup of CVD growth of h-BN [193]. **b** Illustration of growth method for TMDs. LT stands for low temperature. HT stands for high temperature. **c** Schematic diagram of growth of TMDs nanoribbons through VLS mechanisms [194]. **d** Illustration of the growth process of a TMDs film by atomic step edge induced orientation aligned growth and coalescence of domains growth [195–198]. **e** Transmission electron microscope (TEM) picture of the bilayer MoS_2_ grown from the step edge. **f** Picture of 1 × 1 cm^2^ continuous bilayer MoS_2_ film [199]. **g** Depiction of the setup of MBE to grow wafer-scale polycrystalline HfSe_2_ thin film **h** on SiO_2_. **i** HfSe_2_ film was employed to fabricate the memristor crossbar array with Ti and Au electrodes. **j** TEM picture of liquid exfoliated 2D MoS_2_ nanosheets [200]. **k** Scanning electron microscope (SEM) image of 2D MoS_2_ thin film deposited on a glass substrate. **l** AFM topographic images of the continuous 2D MoS_2_ thin film assembled by LPE MoS_2_ nanosheets [201]. **m** Schematic representation of ITO/MoS_2_/EGaIn memristor device [202]. Reproduced with permission. Copyright 2022, IOP Publishing. Copyright 2022, Springer Nature. Copyright 2021, Wiley. Copyright 2024, American Chemical Society

CVD has become a cornerstone technique in the synthesis of 2D materials, offering a robust and scalable method for producing high-quality, atomically thin layers. This process involves the chemical reaction of gaseous precursor molecules on a heated substrate, where the precursors decompose or react to form the desired 2D material. For instance, in the synthesis of -BN [190], ammonia borane (BH_6_N) or borazine (B_3_H_6_N) are commonly used as boron and nitrogen source, as depicted in Fig. 8a, while Cu (111) is used as substrate, promoting the growth of crystalline h-BN [191]. The CVD setup typically includes a furnace where the substrate, metals, or insulator substrates such as sapphire and mica are heated to a high temperature [192]. Here, the choice of substrate, temperature, pressure, gas flow rates, and the composition of the gas mixture are meticulously controlled to influence the nucleation, growth rate, and quality of the 2D material [193].

For transition metal dichalcogenides (TMDs) like MoS_2_ or WS_2_, the process involves precursors such as molybdenum oxide (MoO_3_) or tungsten oxide (WO_3_) for the transition metal and sulphur or selenium for the chalcogen [192, 203, 204]. These precursors vapourize at elevated temperatures, typically between 500 and 1000 °C [204, 205], to form the desired TMDs in the vapour–solid–solid (VSS) growth mode [206] as shown in Fig. 8b, c, sulphur and transition metal oxide vapour are transported onto the solid growth substrates to initiate the chemical reaction, which prompts the nucleation and growth of solid 2D TMDs. The growth can occur through different mechanisms, such as vapour–liquid–solid (VLS) [194] processes, where nanostructures are produced by precipitation from ambulant supersaturated catalytic liquid droplets. Furthermore, Fig. 8d shows that atomic step edges have been found to destroy the symmetry of the substrate and align the flake orientation in the same direction toward seamless coalescence for large area growth [195–198]. As depicted in Fig. 8e, f, Liu et al. used the C/A 1° miscut substrate with 1350 ℃ annealing to grow out 1 cm^2^ continuous bilayer MoS_2_ film. Moreover, low-temperature growth compatible with the back-end-of-line (BEOL) is under considerable attention. Recently, Zhang et al. developed a universal low-temperature method to grow two-dimensional metal chalcogenides below 400 °C. This method uses the low-barrier-energy iodine–chalcogen exchange and is compatible with standard CMOS techniques [199]. Other 2D materials such as 2D metal oxides are also promising in neuromorphic related devices. Hong et al. used the reversed reaction of MoS_2_ and oxygen, combined with charges from plasma pretreatment of substrates to grow out slantly standing ultrathin 2D α-MoO_3_ for memristor fabrication. The lower thickness reduced the set voltage effectively [207]. Seems underwhelming. Why discuss it if the benefits are so marginal?

One of the key advantages of CVD is its scalability, enabling the production of large-area, uniform films that are crucial for practical applications in electronics and optoelectronics, among others [208]. Moreover, CVD allows for the incorporation of dopants or the creation of heterostructures by sequentially introducing different precursors, thus tailoring the electronic band structure or introducing new functionalities [209]. Challenges include obtaining wafer-scale growth [210], control over the layer thickness [204], growth in the low enough temperature, and achieving high-quality, defect-free growth. In ML, particularly neural networks, memristors can act as synaptic weights, enabling in-memory computing. This reduces the energy-intensive data shuffling between memory and processing units in traditional architectures. Memristor or memtransistor-based crossbar arrays perform matrix–vector multiplications in hardware, accelerating neural network operations. All these hardwares call for large area materials, whose foundation relies on the wafer-scale growth. Moreover, the layer thickness can affect device performance. For example, different thickness layers possess various passage length for the conductive filament formation in the memristors. To enable integration into industrial CMOS fabrication, the growth temperature must be BEOL-compatible. Last but not least, the quality of the materials directly influences the neuromorphic device performances, including variability, endurance, noise, etc. However, certain micro- and nanostructures can improve device performance. For instance, the defects can sometimes function as beneficial factors for charge trapping/detrapping in neuromorphic devices [211]. Grain boundaries were recently found to be beneficial passages for conductive filament-based memristors [212]. Researchers have thus focused on optimizing growth conditions, exploring new precursors, and developing in situ characterization techniques to monitor and control the growth process in real-time [204, 213]. Besides enhancing our understanding of 2D material growth dynamics, these studies also push the boundaries of what these materials can achieve in terms of performance and application, making CVD an indispensable tool in the field of 2D materials [45, 47, 49–52, 54, 214, 215].

Molecular beam epitaxy (MBE) has also proven to be effective for the growth of 2D materials. Conducted in ultra-high vacuum (10^–8^–10^–12^ Torr), MBE employs a slow deposition rate to ensure the epitaxial growth of highly uniform and crystalline films. This method benefits from the ultra-pure environment, enhancing film quality. Real-time monitoring of crystal layer growth can be followed using techniques like reflection high-energy electron diffraction (RHEED). However, the slow growth rate (< 3000 nm h^−1^) and high setup costs are notable drawbacks. MBE has been employed to deposit high-quality 2D materials. Recently, as shown in Fig. 8g–i, Li et al. used an ultra-high vacuum MBE system to deposit wafer-scale polycrystalline 2D HfSe_2_ to make a memristor crossbar array (CBA) to perform hardware convolution image processing [200]. MBE, with stable and well-controlled beam flux and the rotational substrate with uniform temperature distribution, enables 2-inch size material growth, effectively eliminating the occurrence of by-products and impurities.

Solution-based synthesis techniques offer a versatile and cost-effective alternative to vacuum-based technologies. These methods typically involve the chemical reaction of precursors in a liquid medium or intercalation, often at lower temperatures than that required for CVD [216]. Techniques such as liquid-phase exfoliation, hydrothermal synthesis and solvothermal synthesis and are commonly used. Liquid-phase exfoliation (LPE) involves the mechanical or chemical exfoliation of bulk layered materials in a solvent, producing few-layer or single-layer 2D materials. Saha et al. used LPE to prepare a uniform dispersion of few-layer-thick 2D MoS_2_ nanosheets in N-methyl-2-pyrrolidone (NMP) and later transferred to isopropyl alcohol [201]. A thin film was then grown using a biphasic method, which involved scooping a thin film at the liquid–liquid interface of two immiscible liquids (Fig. 8j–l). As shown in Fig. 8m, a thin film of LPE 2D MoS_2_ was pinched between two electrodes to fabricate memristors and demonstrated edge computation and adaptive learning behaviour. In hydrothermal and solvothermal processes, on the other hand, precursors react in a sealed vessel under high pressure and temperature, allowing for the growth of 2D materials like TMDs or metal oxides (MOs) in solution [202]. These approaches are particularly advantageous for ease of doping or functionalization, the ability to produce materials in macroscopic quantities, although they might not always yield the same level of crystallinity or purity compared to vapour-phase methods.

To conclude, different synthesis techniques offer distinct advantages and limitations for neuromorphic applications. CVD is highly attractive for large-area growth, although defect formation and non-uniform thickness remain major concerns. MBE provides excellent crystal quality and interface control but is expensive and difficult to scale. Solution-based methods offer low-cost and flexible processing, but reproducibility and thickness control remain limited. At present, no single fabrication method can simultaneously satisfy the requirements for wafer-scale uniformity, low defect density, CMOS compatibility, and low-temperature processing.

Table 2 summarizes and compares the strengths and limitations of the above-mentioned growth methods, with some typical materials and corresponding applications. Each method has advantages and limitations and should be chosen on the basis of the desired target materials and experimental conditions.

**Table 2:** Comparison of the above-mentioned growth methods with typical materials and applications

Growth method	Advantages	Disadvantages	Typical Materials	Applications	References
CVD	Versatility: Suitable for a wide range of 2D materials. Cost-effective: Relatively low setup cost compared to other methods. Handling easiness: Generally easy to handle. Scalability: Can produce large-area films. Integration: Compatible with existing semiconductor processes	Growth Rate: Can be slow, especially for high-quality films. Defects: Generation of defects like grain boundaries and impurities. Complexity: Many influential factors like temperature, pressure, and gas flow and precise control is hard. Scalability Challenges: Uniformity over large areas and thickness control can be challenging. The growth temperature is usually high	Graphene	Memristive synapse	[217]
			MoS_2_	Memristors for neuromorphic computing	[218]
			WSe_2_	Artificial synapse	[219]
			ReS_2_	Memristors for neuromorphic computing	[220]
			SnS_2_	Ferroelectric analogue synaptic device	[221]
			PtSe_2_	Bidirectional photoresponse and artificial optoelectronic synapses	[222]
			MoTe_2_	Memristor array for artificial synapses	[223]
			MoO_3_	Memristors	
MBE	High Purity: Produces highly pure, defect-free films. Precision: Offers atomic layer control over growth. Interface quality: Atomically sharp interfaces Versatility: Can grow complex heterostructures. In-situ monitoring: Allows real-time monitoring of growth	Cost: High setup and operational costs due to ultrahigh vacuum requirements. Slow growth: Growth rates are typically very slow. Scalability: Not easily scalable to large areas. Complexity: Requires highly skilled operators and precise control. Limited materials: Not all materials can be grown due to high-temperature requirements	Mo-ReS_2_	Non-volatile switching, programmable multilevel resistance states, long-term synaptic plasticity	[224]
			HfSe_2_	Memristor crossbar array, hardware multiply-and accumulate (MAC) operation, hard ware convolution image processing	[200]
			VO_2_	Sensory neuron, spike-based neuromorphic multisensory system	[225]
			In_2_Se_3_	Ferroresistive memory Junction, giant Electroresistance Switching	[226]
Solution based synthesis	Scalability: Can be highly scalable for solution processable materials. Low cost: Generally lower setup and operational costs. Flexibility: Suitable for flexible electronics and solution-processed devices. Tunability: Easy to modify material properties through solution chemistry. Ambient conditions: Often does not require ultrahigh vacuum or extreme temperatures	Thickness control: Limited control over layer thickness. Uniformity: Can struggle with achieving uniform film thickness over large areas. Oxidation: Materials can be prone to oxidation or degradation in solution. Contamination: Potential for impurities from solvents or precursors. Limited materials: Not all 2D materials are suitable for liquid synthesis	MoS_2_ nanosheets	Memristors, edge computation and adaptive learning	[201]
			MXene	Reservoir computing networks	[227]
			2D Metal–organic framework	Optoelectronic neuromorphic Transistors, human Emotion Simulation	[228]
			Black phosphorus	Optoelectronic memristive synapses, artificial visual perception	[165]

### Device Architectures: Memristors, Memtransistors, and Vertical Heterostructures in Neuromorphic Circuits

#### Memristors

**Fig. 9:**
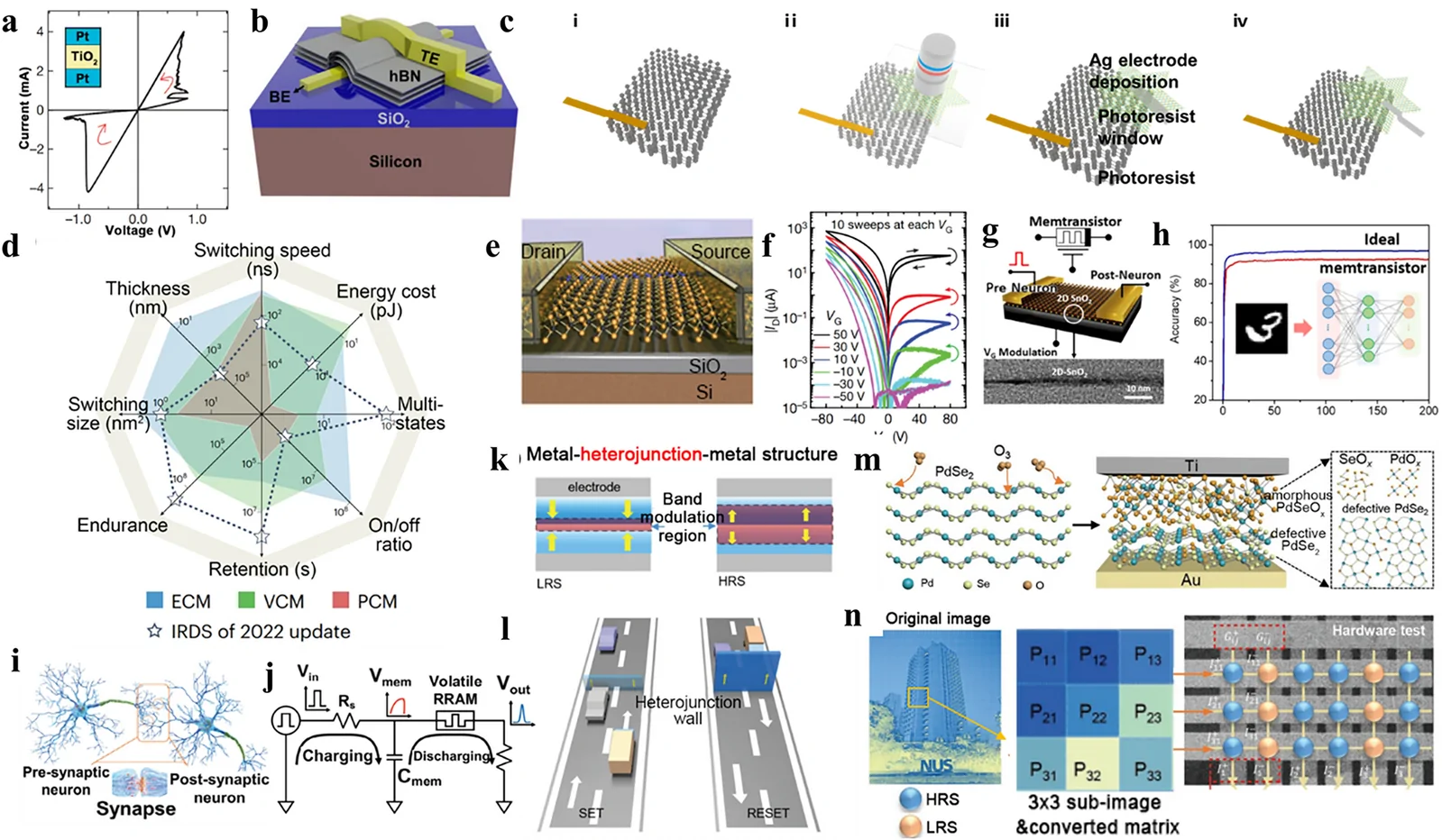
**a** Experimental I–V plot of a Pt-TiO_2_-x-Pt device [230]. **b** Schematic drawing of the memristor based on h-BN. BE: bottom electrode. TE: top electrode [231]. **c** Depiction of the device fabrication process with the alignment on specific position GBs [212]. **d** Evolution of the critical benchmarks for three types of switching mechanism, including ECM, VCM and PCM. Open stars indicate the IRDS 2022 requirements [207]. **e** Schematic of a MoS_2_ memtransistor device built on 300-nm-thick thermal SiO_2_ on doped Si (gate) [124, 232]. **f** ID–VD curves for ten consecutive sweeps at each gate bias VG for the MoS_2_ memtransistor. The switching directions are indicated by the curved arrows. **g** Illustration of the inverted FET structure memtransistor using the 2D-SnO_2_ memristor channel and cross-sectional TEM images of the 2D-SnO_2_ nanosheet [232]. **h** Schematic illustrations of three-layer neural network for pattern recognition simulation and simulated pattern recognition accuracy of the 2D-SnO_2_ memristor with gate bias [232]. **i** Schematic depiction of a biological neuron [212]. **j** Circuit diagram for mimicking neuron behaviour [212]. **k** Change of resistance of the memristor based on WS_2_/MoS_2_ heterostructure is caused by the band modulation of the heterogeneous junction. It is similar to a “lift gate” on the expressway **l** [233]. **m** Schematic image showing single-crystalline PdSe_2_ nanosheet treated by UV-ozone. The pristine PdSe_2_ nanosheet is converted into heterostructure with an amorphous PdSeO_x_ overlying the defective PdSe_2_ [234]. **n** Convolutional image processing implemented using PdSeO_x_/PdSe_2_ memristor crossbar array [235]. Reproduced with permission. Copyright 2008, 2018, 2023, Springer Nature. Copyright 2021, 2024 American Chemical Society

As briefly described earlier, memristor (short for memory resistor; Fig. 9a) is a fundamental electronic component whose resistance depends on the history of the current that has passed through it, thereby providing a memory function analogous to the operation of biological synapses. First theorized by Leon Chua in 1971 and physically realized by HP Labs in 2008 [229], memristors have since been widely studied for applications in non-volatile memory, neuromorphic computing, and logic circuits. Their analogue resistance states allow efficient, low-power in-memory processing and parallel matrix operations, addressing the von Neumann bottleneck in conventional architectures.

##### Basic Principles and Operation

2D-material-based memristors operate through ionic or electronic transport within the active layer, which modulates the device’s resistance under an applied electric field. Depending on the material system, resistive switching can arise from vacancy migration, charge trapping/detrapping, or interface modulation. Representative examples include TMDs, h-BN, and 2D metal oxides (Fig. 9b), each exhibiting distinct switching dynamics governed by their atomic thickness and surface defect chemistry [212, 236]^.^

##### Recent Progress

(i) *Integration with 2D Materials*: Recent advancements include the successful integration of 2D materials like MoS_2_[212], WSe_2_[237], h-BN[231], and graphene into memristor structures. These materials enable the creation of ultrathin, flexible, and transparent memristive devices, expanding their potential applications in wearable electronics and flexible displays.
(ii) *Enhanced Performance*: Research has focused on improving the switching speed, endurance, and retention time of 2D-material-based memristors. For instance, studies have shown that vertical stacking of 2D materials can lead to multilevel resistance states, enhancing memory density and enabling analogue computing capabilities [238].
(iii) *Material Exploration*: Material engineering, especially defect engineering, can be used to improve the performance of TMDs-based memristors. Recently, Lan et al. synthesized position-controlled, star-shaped MoS_2_ flakes with well-defined grain boundaries (GBs). During device fabrication, these flakes were precisely aligned onto the desired GB regions using a dry-transfer process, enabling controlled ion migration pathways and significantly reducing the set voltage—by nearly 16 folds (Fig. 9c) [212]. Besides, other 2D materials beyond TMDs like BP [165], MXenes [239], and 2D Mos [207] are being explored for their memristive properties. These materials offer different mechanisms for resistance switching, such as electrochemical memristor (ECM), valence change memristor (VCM), or phase-change memristor (PCM), providing a rich palette for device design with different device properties (Fig. 9d).
(iv) *Neuromorphic Computing*: 2D materials-based memristors have shown promise in neuromorphic computing, as demonstrated in Fig. 9i, j, where they can mimic synaptic functions under spiking signal input. Recent work has demonstrated the use of these memristors in neural networks. For example, the memristors are built into a network array for handwritten digit pattern memory and recognition [212], showcasing their ability to perform in-memory computing, reducing energy consumption and latency.
(v) *Scalability and Fabrication*: Advances in fabrication techniques like CVD and ALD have made it possible to produce large-area, uniform 2D material films, crucial for scaling up memristor arrays. For instance, Mattinen et al. demonstrated the deposition of polycrystalline, wafer-scale MoS_2_, TiS_2_, and WS_2_ films of controlled thickness at record-low temperatures down to 100 ℃ using plasma-enhanced ALD. They observed that high H_2_ flow ratio can enhance the crystallinity [240]. These progress aids in the practical realization of high-density memory devices.
(vi) *Quantum and topological Memristors*: The exploration of quantum and topological effects in 2D materials-based memristors is promising for quantum memory and computing applications. The use of quantum tunnelling and other topological phenomena to enhance or modify memristive behaviour is currently under investigation [241, 242].

#### Memtransistors

Memtransistors, a combination of memristor and transistor, are emerging devices that combine the memory functionality of a memristor with the switching capabilities of a transistor, offering multiterminal control and enhanced tunability. They can emulate complex synaptic behaviours, such as STDP, which is key for neuromorphic systems mimicking brain-like learning. Memtransistors provide greater control over synaptic weights, improving precision in learning algorithms. When fabricated using 2D materials, these devices offer distinctive advantages due to the atomically thin nature of the materials, enabling high-density integration, low power consumption, and potential for flexible electronics.

##### Basic Principles and Operation

A 2D materials-based memtransistor typically features a horizontal structure where a 2D material serves as channel between two electrodes with a third electrode as a gate, similar to the field-effect transistor, as shown in Fig. 9e [124, 232]. Figure 9f shows that the device can operate by controlling both charge transport and memory effect. The gate electrode modulates the channel’s conductivity, while the memory effect arises from the modulation of the channel’s resistance or conductance based on the history of applied voltages or currents. This dual functionality allows for in-memory computing, where data processing and storage occur in the same device, reducing latency and power consumption.

##### Recent Progress

(I) *Material Exploration* Recent studies have featured various 2D materials for memtransistor applications, such as TMDs like MoS_2_, WSe_2_, and ReS_2_. As shown in Fig. 9g, recently, Huang et al. used liquid–metal printing technique to synthesize 2D-SnO_2_ to fabricate memtransistors. By varying the gate voltage, the recognition accuracy of handwritten digits was improved to 92.25% (Fig. 9h) [232]. This memtransistor also offers low-power consumption operation and showed high potential to be developed as an energy-efficient biological neural systems such as the human brain (~ fJ), putting forward metal oxides as strong candidates for memristors with excellent resistive switching characteristics [232]. All these materials offer tunable electronic properties, allowing for the design of memtransistors with specific memory and switching characteristics.
(II) *Enhanced Memory and Switching* Advances have been reported in improving the memory retention time, switching speed, and endurance of 2D materials-based memtransistors. For instance, researchers have demonstrated multilevel memory states in 2D MoS_2_-based memtransistors, enabling analogue computing capabilities. The MoS_2_ memtransistors show gate tunability in individual resistance states by four orders of magnitude, as well as large switching ratios (higher than 100), high cycling endurance (475 times) and long-term retention of states (24 h and can be further projected into one year) [124]. These devices can store multiple resistance states, facilitating complex neural network operations.
(III) *Neuromorphic Computing* 2D materials-based memtransistors are particularly promising for neuromorphic computing, where they can emulate the behaviour of biological synapses and neurons. Recent studies have shown that these devices can perform synaptic plasticity, STDP, and other neuromorphic functions, paving the way for brain-inspired computing systems. For instance, Huang et al. employed 2D-SnO_2_ via a liquid–metal route to fabricate memtransistor to examine the synaptic behaviour and achieved improved digit recognition accuracy up to 92.25% simply by gating [232].
(IV) *Flexible Electronics* The inherent flexibility of 2D materials makes them ideal for use in flexible electronics. Recent progress includes the demonstration of flexible, transparent, and wearable memtransistor devices, opening up applications in wearable technology, flexible displays, and electronic skin. Feng et al. used MoS_2_ ink to develop a scalable and low-temperature printing technique to realize a CBA structure based on aerosol-jet printed Ag/MoS_2_/Ag memristors. The fully printed devices exhibit an ultra-low switching voltage (0.18 V), a high switching ratio (10^7^), tunable resistance states for multibit data storage, and a low standby power consumption of 1 fW and a switching energy of 4.5 fJ per transition set, demonstrating the potential to enable energy-efficient artificial neuromorphic computing [243].
(V) *Integration with Existing Technologies* Efforts are underway to integrate 2D materials-based memtransistors with CMOS technology, aiming to leverage the benefits of both worlds. This hybrid approach seeks to combine the non-volatility, low power consumption, and in-memory computing capabilities of memtransistors using the established manufacturing processes of silicon-based electronics.

Although two-terminal memristors and three-terminal memtransistors are often discussed together as neuromorphic devices, their operating mechanisms and system-level advantages differ significantly. Two-terminal memristors are generally attractive for high-density crossbar arrays because of their simple structure, small footprint, and low operating energy. These devices are particularly suitable for vector–matrix multiplication and in-memory computing applications. However, their weight-update behaviour is often affected by nonlinear conductance changes, cycle-to-cycle variability, and sneak-path currents in large arrays, which can reduce training accuracy and complicate precise synaptic tuning. In contrast, three-terminal memtransistors provide an additional gate terminal that enables improved control over channel conductance and synaptic weight updates. This extra degree of freedom allows more linear and gradual conductance modulation, better separation between read and write operations, and reduced risk of unintended state disturbance during training. As a result, memtransistors are often more suitable for applications requiring precise analogue weight control, stable multilevel states, and on-chip learning, although their larger footprint may limit array density compared with two-terminal devices.

#### Vertical Heterostructures in Neuromorphic Devices

Vertical heterostructures, where different 2D materials are stacked layer by layer, have emerged as a promising approach for neuromorphic circuits [79, 244]. These structures influence the distinctive electronic, optical, and mechanical properties of 2D materials to mimic the functionality of biological neural networks, offering potential for energy-efficient, high-density, and versatile computing systems.

##### Basic Principles and Operation

In vertical heterostructures for neuromorphic circuits, 2D materials like graphene, TMDs, and h-BN are vertically stacked to form complex architectures [245]. Each layer can serve different roles, such as acting as a synaptic connection, a neuron, or a gate for controlling charge transport, tunnelling, or ion migration between layers. Besides, the energy band modulation in the vertical heterostructures can be used as a switching vehicle in memristors [233]. The vertical stacking allows for high integration density, where each layer can be individually addressed, enabling the realization of artificial synapses and neurons with tunable properties.

##### Recent Progress

(I) *Material Combinations* various combinations of 2D materials have been explored to create vertical heterostructures with tailored properties. For instance, combining graphene with TMDs like MoS_2_ or WSe_2_ provides a platform where graphene acts as an electrode, and TMDs serve as the active channel, allowing for efficient charge transfer and memory effects [212]. Additionally, combining two different TMDs, for example, WS_2_ and MoS_2_, allows to use the band modulation region to release or block the carrier in the passages, enabling switching of vertical heterostructure-based memristors. Figure 9k, l shows that the band modulation by heterostructure is like a “lift gate” on the expressway for the charge carriers and effectively avoids direct “damage” to the memristor layer [233].
(II) *Synaptic Devices* Recent advancements include the development of synaptic devices which exhibit analogue memory states using vertical heterostructures, enabling the emulation of synaptic weights in neural networks [233]. These structures can mimic short-term and long-term plasticity, crucial for learning and memory in neuromorphic systems. For example, as shown in Fig. 9m, Li et al. oxidized the surface of PdSe_2_ with UV-ozone to synthesize PdSeO_x_/PdSe_2_ heterostructure to utilize the point defects (Pd and Se vacancies) for diffusion channels in a vertical memristor [234]. Figure 9n shows that CBA can be fabricated with PdSeO_x_/PdSe_2_ heterostructure to implement convolutional image processing. In addition, vertical heterostructures have been used to fabricate devices that mimic the firing behaviour of neurons [235]. By integrating different 2D materials, researchers have realized devices that can generate action potentials, integrate synaptic inputs, and exhibit threshold behaviour, closely resembling biological neurons.
(III) *In-Memory Computing* Vertical heterostructures facilitate in-memory computing, where data processing and storage occur in the same device. This approach reduces latency and power consumption, as the data does not need to be transferred between memory and processing units. Sun et al. has constructed heterostructures of h-BN/WSe_2_/h-BN as a transistor. The device can be switched between the p- and n-type mode, exhibiting subthreshold swing of 64 mV dec^−1^ and on/off current ratio approaching 10^8^. Implementation of reconfigurable linear logic operations of NOR, RNIMP, NIMP, AND, NAND, IMP, RIMP, and OR directly within these structures [235].
(IV) *Flexible and Transparent Devices* The use of 2D materials allows for the creation of flexible and transparent neuromorphic devices. This opens up applications in wearable technology, electronic skin, and brain–machine interfaces, where the devices can conform to non-flat surfaces or be integrated into displays.

In addition, vertical heterostructure devices offer additional advantages by combining multiple 2D materials within a single stacked architecture. These structures can simultaneously exploit charge trapping, ferroelectric switching, tunnelling, optoelectronic modulation, and interlayer coupling, enabling more complex synaptic functions such as multistate memory, optical plasticity, and gate-tunable learning behaviour. Furthermore, vertical heterostructures can improve leakage suppression and enhance retention characteristics, making them promising for multifunctional neuromorphic systems. However, challenges remain in achieving uniform interlayer interfaces, scalable fabrication, and reproducible performance across large arrays.

### Challenges in Fabrication: Addressing Variability, Scaling, and Integration Issues in Neuromorphic Devices

The fabrication of neuromorphic devices using 2D materials presents a unique set of challenges that must be addressed to harness their full potential. These challenges primarily revolve around variability, scaling, and integration issues, which are critical for the practical application of these devices in large-scale neural networks and computing systems.

**Fig. 10:**
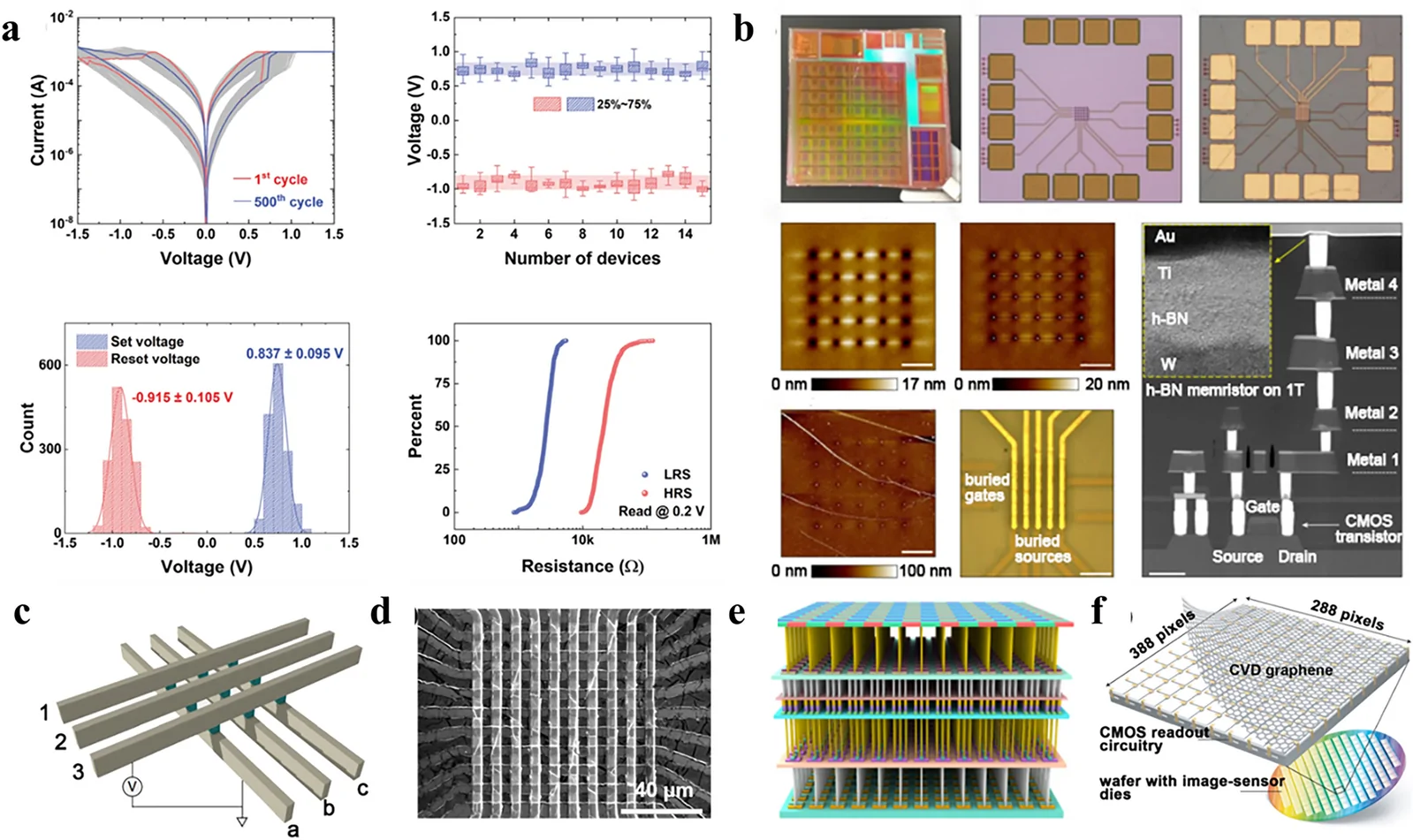
**a** Results indicating cycle-to-cycle and device-to-device variability of memristors [246]. **b** Fabrication of hybrid 2D-CMOS memristive microchips. In the characterizations. the atomic force microscopy of the vias in the 5 × 5 crossbar arrays on the wafers and the cross-sectional transmission electron microscope image of the 1T1M cell in the crossbar array [231]. **c** Depiction of the crossbar array with crossing top electrodes and bottom electrodes [199]. **d** Scanning electron microscope image of a 10 × 10 crossbar array of metal/hNB/metal memristors [199]. **e** Illustration of interconnections between different layers of 2D materials and chips [247]. **f** Depiction of interfacing of transferred CVD graphene and the 3D image sensor read-out circuity on silicon wafer [248]. Reproduced with permission. Copyright 2017, 2018, Springer Nature

Variability in 2D-material-based devices—including cycle-to-cycle (C2C) and device-to-device (D2D) fluctuations—remains a major challenge affecting the reproducibility and stability of memristive performance. For instance, Fig. 10a exhibits the typical C2C, D2D variability in the h-BN RRAM by Jo et al. [246]. The device variability poses significant challenges for the application in reality. Ensuring material uniformity across large areas is essential, as variations in material properties can lead to inconsistent electrical properties, affecting device performance and reliability. Techniques like CVD and epitaxial growth need refinement to produce uniform, high-quality 2D material films. Additionally, the interface between 2D materials and metal contacts can introduce variability due to the formation of Schottky barriers or poor adhesion, requiring engineering solutions to minimize contact resistance. Controlled doping or functionalization of 2D materials to achieve desired electronic properties often results in variability, requiring precise methods for uniform modification at the atomic scale [209].

Scaling down these devices while maintaining their desired properties presents another challenge. As devices are miniaturized, the effects of defects, edge states, and quantum confinement become more pronounced, potentially altering device behaviour [62, 249]. Achieving high integration density without compromising functionality is crucial for neuromorphic computing, which involves reducing the size of individual devices as well as managing interconnects and ensuring dense packing. Compatibility with current CMOS technology is key to realize large-scale integration and applications. Recently, as shown in Fig. 10b, Zhu et al. reported significant progress to fabricate h-BN-CMOS microchips. The CMOS transistors provide outstanding control over the currents across the h-BN memristors, allowing for endurances of roughly six million cycles in memristors as small as 0.053 μm^2^. STDP signals were measured for implementation of spiking neural networks [231]. Crossbar Array (Fig. 10c, d), representing a simple structure that can integrate multiple-layer array structures in large scale, is a highly promising architecture for application in reality. The leakage current and the connection resistances are the most pressing issues yet to be resolved. In large crossbar arrays, sneak-path currents can lead to inaccurate weight updates and degraded read accuracy, making selector devices such as transistors, diodes, or threshold switches necessary. Furthermore, peripheral circuitry including ADCs, DACs, sense amplifiers, and control units may occupy a significant fraction of the total chip area and power consumption, limiting the practical scalability of large neuromorphic systems. Thermal management also becomes critical as devices shrink, with 2D materials offering excellent in-plane thermal conductivity yet facing challenges in vertical heat dissipation in stacked structures.

Integration challenges further complicate the fabrication process. Compatibility with existing semiconductor manufacturing processes is not straightforward, as it requires the development of fabrication techniques that can withstand the harsh conditions of traditional semiconductor fabrication while maintaining the integrity of 2D materials [199]. As shown in Fig. 10e, interconnects and wiring for 2D materials-based circuits require ad hoc solutions, because traditional interconnect techniques might not be optimal due to issues like insufficient integration density and contact resistance at interfaces [247]. Heterogeneous integration, where different 2D materials or different dimensional materials are combined, presents challenges in terms of material compatibility, interface engineering, and ensuring consistent performance across different materials. Figure 10f shows CVD-grown graphene and CMOS read-out circuitry hetero-integrated to form image sensor dies on CMOS wafer [248]. Additionally, packaging and encapsulation are also crucial to protect 2D materials from environmental factors like oxidation or degradation while preserving their electrical properties [250].

Recent progress in addressing these challenges includes the refinement of advanced fabrication techniques like ALD [240], MBE [193, 200], and transfer printing [251], which aim to improve the quality and uniformity of 2D material films, thereby reducing variability [250]. In situ characterization during fabrication, typically referred to as *in operando* techniques, helps control growth and minimize defects. Materials engineering strategies, such as defect engineering, strain engineering, or the creation of hybrid materials, are being explored to address variability and scaling issues. Hybrid integration approaches are also being developed, whereby 2D materials are integrated with traditional silicon technology or other materials to leverage the strengths of both while addressing integration challenges [251–253].

While 2D materials offer immense potential for neuromorphic computing, overcoming fabrication challenges related to variability, scaling, and integration is essential for their practical implementation. Significant research and development efforts are still required to refine fabrication techniques, explore new materials, and develop strategies for integration, ensuring that these materials attain their true potential in enhancing the performance of neuromorphic devices.

## Machine Learning-Driven Optimization of Neuromorphic Devices

### Training and Inference Techniques: Implementing Supervised, Unsupervised, and Reinforcement Learning Algorithms in 2D-Material-Based Neuromorphic Systems

**Fig. 11:**
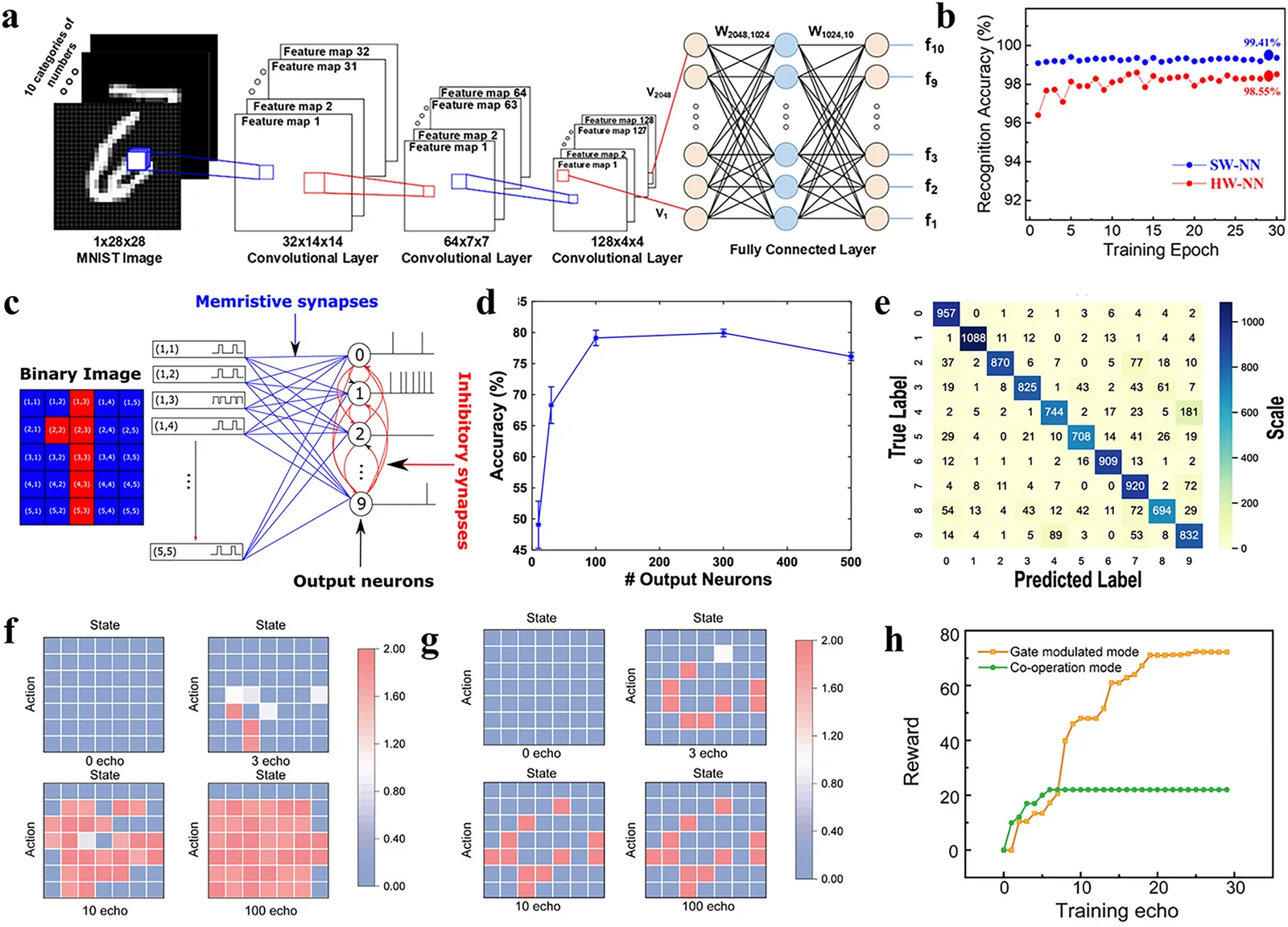
**a** Architecture of the convolutional neural network model used to evaluate the accuracy of the MNIST dataset. **b** A comparison of recognition accuracy between hardware-based neural networks (HW-NN) and software-based neural networks (SW-NN), emphasizing the performance improvements enabled by 2D-material-based memristors. Reproduced with permission from Ref. [244]. **c** A biologically inspired neuromorphic computing mechanism designed for unsupervised character recognition. **d** Classification accuracy as a function of the number of output neurons, measured after a single training epoch for handwritten digit recognition on the MNIST dataset; **e** A confusion matrix illustrating classification accuracy for individual digits, highlighting the most and least accurately recognized digits. Reproduced with permission from Ref. [255]. **f** Memory matrix update process of the agent under the gate-modulated mode during reinforcement learning. **g** Memory matrix update process of the agent under the cooperative mode during reinforcement learning. **h** Rewards obtained by the agent as a function of training epochs in a maze game, demonstrating the rabbit’s probability of finding a water source under the two modes across multiple training epochs. Reproduced with permission from Ref. [256]

In neuromorphic hardware, machine-learning algorithms, including supervised, unsupervised, and reinforcement learning, serve as critical enablers for efficient training and inference [1–3, 33]. These algorithms allow neuromorphic systems to replicate biological processes, significantly enhancing their capabilities in tasks such as image recognition, decision-making, and anomaly detection. Supervised learning relies on labelled data to train models for accurate outcome predictions. Neuromorphic systems implement this learning paradigm through synaptic devices, which follow rules like STDP to adjust synaptic weights based on spike timing. STDP-based learning is especially relevant for SNNs because the relative timing between pre-synaptic and post-synaptic spikes determines the synaptic update, directly mimicking biological event-driven learning. Among the various synaptic devices, memristors fabricated with 2D materials have shown exceptional promise in emulating and storing synaptic weights [71, 254]. These devices dynamically adjust their conductance states during the training process, analogous to the weight updates in artificial neural networks. The gradual and linear conductance modulation of these devices is particularly important for CNN and ANN implementations because it enables accurate multiply-and-accumulate operations during feature extraction and classification. In addition, multilevel conductance states improve weight precision and reduce training error in hardware neural networks. In such systems, algorithms such as gradient descent iteratively minimize the error between predicted and target outputs, while backpropagation propagates this error backward through the network to fine-tune each memristive synapse’s conductance. This enables hardware-level learning and adaptation directly within the memristor array. For example, MoS_2_-based memristors were used as synaptic devices demonstrating linear and distinct synaptic behaviours [244]. These memristors were subsequently integrated into artificial neural networks, achieving an impressive 98.55% recognition accuracy on the MNIST dataset using a deep neural network (DNN). Figure 11a illustrates the baseline CNN (convolutional neural network) architecture employed in the simulation, consisting of three convolutional layers—which extract features from input images by applying small trainable filters—and two fully connected layers. The model was trained over 30 epochs (i.e. complete passes through the entire training dataset), with convolutional layers performing operations between the input feature maps and kernels. The recognition accuracy of the hardware neural network (HW-NN) reached 98.55%, closely approximating the software neural network (SW-NN) accuracy of 99.41%, as shown in Fig. 11b, highlighting the potential of 2D-material-based memristors in advancing neuromorphic systems for high-performance computing tasks. On the other hand, unsupervised learning plays a pivotal role in identifying patterns within unlabelled datasets, making it essential for clustering and anomaly detection tasks. Techniques such as Hebbian learning, which strengthens the connection between neurons that activate simultaneously (“cells that fire together, wire together”), and autoencoders, which use unsupervised neural networks to compress and reconstruct input data by extracting latent features, are widely implemented in neuromorphic hardware to enable self-organized pattern recognition and feature learning. The unique multiconductance state properties of memristors have been shown to significantly improve clustering accuracy compared to traditional architectures when processing complex datasets. In a notable example, a graphene-based memristive device was employed as synapse within SNNs to realize STDP for efficient unsupervised learning [255]. In this case, the combination of STDP, multilevel conductance states, and lateral inhibition enabled efficient spike-based competition between neurons, which is particularly important for unsupervised SNN learning and clustering tasks. To enhance competitive Hebbian learning among neurons, inhibitory synapses were integrated between all output neurons, enabling lateral inhibition, as illustrated in Fig. 11c. This configuration introduced a biologically inspired competitive mechanism, optimizing the network’s ability to distinguish patterns. To evaluate performance, the network was trained on the original 60,000 images from the MNIST training set and tested on 10,000 images from the MNIST test set. After a sample training phase, the synaptic receptive fields demonstrated the network’s capacity to learn handwritten digits. This was reflected in the high average test-set classification accuracy, which was approximately 80%, as depicted in Fig. 11d. The results were averaged over ten trials, each conducted with networks containing different numbers of output neurons. A detailed analysis using the confusion matrix—a tabulated representation of predicted versus actual classifications—further revealed the recognition accuracy for individual digits. The digits “0” and “1” were the most accurately classified, while “3”, “4”, and “9” exhibited the highest misclassification rates, as shown in Fig. 11e, addressing the issue of a relatively high current draw in the memristors’ on-state by employing current limiters/selectors, thus providing a practical solution to mitigate power consumption challenges in large-scale implementations. Unlike supervised and unsupervised learning, reinforcement learning (RL) optimizes actions through interactions with the environment. Various algorithms, such as the Markov Decision Process (MDP), Q-learning, and policy gradients, dynamically adjust synaptic weights to improve performance. In this context, a 2D ferroelectric α-In_2_Se_3_-based three-terminal neuromorphic device was developed to modulate conductance states, enabling brain-inspired learning strategies for real-world applications [256]. The multistate conductance behaviour and non-volatile memory retention of the ferroelectric device are particularly useful for reinforcement learning because they allow the system to preserve reward-dependent weight updates over repeated training cycles. Two operation modes, gate-modulated mode and co-operation mode, were implemented and compared to execute the MDP algorithm in artificial intelligence. In the gate-modulated mode, applying a −2 V pulse at the gate terminal, along with a 1.5 V pulse at the source terminal, changed the device’s conductance. This dynamic adjustment served as a reward signal in a maze game, where a rabbit needed to locate a water source within fifteen steps. Figure 11f illustrates the synaptic weight matrix, trained over 100 epochs. Out of 100 tests, the probability of the rabbit successfully finding the water source was 68%. To further enhance the success rate, the co-operation mode was employed. In this mode, voltage pulses from the source terminal were used to read the conductance state as well as to modify the channel conductance. The memory matrix was updated and trained across 100 epochs, leading to an increase in the rabbit’s success probability to 82%, as shown in Fig. 11g. Additionally, Fig. 11h depicts the variation of reward values over time across different modes, demonstrating faster convergence in the co-operation mode. This highlights the potential of co-operation mode to significantly improve the learning efficiency of MDP in artificial intelligence applications. Thus, advancements in training techniques underscore the potential of neuromorphic devices for high-performance and reliable computing, paving the way for their broader application in artificial intelligence and practical problem-solving.

### Adaptive and Low-Power Architectures: Exploiting Machine Learning for Energy-Efficient Design and Operation

**Fig. 12:**
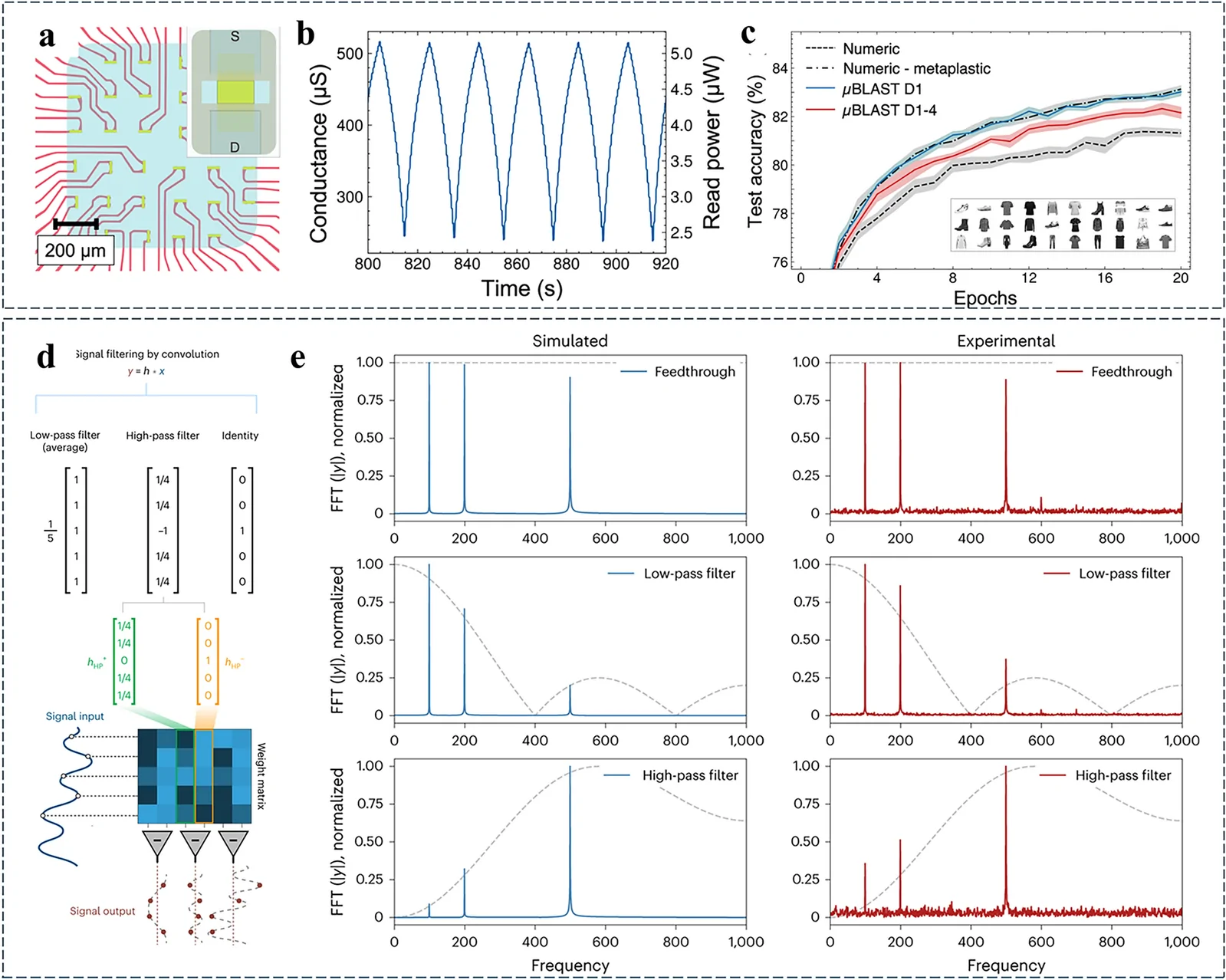
**a** Schematic representation of the 32 µBLASTs array, highlighting metal feedlines (red), the graphene channel (green), passivation with polyimide, and coverage with Nafion (blue). **b** Synaptic behaviour observed in µBLASTs, demonstrating low read power and high energy efficiency during operation. **c** Online training simulation of a multilayer perceptron using µBLAST devices, applying experimental data to neuromorphic tasks on the Fashion-MNIST dataset (clothing articles) [**a**-**c**] reproduced with permission from Ref. [259]. **d** Convolution-based signal processing for different filters (low-pass, high-pass, and identity) within the architecture of a 32 × 32 integrated vector–matrix multiplier based on monolayer MoS₂, illustrating in-memory computing [260] **e** Fast Fourier Transform (FFT) analysis comparing simulated and experimental signal processing results, confirming system accuracy. Reproduced with permission from Ref. [260]

The increasing demand for energy-efficient systems in neuromorphic computing has driven significant advances in adaptive and low-power architectures, which integrate circuit-level and algorithmic strategies that dynamically regulate power consumption. These systems employ energy-aware control schemes, such as voltage scaling, current limiting, and adaptive biasing, to maintain high computational efficiency while minimizing energy waste. These systems are pivotal for ensuring sustainable and high-performance operations, especially in applications like wearable devices and IoT sensors [257, 258]. Exploiting the unique properties of 2D materials such as low leakage currents, tuneable electronic states, and high flexibility, these architectures dynamically manage energy consumption to meet varying computational workloads [257]. In addition, ML algorithms can dynamically regulate power in neuromorphic systems by adjusting energy consumption based on real-time computational demands [254]. This dynamic regulation is achieved through feedback loops that modulate voltage and current supplied to synaptic devices, enabling precise power management. In this context, a biocompatible bilayer graphene-based artificial synaptic transistor (BLAST) array was reported (Fig. 12a), capable of mimicking synaptic behaviour and enabling long-term potentiation, with a switching energy efficiency of ~ 50 aJ µm^−2^ [259]. Furthermore, synaptic behaviour was observed in the μBLASTs (channel dimensions: 40 × 10 μm^2^), and the results showed low read power between approximately 2.5 and 5.0 μW, indicating minimal energy consumption during the data-reading process. Such low-power operation is essential for reducing overall device energy usage in large-scale neuromorphic arrays, as depicted in Fig. 12b. The analogue and nonlinear conductance modulation of µBLAST devices is especially relevant for multilayer perceptrons and CNN-type architectures, where gradual weight updates are needed for accurate training and inference. Because of this, the unique nonlinear and asymmetric updates of µBLASTs resulted in slower online training of a multilayer perceptron on UCI-HAR compared to ideal synaptic updates. However, on Fashion-MNIST, µBLASTs significantly outperformed ideal numeric weights in training performance (Fig. 12c). This improvement was more pronounced when the measured synaptic properties of a single device (D1) were used rather than the variations across four devices (D1–D4), highlighting D1’s superior plasticity. These biocompatible devices, which operated at low energy density (< 50 aJ µm^−2^) and > 10 kHz speeds, demonstrated great potential for applications in bioelectronics and neuromorphic computing. Concerning large-scale energy-efficient architectures, data-driven algorithms like signal processing and ML are essential for handling massive data, but the limitations of the von Neumann architecture, with its separation of processing and memory, necessitate the development of in-memory computing. In this regard, a 32 × 32 integrated vector–matrix multiplier comprising 1,024 floating gate field-effect transistors (FGFETs) based on monolayer MoS_2_ as the channel material was reported, demonstrating multibit data storage with a single programming pulse [260]. Discrete signal processing was implemented using different kernels to enable in-memory computing. The accelerator processed signals by convoluting input signals with kernels, allowing parallel operations for low-pass filtering, high-pass filtering, and feedthrough in a single cycle (Fig. 12d). Kernel values were encoded into memory conductance, and outputs from positive and negative components were subtracted to produce the final processed signal, with scalability limited only by matrix size, as validated by theoretical and experimental comparisons. This type of conductance-based vector–matrix multiplication is particularly suitable for CNN inference because convolution operations can be directly mapped onto crossbar arrays, significantly reducing latency and energy consumption. Additionally, the signal range was restricted to − 100 to 100 mV at *V*_read_ = 0, and the fast Fourier transform (FFT) of simulated and experimental processed signals closely matched theoretical predictions for all three filters (Fig. 12e), demonstrating system accuracy. These findings suggest that large-scale arrays of FGFETs based on 2D materials could be applied to advanced tasks such as image processing and artificial neural network inference.

### Case Studies: Highlighting Recent Studies Where Machine Learning Enhances Synaptic Behaviour, Plasticity, and Performance in 2D-Material-Based Systems

**Fig. 13:**
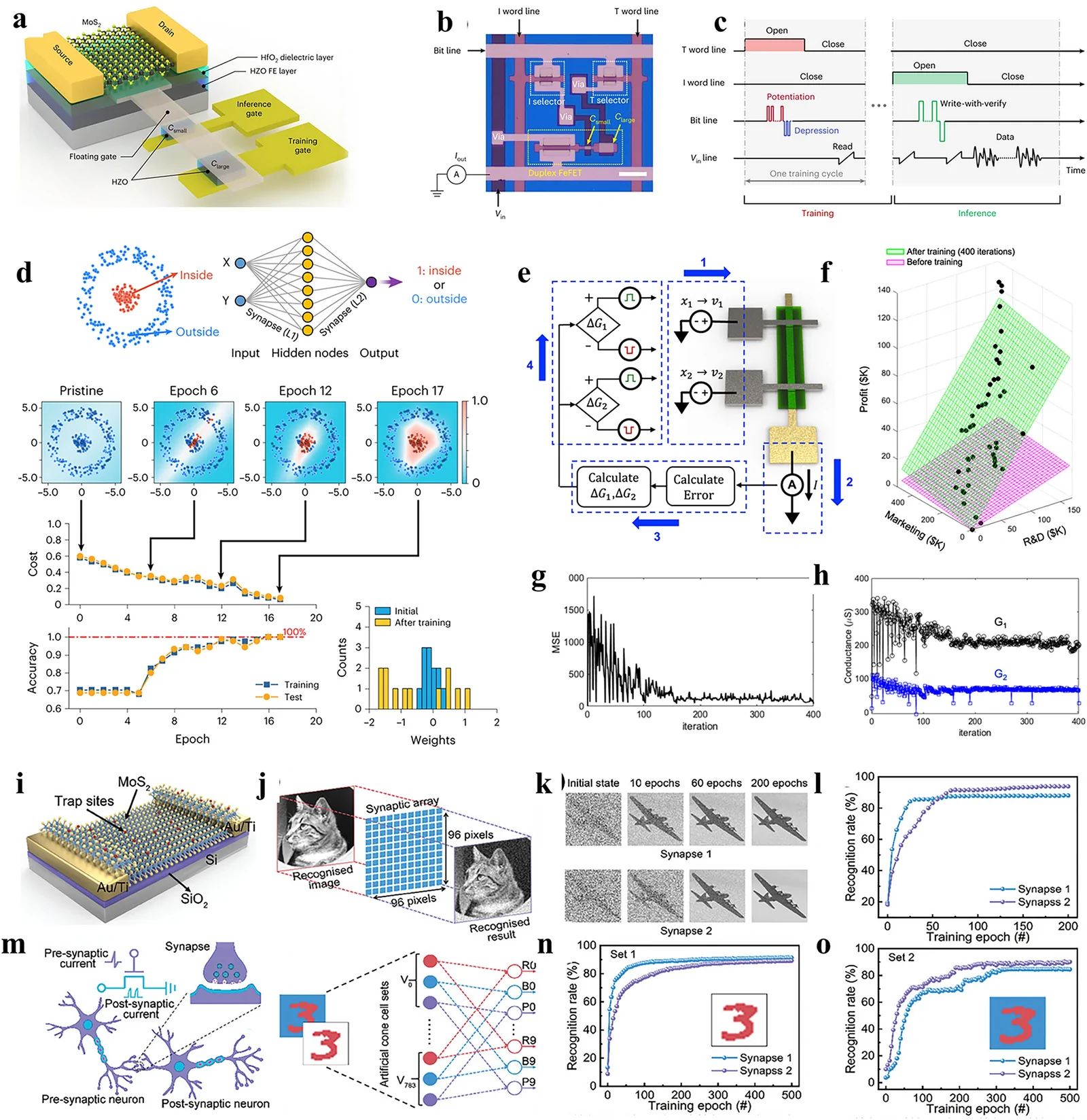
**a** Schematic of a duplex 2D material computing-in-memory device. **b** Optical microscopy image and programming sequence of a TIIO cell comprising 2T1D. **c** In the duplex FeFET core, two selector transistors (T and I) are involved in forming a pseudo crossbar structure. **d** Illustration of the 2D localization task, a nonlinear classification problem requiring a neural network with at least two synapse layers. The target of this ANN was to classify location data as “inside (1)” or “outside (0)” with high accuracy, with cost and accuracy plotted as a function of training epoch (blue for training data and yellow for test data). The training completed at the 17th epoch with 100% accuracy. Reproduced with permission from Ref. [263]. **e** Hexagonal boron nitride (h-BN) memristor arrays performing dot-product operations for multivariable stochastic linear regression. **f** Predictions before and after training on a dataset of 50 start-up companies, illustrating improved regression accuracy. **g** Reduction in mean squared error (MSE) with successive training steps, confirming effective optimization. **h** Changes in conductance during training, demonstrating parameter stabilization after initial fluctuations. Reproduced with permission from Ref. [139]. **i** Structural schematic of the MoS_2_-based transistor and schematic illustration of the biological synapse compared to the MoS_2_-based artificial synaptic device. **j**–**l** Performance of the artificial neural network (ANN) for grayscale image recognition using STL-10 datasets. **m**–**o** Dual-layer optoelectronic neural network (ONN) based on MoS₂ transistors for coloured-digit recognition, showing accuracy improvements across datasets. Reproduced with permission from Ref. [264]

Recent advancements in 2D-material-based synaptic systems, integrated with ML, have resulted in significant improvements in synaptic behaviour, adaptive plasticity, and overall system performance [261]. By addressing the limitations of traditional computing architectures, such as the von Neumann bottleneck, these innovations provide localized, energy-efficient AI processing and pave the way for biologically inspired, real-time technologies [262]. One prominent case involves a duplex device structure integrating FeFETs with an atomically thin MoS_2_ channel, demonstrated as a universal in-memory computing architecture for in situ machine learning (Fig. 13a) [263]. This architecture employs arrays of two-transistor–one-duplex (2T1D) FeFET cells, in which one transistor controls the training operation and the other manages inference, while the duplex FeFET element serves as a shared memory node. Such a configuration enables simultaneous weight storage and signal processing within the same cell, forming the basis of hardware neural network implementation. The multilevel conductance states and non-volatile memory characteristics of the duplex FeFET are particularly beneficial for ANN training because they enable repeated weight updating while preserving stored information during inference. Each 2T1D cell featured distinct ferroelectric (FE) and dielectric (DE) capacitance ratios (CFE/CDE), independently optimized for training and inference operations. An optical image of the 2T1D duplex cell is shown in Fig. 13b, highlighting the split gates, which are connected to the training (T) and inference (I) selectors through vertical interconnects (vias). During in situ learning, T and I word lines corresponding to the respective gate voltages selectively activated T-type synapses during training and I-type synapses during inference. Figure 13c illustrates the programming sequence, which efficiently integrates training and inference functionalities within a single device. A multilayer ANN comprising input, hidden, and output layers was implemented using an 8 × 3 array of 2T1D cells, as depicted in Fig. 13d [263]. T-type synapses were used during training, with accuracy and loss gradually converging over successive epochs as dataset boundaries became more well-defined. Heat maps of the classification results at the 6th, 12th, and 17th epochs (Fig. 13d) highlighted the learning progression. By the 17th epoch, both training and test data achieved 100% accuracy, with costs reduced to 0.067 and 0.083, respectively. The histogram of synaptic weights before and after training confirmed effective weight adjustments via the backpropagation algorithm. These findings underscore the potential of duplex FeFET-based architectures for integrating neuromorphic computing cores with functional modules, such as pooling, activation, routing, and buffering, to advance edge intelligence applications. Another noteworthy case study demonstrated the application of h-BN memristor arrays for performing dot-product operations, a fundamental function in neural networks. These operations were implemented by applying input voltages (*V*_1_, *V*_2_) to memristor conductances (*G*_1_, *G*_2_), generating output currents at the post-synaptic neurons (Fig. 13e) [139]. The analogue conductance modulation of h-BN memristors is especially important for regression and ANN models because it enables accurate dot-product operations and gradual weight updates during training. The h-BN memristor arrays were used to train a multivariable stochastic linear regression model. Using a dataset of 50 start-up companies, the model predicted profits based on marketing and R&D investments, with input variables normalized between 0 and 0.15 V. Figure 13f shows predictions before (magenta plane) and after training (green plane) over 400 iterations, where significant improvements were observed [139]. Quantitative results in Fig. 13g illustrate the reduction in mean squared error (MSE) with successive training steps, confirming algorithmic convergence. Additionally, changes in conductances *G*_1_ and *G*_2_ during training (Fig. 13h) highlight larger fluctuations during initial steps, followed by stabilization, indicating effective optimization of the model parameters. Further addressing the von Neumann bottleneck, an MoS_2_-based anisotropic synaptic device was demonstrated as an axon-multisynapse system, enabling brain-inspired tasks such as image recognition and coloured-digit identification through artificial neural networks (ANNs) [264]. Figure 13i presents the back-gated transistor structure based on MoS_2_, where the artificial synapse mimics biological synaptic functions by generating post-synaptic currents (PSCs) in response to pre-synaptic gate pulses. By inducing anisotropic synaptic plasticity through localized electron beam irradiation (EBI), this system facilitated diverse neuromorphic applications. The direction-dependent conductance behaviour of the MoS_2_ synapse is particularly useful for CNN-based image recognition because it provides different trade-offs between rapid learning and high final accuracy. Using this anisotropic synaptic plasticity, an ANN was developed to process greyscale images from the STL-10 dataset (96 × 96 pixels). As shown in Fig. 13j, feature maps were extracted via an 18-layer convolutional neural network (ResNet18) and classified through a fully connected network.

During training, the ANN exhibited distinct advantages based on synaptic direction. One direction provided rapid initial learning, as illustrated by object outlines emerging after fewer epochs (Fig. 13k), while the other achieved higher final recognition accuracy (Fig. 13l), highlighting a trade-off between speed and precision. Finally, a dual-layer optoelectronic neural network (ONN) based on anisotropic MoS_2_ transistors was developed for coloured-digit recognition tasks (Fig. 13m) [264]. The input layer comprised 784 cone cell sets, each responsive to red, blue, and purple light, generating synaptic dynamics mapped across R/B/P weight regions. The ONN classified coloured MNIST digits into categories such as R0–R9, B0–B9, and P0–P9. For datasets without distracting backgrounds (Set 1), synaptic plasticity enabled 83.11% accuracy after 50 learning epochs for one synapse, as shown in Fig. 13n, while the other achieved 69.75%. For datasets with distracting backgrounds (Set 2), the second synapse outperformed the first, reaching 85.31% accuracy compared to 69.86% after 200 epochs (Fig. 13o) [264]. These findings demonstrate the ONN’s robust pattern recognition capabilities, even in the presence of visual distractions. In addition, these case studies collectively demonstrate the transformative potential of 2D-material-based synaptic devices integrated with machine learning to enhance neuromorphic computing. By achieving energy-efficient, adaptive, and high-precision performance, these innovations pave the way for future applications in edge AI and real-time data processing, while addressing scalability and integration challenges for widespread implementation.

## Performance Metrics and Benchmarking

### Responsivity and Efficiency: Evaluating the Electrical and Optical Performance of Neuromorphic Devices Made with 2D Materials

**Fig. 14:**
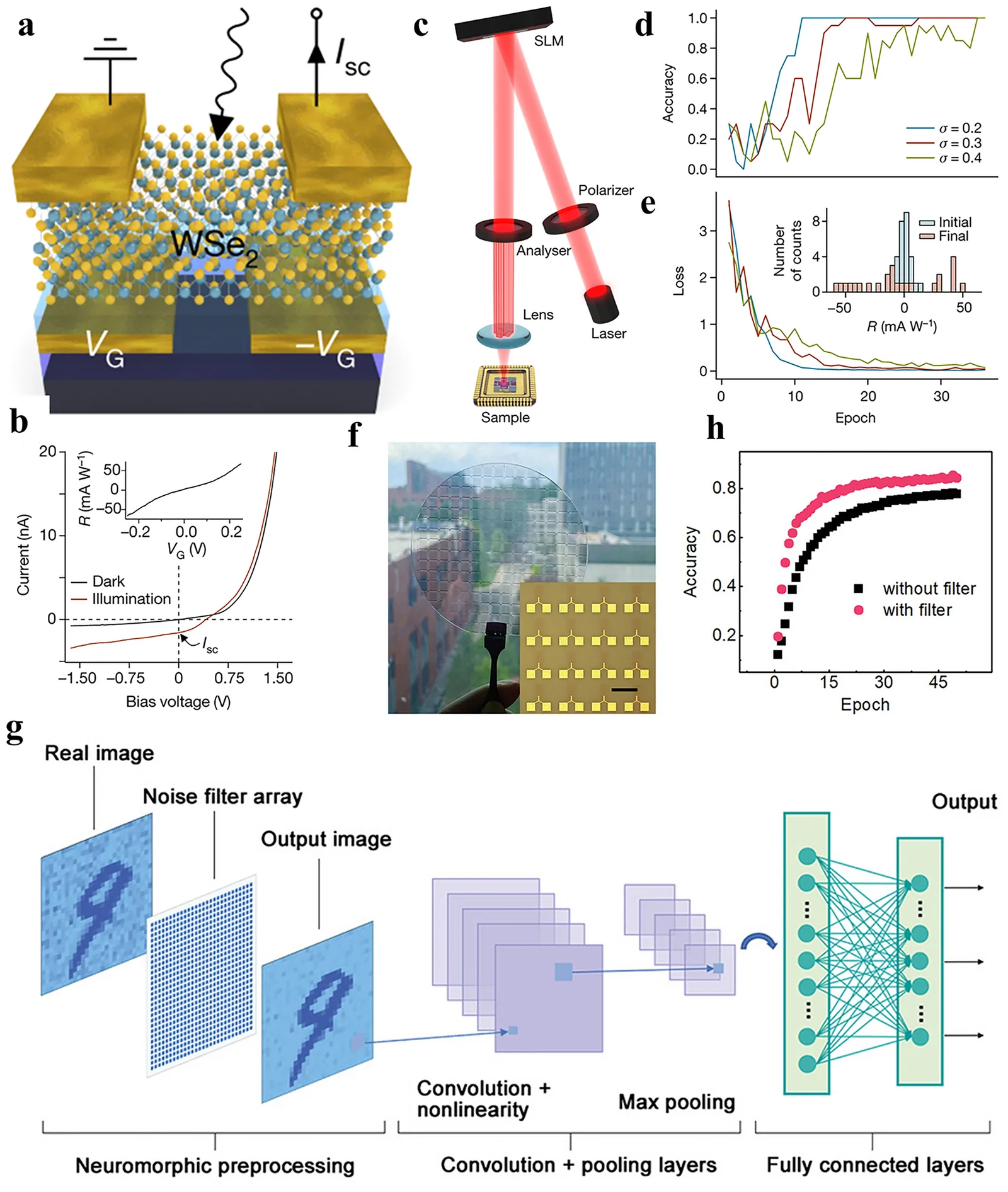
**a** Reconfigurable 2D semiconductor photodiode array constructed using lateral p–n junction photodiodes based on WSe_2_. **b** Current–voltage characteristic curve of a photodetector in the dark (blue line) and under optical illumination (red line), with the inset showing gate-voltage tunability of photoresponsivity. **c** Scheme of the optical setup: laser light is linearly polarized by a wire-grid polarizer, reflected by a spatial light modulator (SLM), filtered by an analyser for intensity modulation, and projected onto the photodiode array. **d**–**e** Performance metrics of the ANN during training, depicting loss reduction and accuracy improvements under various noise levels. Reproduced with permission from Ref. [269]. **f** Photograph of a transparent wafer-scale MoS_2_ FET on sapphire, with the inset showing a magnified image of the transistors. **g** Illustration of an artificial neuromorphic visual system utilizing the MoS_2_ synapse for image noise filtering (preprocessing) and a CNN for image recognition. **h** Image recognition rate and accuracy with and without noise filtering. Reproduced with permission from Ref. [270]

Due to their exceptional electrical, optical, and mechanical properties, 2D materials provide an ideal platform for developing neuromorphic synaptic devices. Their unique characteristics, such as high carrier mobility, tuneable bandgaps, and strong light-matter interactions, enable efficient data processing and signal transmission in edge computing and wearable technologies [265]. Synaptic devices built with 2D materials mimic human brain functions by combining computation and memory capabilities, paving the way for real-time data processing in resource-constrained environments [266]. Specifically, neuromorphic synaptic devices based on 2D materials drastically improve energy efficiency and operational speed while simultaneously offering the flexibility and scalability required for next-generation computing [73, 267]. The integration of these devices into flexible and portable platforms addresses critical challenges in developing sustainable and high-performance neuromorphic systems. The responsivity and efficiency of these devices play a pivotal role in determining their suitability for applications such as autonomous systems, edge AI, and real-time data processing [268]. A reconfigurable 2D semiconductor photodiode array was employed to integrate sensing and processing directly within an ANN [269]. This device exploited a continuously tuneable photoresponsivity matrix to encode synaptic weights, enabling both supervised and unsupervised learning. Optical images projected onto the chip were classified and encoded with a throughput of 20 million bits per second, significantly enhancing processing efficiency. The photodiode array was constructed using lateral p–n junction photodiodes based on WSe_2_ (Fig. 14a), a 2D semiconductor with ambipolar conduction behaviour and excellent optoelectronic properties. The device featured a thickness of approximately 4 nm and utilized split-gate electrodes with a ~ 300-nm-wide gap to control the responsivities. By applying gate voltages of ± V_G_, the photoresponsivity could be tuned between − 60 and + 60 mA W^−1^, as illustrated in Fig. 14b [269]. This adjustability facilitated precise training of synaptic weights and efficient responsivity modulation, a critical factor for enhancing neuromorphic computation. To evaluate the device’s performance, optical images were projected onto the array using a setup optimized for a wavelength of 650 nm and an irradiance of 0.1 W cm^−2^ (Fig. 14c) [269]. This configuration allowed for efficient signal processing in the ANN. Training and testing of the network demonstrated remarkable results: as shown in Fig. 14d, e, the loss decreased rapidly over 35 epochs, achieving a minimum within 15 to 35 epochs depending on noise levels (σ = 0.2, 0.3, 0.4) [269]. The accuracy reached 100% for all noise levels, with faster convergence observed in lower noise conditions, revealing the feasibility of implementing analogue deep-learning networks by converting photocurrents into voltages, enabling real-time image recognition and encoding. Such advancements pave the way for ultrafast vision sensors integrated with neuromorphic systems, addressing critical challenges in autonomous systems, edge AI, and other applications requiring high-speed, low-energy computation. An artificial optoelectronic synapse based on MoS_2_ FETs demonstrated the integration of sensing, memorizing, and preprocessing functions (Fig. 14f) [270]. The MoS_2_-based opto-synaptic device realized basic synaptic functions, such as paired-pulse facilitation (PPF) and the transition from short-term memory (STM) to long-term memory (LTM), achieved by varying gate voltages and light illumination. Additionally, the device successfully simulated interest-modulated human visual memory via gate-voltage modulation. A CNN was employed to recognize images filtered for background noise. The CNN architecture consisted of a convolutional layer, a max-pooling layer, and an output layer, as illustrated in Fig. 14g. The MNIST database was used for simulations, and images processed by the MoS_2_ opto-synaptic device achieved higher recognition accuracy (85.5%) compared to those without the filtering process (79.5%), as depicted in Fig. 14h [270]. Moreover, the learning rate for filtered images improved significantly, requiring fewer training iterations to reach convergence. These advancements collectively underscore the transformative potential of 2D materials in creating scalable, energy-efficient, and high-performance neuromorphic systems, promising significant contributions to the fields of artificial vision, edge AI, and beyond [270].

### Energy Consumption: Analysis of Power Efficiency, Focusing on Low-Power Operation Suitable for Edge and Wearable Applications

**Fig. 15:**
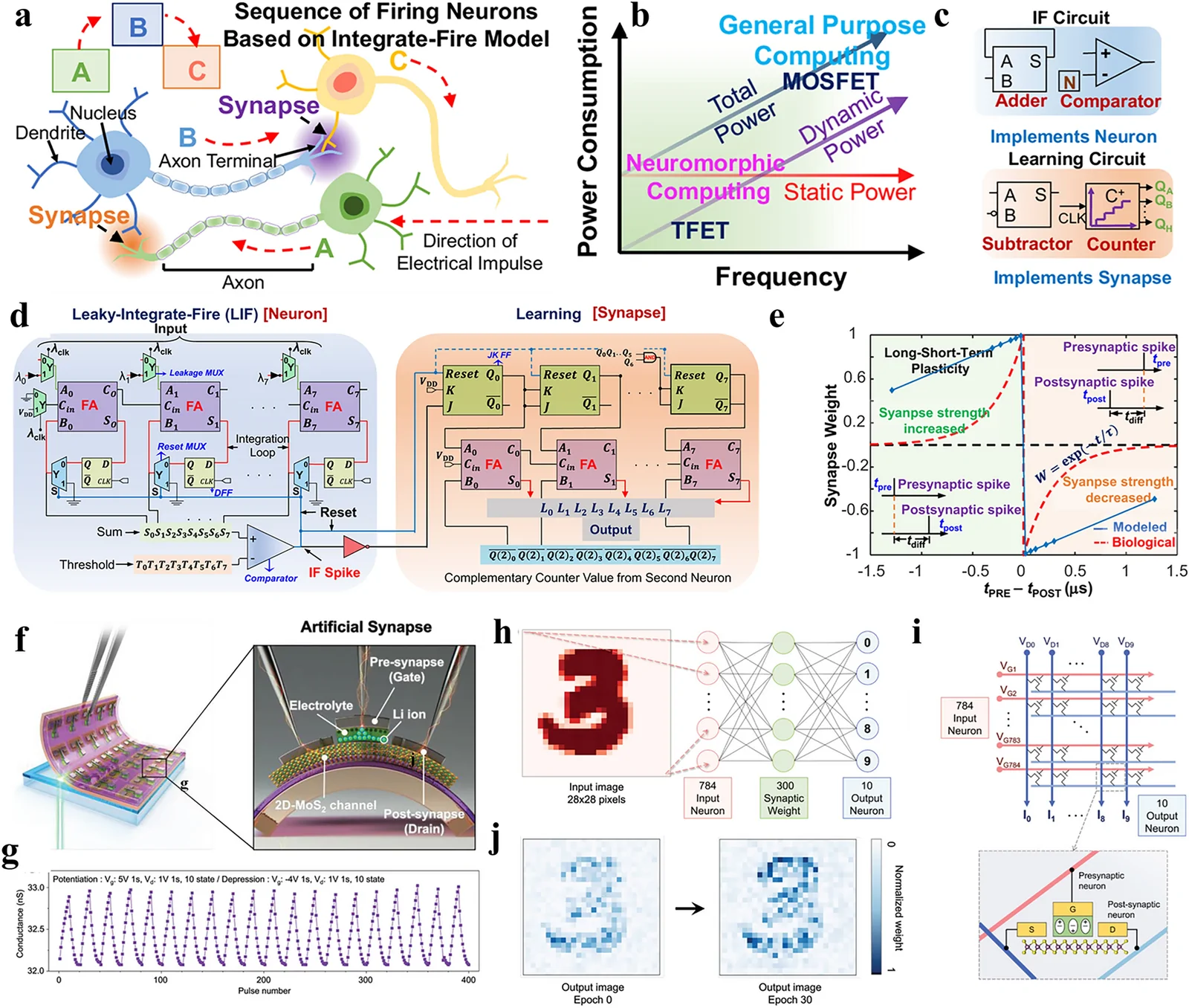
**a** Illustration of three firing neurons (A, B, C) in the brain and their synaptic connections. **b** Operating power consumption of a circuit as a function of clock frequency, showing that dynamic power dissipation is lower for low-frequency neuromorphic circuits that mimic neuronal and synaptic behaviour in the brain. **c** IF circuitry (centre) for neuron firing, implemented using a feedback loop adder and comparator circuit with a firing threshold of N, while neural learning (right) is achieved through synaptic behaviour using a subtractor and a counter circuit (C + represents an up-counter, with Qs as the output). **d** Schematic of the neuromorphic circuit architecture for a single firing neuron, including Leaky-Integrate-and-Fire (LIF) circuitry (left) and Hebbian learning circuitry (right). **e** Simulation of long short-term plasticity (solid blue line) compared with the biological model (dashed red line). Reproduced with permission from Ref. [272]. **f** Design of a flexible artificial synapse array, with a magnified cross-sectional view of each device. **g** Endurance characteristics of the neuromorphic device over 20 cycles. **h** Schematic of a multilayer neural network structure for recognizing handwritten digits with 28 × 28 pixels. **i** Circuit design of a synaptic transistor-based crossbar array for matrix operations, where VG, VD, and I denote the input signal, read signal, and output current, respectively. The inset shows an image of the synaptic transistor. **j** Output images showing progression with different synaptic weights during training epochs from 0 to 30. Reproduced with permission from Ref. [273]

As discussed earlier, the computing and wearable technologies [270, 271] applications demand architectures that prioritize energy efficiency to ensure optimal performance under reliable power constraints. Advanced materials, such as 2D semiconductors including graphene and TMDs, have proven to be key enablers of low-power neuromorphic systems due to their unique characteristics, such as non-volatility, high electrical conductivity, and scalability. Reducing energy consumption in neuromorphic systems requires the integration of innovative techniques, including dynamic voltage scaling, sleep modes, and the use of low leakage materials, to address the strict power limitations of edge and wearable devices [270, 271]. Given this, neuromorphic circuits based on 2D-TMD layered channel material-based tunnel field-effect transistors (TFETs) demonstrated significant improvements in energy efficiency by exploiting their unique properties, such as low OFF-state current and subthreshold swing (SS) values of less than 60 mV/decade, effectively overcoming the limitations of traditional CMOS-based implementations [272]. Figure 15a illustrates an interconnected biological network comprising three neurons (A, B, and C) and their synaptic connections. In this network, spikes generated on the axons of neuron A (or B) are transmitted across synapses to the dendritic terminals of neuron B (or C), increasing their membrane potential and eventually triggering firing events once the firing threshold is exceeded. Figure 15b, c highlights the advantages of TFET-based neuromorphic circuits over CMOS-based designs. These circuits operate at low frequencies (~ MHz) and activity factors due to the sparse firing nature of neurons, making TFETs an ideal choice for low-power applications. The energy efficiency of TFETs is further enhanced by their low static power dissipation and steep turn-on characteristics. Figure 15d presents the architecture of a neuromorphic circuit incorporating a Leaky-Integrate-and-Fire (LIF) neuron and Hebbian learning circuitry. The LIF neuron integrates membrane potential using an 8-bit full-adder (FA) looped through D-flip-flops (DFFs) and multiplexers (MUXs) to enable membrane potential reset post-firing. Meanwhile, the Hebbian learning circuit employs a JK flip-flop-based up-counter to track timing differences between primary and secondary neurons, determining connection strength through signed outputs. This optimized neuromorphic circuit, implemented with 3259 transistors, mimics biological neuronal functions such as spiking, resetting, and synaptic weight adjustments. Further, the simulation of LSTP for a neural synapse (solid blue line) was compared with the biological STDP model (dashed red line), shown in Fig. 15e [272]. The firing time difference (t_DIFF_) between pre-synaptic and post-synaptic neurons was analysed by assuming a fixed firing event for the post-synaptic neuron (t_POST_) and varying the pre-synaptic neuron firing event (t_PRE_). When the pre-synaptic neuron fires before the post-synaptic neuron (*t*_DIFF_ < 0), indicating a causal relationship, the synaptic weight is positive and increases (up to 1) as t_DIFF_ decreases, signifying a stronger connection. Conversely, for anti-causal events (*t*_DIFF_ > 0), where the post-synaptic neuron fires before the pre-synaptic neuron, the synaptic weight decreases (to a minimum of − 1) as *t*_DIFF_ decreases [272]. The sharpness of the simulated STDP curve is influenced by the rate at which the up-counter value increases per clock cycle, while the biological STDP curve follows an exponential decay governed by the response time constant (τ), which determines the sharpness of the synaptic weight decay. These findings highlight the nuanced relationship between firing time differences and synaptic weight modulation in both simulated and biological models, thus revealing the potential of TFET-based systems to enable scalable, energy-efficient, and high-performance neuromorphic computing for real-time applications. Recent advancements in neuromorphic systems have paved the way for significant progress in wearable applications. The ultra-flexible artificial synapse device is a notable example. Fabricated using a 2D MoS_2_ channel and lithium silicate (LiSiO_x_) solid electrolyte, constructed via a laser lift-off process on colourless polyimide substrates, the device is shown in Fig. 15f [273]. This fabrication method ensured durability and adaptability, with the device maintaining stable synaptic characteristics over 20 cycles, as shown in Fig. 15g, and enduring 400 gate pulses without degradation. The novel MoS_2_ and LiSiO_x_ heterostructures facilitated robust intercalation and deintercalation processes, ensuring long-term potentiation and depression performance, critical for real-time low-power neuromorphic edge computing. The robustness of these devices was further assessed through simulations involving a three-layer neural network for handwritten digit recognition using the MNIST dataset (Fig. 15h) [273]. The network architecture, comprising 784 input neurons, 300 hidden neurons, and 10 output neurons, showcased the device’s efficacy in wearable scenarios. Synaptic transistor-based crossbar arrays for matrix operations (Fig. 15i) revealed that the synaptic weights mapped during training epochs achieved recognition accuracies of 94.5%, as shown in Fig. 15j. This demonstrates the potential of these devices for wearable adaptive neuromorphic computing, addressing the unique challenges of edge processing in dynamic environments. Thus, these breakthroughs in neuromorphic system design underscore the potential to revolutionize edge and wearable computing, addressing power limitations while maintaining high performance and adaptability [273].

### Stability and Endurance: Long-Term Performance and Stability in Flexible and Reconfigurable Neuromorphic Systems

**Fig. 16:**
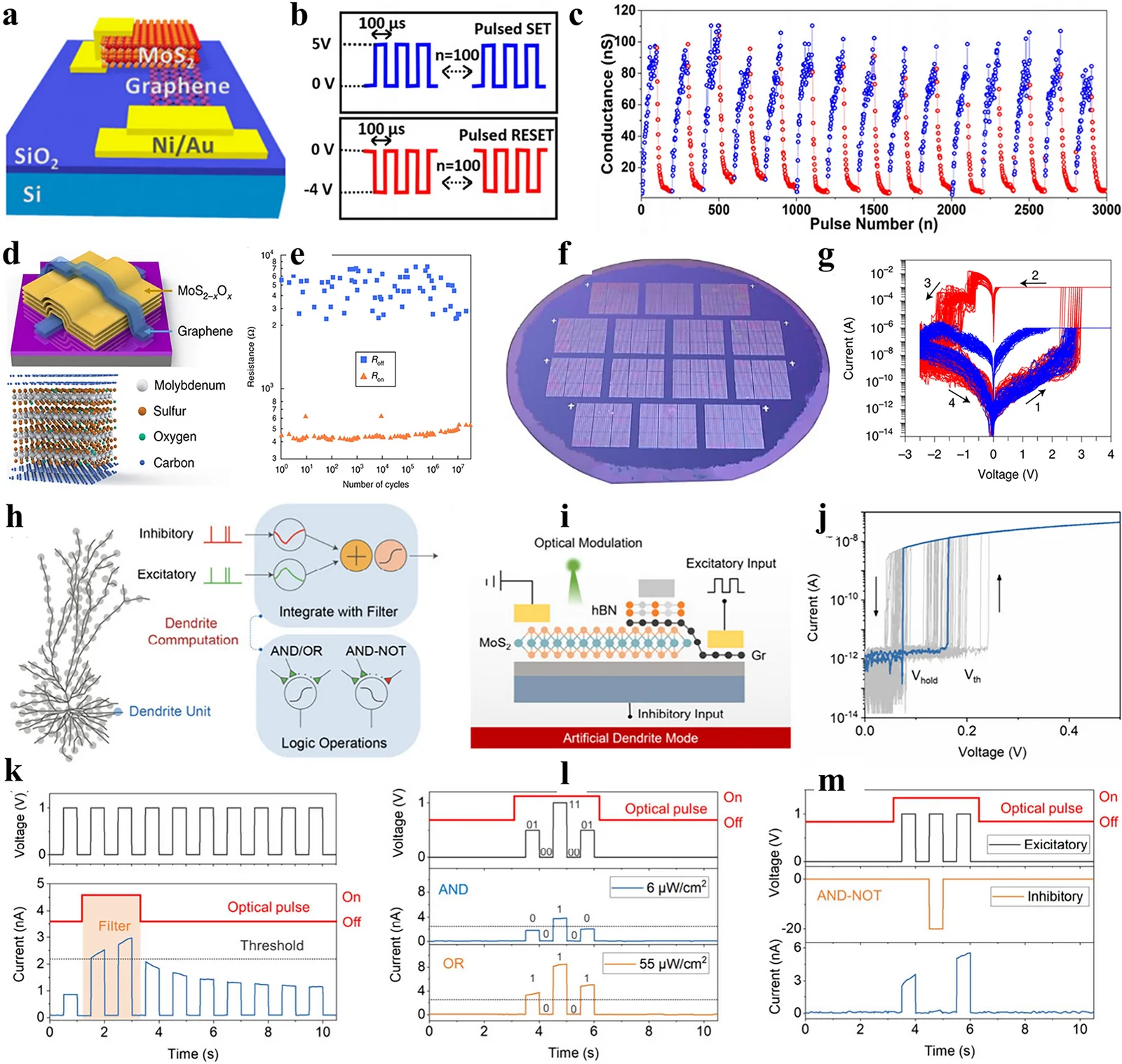
**a** Schematic illustration of the MoS_2_/graphene devices. **b** Pulsed I–V measurements showcasing synaptic behaviour with multiple distinct conductance states. **c** Near-linear weight updates achieved in MoS_2_/graphene devices, underscoring their potential for unsupervised learning applications. Reproduced with permission from Ref. [275]. **d** Schematic illustration of the GMG (Graphene/MoS_2_/Graphene) devices, including the crystal structure of the GMG stack (bottom). **e** Stability and endurance performance of graphene/MoS_2_-xO_x_/graphene memristors under extreme environmental and operational conditions. Reproduced with permission from Ref. [119]. **f** Photograph of a 2-inch wafer featuring Au/h-BN/Au memristive crossbar arrays distributed across its surface. **g** Representative I–V characteristics recorded over 120 cycles from a single Au/h-BN/Au memristor, using ICC = 1 µA (blue lines) and ICC = 1 mA (red lines), demonstrating scalability and high yield. Reproduced with permission from Ref. [276]. **h** Schematic representation of a dendritic tree with distributed equivalent dendrites acting as computational elements. Excitatory (green) and inhibitory (red) spike trains are received, filtered, and integrated nonlinearly (via a combination of linear summation and sigmoidal nonlinearity). The active and passive properties of dendrites allow them to perform logic operations such as AND, OR, and AND-NOT. **i** Schematic depiction of the working mechanism of artificial dendrites under electrical stimuli combined with optical modulation. **j** Volatile resistive switching curves for the on-state current clamped by the MoS_2_ channel (50 cycles). **k** Measured current response of the artificial dendrite modulated by external light pulses. **l** Logic operation toggling between AND and OR achieved by varying light intensity, with higher light intensity enabling a larger photogating current to surpass the threshold. **m** Introduction of inhibitory input to implement the AND-NOT logic operation. Reproduced with permission from Ref. [235]

High stability and endurance in neuromorphic systems can only be achieved by addressing critical challenges, including material degradation and scalability. 2D materials such as graphene, MoS_2_, and WS_2_ have shown remarkable promise for neuromorphic applications owing to their intrinsic advantages. These include high mechanical flexibility, environmental stability, and excellent switching endurance. Such characteristics make 2D material-based synaptic devices particularly suitable for long-term, reliable operation in edge computing, flexible electronics, and wearable neuromorphic systems [131, 274]. In this regard, non-volatile resistive switching in MoS_2_/graphene devices was reported to exhibit gradual potentiation and depression behaviour with near-linear weight updates under identical voltage pulses, as shown in Fig. 16a [275]. These devices demonstrated exceptional retention characteristics of up to 10^4^ s at a low operating current of 1 nA, indicating their suitability for low-power neuromorphic systems. Additionally, pulsed I–V measurements revealed synaptic behaviour with post-synaptic currents enabling approximately 100 distinct conductance states during potentiation (5 V pulses) and depression (− 4 V pulses), as depicted in Fig. 16b. The conductance, plotted against pulse numbers in Fig. 16c, highlighted a near-linear weight update with a nonlinearity factor (NLF) of 0.276 during potentiation, revealing the potential of MoS_2_/graphene devices for reconfigurable and unsupervised learning neuromorphic applications, despite observed instability in lower conductance states. Considering the stability and endurance challenges in existing neuromorphic devices, robust memristors based on a van der Waals heterostructure of graphene/MoS_2-x_O_x_/graphene (GMG) were reported, as depicted in the schematic layout of Fig. 16d [119]. The GMG devices exhibited a remarkable endurance of up to 10^7^ switching cycles and stable operation at a record-high temperature of 340 °C, demonstrating exceptional performance. During testing, over 2 × 10^7^ switching cycles were achieved using fixed voltage pulses with a width of 1 μs (+ 3.5 V for set and − 4.8 V for reset), ensuring robust and repeatable performance (Fig. 16e). Additionally, switching speed tests with fixed voltage amplitudes (+ 3 V for set and −4 V for reset) and progressively broader pulse widths confirmed the stable and reliable operation of the GMG devices, enabling their potential for high-density memory and neuromorphic computing applications in harsh environments. Subsequently, high-density memristive crossbar arrays using h-BN were fabricated on 2-inch SiO_2_/Si wafers, with h-BN serving as the resistive switching material, as shown in Fig. 16f [276]. To understand the variability and endurance properties, out of 104 memristive devices measured across crossbar arrays on a 2-inch wafer, 102 exhibited bipolar resistive switching characteristics, as shown in Fig. 16g, resulting in a high yield of approximately 98%. These results highlight the consistency and scalability of h-BN-based devices. Building on this, the potential of this technology for neuromorphic computing was explored by modelling a multilayer perceptron network using crossbar circuits integrated with Au/h-BN/Au memristors. The modelled system demonstrated a high recognition accuracy, underscoring the suitability of h-BN-based memristors for advanced neuromorphic applications. Reconfigurable neuromorphic computing further advances energy-efficient neural network implementation and functional versatility. A fully 2D-material-based heterostructure capable of performing multiple neuromorphic operations was reported, reconfiguring output terminals in response to stimuli to emulate synaptic, neuronal, and dendritic functions [235]. A hierarchical network of simplified dendritic units generated dendritic spikes, enhancing synaptic input and enabling logic operations (AND, OR, AND-NOT), supporting complex and efficient computations (Fig. 16h). An optoelectronic transistor was introduced to mimic fundamental dendritic computation by integrating excitatory and inhibitory inputs modulated by optical signals (Fig. 16i). DC current–voltage sweeps over 50 cycles (Fig. 16j) exhibited volatile resistive switching characteristics. The proposed artificial dendrite, featuring a Gr electrode for excitatory input and a back gate for inhibitory input, enabled both temporal and spatial integrations. Optical signal filtering modulated the current response under successive voltage pulses (Fig. 16k), while Fig. 16l, m demonstrated its temporal and spatial integration capabilities, respectively, emphasizing its potential for advanced neuromorphic computations. These advancements in 2D-material-based neuromorphic devices, combining stability, endurance, and reconfigurability, demonstrate their transformative potential for scalable, energy-efficient, and versatile neural network applications. From a benchmarking perspective, MoS_2_ and WSe_2_ provide one of the best overall balances between optical response, switching tunability, and material maturity. h-BN is particularly suitable for dielectric integration and low-power operation, while BP offers advantages in anisotropic transport and optical sensitivity. Emerging materials such as tellurene and Xenes provide high mobility and multifunctionality, although their fabrication maturity remains limited. Organic 2D materials are promising for flexibility and biocompatibility, but their endurance and long-term stability still require improvement. In this regard, Table 3 compares a wide range of neuromorphic device architectures based on 2D materials integrated with machine-learning models across different paradigms, including supervised, unsupervised, spiking, and adaptive learning. These devices demonstrate functional capabilities such as STDP, PPF, multilevel conductance, and optical encoding, with performance metrics including recognition accuracy (up to 98.55%), low switching energy (down to femtojoule levels), high endurance (up to 10^7^ cycles), and robustness under mechanical stress. This comparative summary highlights the diverse mechanisms through which 2D materials support neuromorphic behaviour while also emphasizing their practical applicability in edge computing, real-time learning, and intelligent sensing platforms.

**Table 3:** Comparison of neuromorphic devices based on 2D materials, detailing their architectures, learning paradigms, functional capabilities (e.g. STDP, PPF, optical encoding), and key performance metrics such as accuracy, energy efficiency, and endurance

Device type	Material/architecture	Learning algorithm	Key features	Key performance(s)	Performance metrics	References
Memristor	MoS_2_	Supervised learning	High linearity in synaptic weight updates; integrated into ANN for MNIST dataset classification	Linearity: high; on/off ratio: ~ 10^2^; switching voltage: ~ 0.1–1.5 V	Recognition accuracy: 98.55% (HW-NN); matches 99.41% of SW-NN	[244]
Memristor	2D Ti_3_C_2_T_x_ MXene	Supervised learning	High-speed resistance switching; Layer-by-layer self-assembled MXene multilayers	Operating energy: ~ 80 fJ µm^−2^ endurance: > 10^8^ cycles;	Learning accuracy: 90–93% (MNIST); endurance: > 10 S cycles; energy: ~ 80 fJ µm^−2^	[277]
Artificial synaptic transistor	Graphene-based memristor	Supervised learning	Metaplasticity; biocompatibility; low switching energy; accurate gradient descent for neural network training	Switching energy; ~ 50 aJ µm^−2^ switching voltage: < 2.5 V	Recognition accuracy: 96.8% (Fashion-MNIST); power consumption: < 1 nW	[259]
FETs synapse	MoS_2_	Supervised learning	Paired-pulse facilitation (PPF); STM to LTM transition under gate voltage and light modulation	switching voltage: < 5 V; retention: > 10^3^ s	Recognition accuracy: 85.5% (MNIST); energy density: < 1 pJ	[270]
Memristor	h-BN	Unsupervised learning	Multi-level conductance; effective for Hebbian learning and unsupervised clustering	Longer Retention: > 10^4^ cycles; on/off ratio: ~ 10^3^–10^4^; switching voltage: ~ 0.5–2 V	Accuracy: ~ 80% (MNIST clustering task); endurance: > 10^5^ cycles	[139]
Artificial synapse	Graphene-based memristive device	Unsupervised learning	Efficient STDP; robust performance in spiking neural networks	on/off ratio: ~ 10^2^; Switching voltage; < 4 V	Accuracy: ~ 85% (MNIST classification); retention: > 10^5^ s	[255]
Optical neuromorphic device	MoS_2_	Spiking neural network	Multilayer MoS_2_ device for neuromorphic applications; demonstrates optical potentiation and depression	Retention: 1.4 × 10^3^ s; switching voltage: ~ 1–3 V; on/off ratio: ~ 10^2^	Retention time: 1.4 × 10^3^ s; accuracy: 41% (MNIST dataset)	[278]
Memristive Crossbar Array	Au/h-BN/Au	Stochastic regression	High-yield fabrication; improves regression model convergence	Endurance: > 10^5^ cycles; yield: 98%; switching voltage: < 2 V	Yield: 98% (across 2-inch wafers); endurance: > 10^7^ cycles	[276]
Reconfigurable neuromorphic device	Graphene/MoS_2_/Graphene Heterostructure	Adaptive learning	Performs logic operations (AND, OR, AND-NOT); supports spatiotemporal computations	On/off ratio: > 10^4^	Stability: > 50 cycles; logic accuracy: ~ 100%	[131]
Photodiode array	WSe_2_	Responsivity evaluation	Tunable photoresponsivity for synaptic weight modulation; direct optical encoding	Responsivity: −60 to + 60 mA W^−1^; switching voltage: ~ 1–5 V	Responsivity range: −60 to + 60 mA W^−1^; accuracy: ~ 100%	[269]
Neuromorphic accelerator	Graphene/BN	Edge AI and wearables	Ultra-flexible design; stable performance under mechanical stress	Operational stability: > 400 cycles; bending radius: ~ 5 mm	Recognition accuracy: 94.5% (MNIST); operational stability: > 400 cycles	[273]

## Applications and Future Directions

**Fig. 17:**
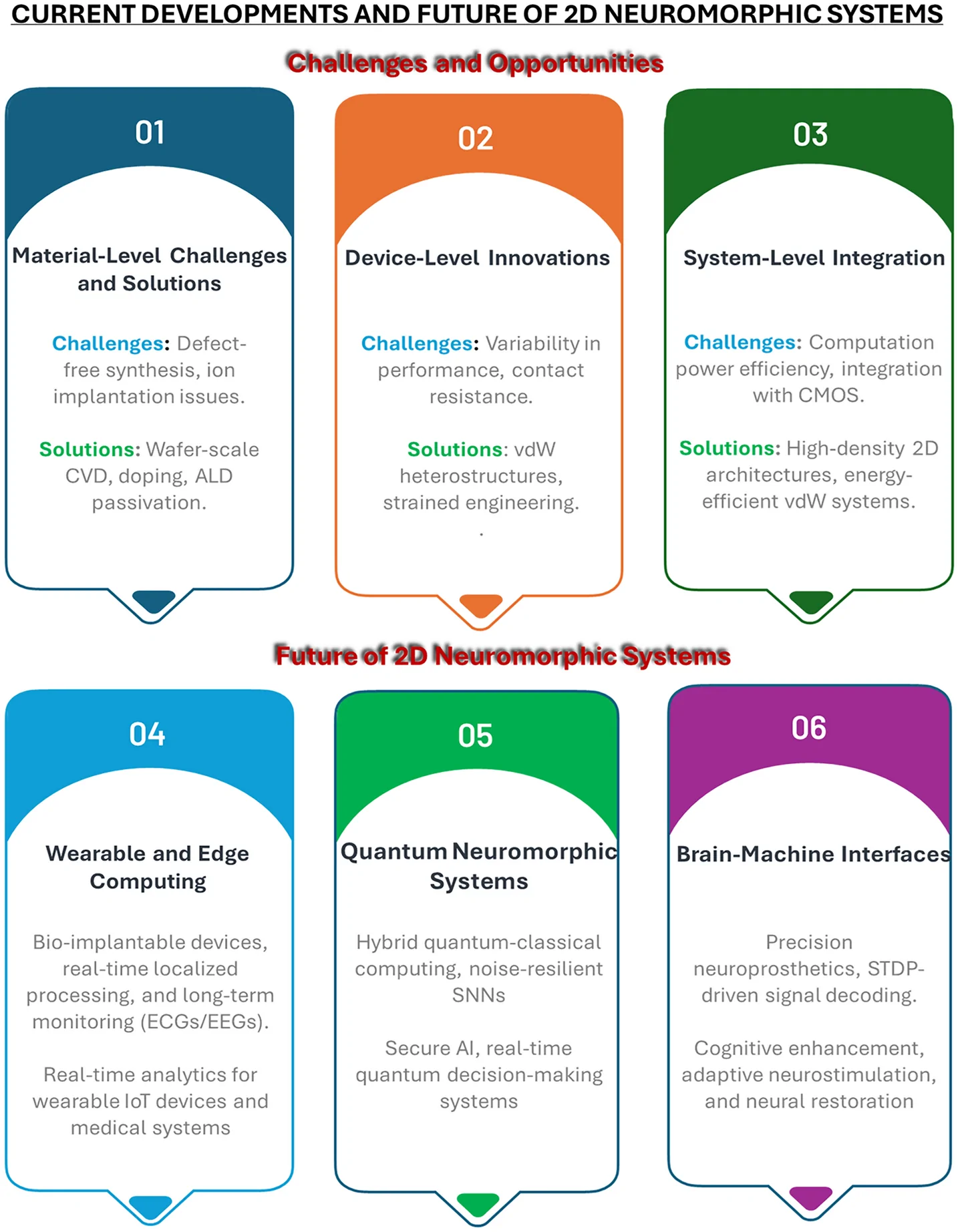
Current challenges and future directions of 2D materials-enabled neuromorphic computing system

This section explores the emerging applications and research directions that will shape the future of neuromorphic systems. The overview of this section is highlighted in Fig. 17.

### Wearable and Edge Computing: Integration of Flexible 2D Neuromorphic Devices in Bio-Implantable and Portable Electronics

The integration of flexible 2D materials like TMDs, h-BN, and BP into wearable and edge devices has unlocked unprecedented potential for use in low-power, adaptive electronics. Exploiting their high mechanical flexibility, ultrathin dimensions, and exceptional electrical properties, these materials enable neuromorphic functionalities directly within bio-implantable and portable platforms. Recent studies showed that memristive devices based on MoS_2_ and WSe_2_ can emulate LTP and STP, allowing for localized, energy-efficient processing of physiological signals such as ECGs and EEGs. These neuromorphic devices, when embedded in bio-implantable systems, provide continuous, real-time monitoring while consuming femtojoules per operation, significantly extending operational lifespans in resource-constrained environments.

In edge computing, where real-time data processing is critical, 2D materials offer unique advantages through their ability to integrate seamlessly with CMOS architectures. Systems incorporating optoelectronic synapses based on 2D materials, such as TMDs and heterostructures, demonstrate superior temporal response and signal processing efficiency, outperforming traditional hardware in latency-sensitive applications. For instance, 2D photonic synapses enable rapid optical signal transduction, offering significant improvements in edge analytics for wearable medical devices and IoT systems. Such advancements reduce reliance on cloud-based computing, improving data privacy and reducing energy demands for remote healthcare monitoring. Recently, Lee et al. [279] report the integration of nanoscale MoS_2_ memristors (with an active area of ~ 0.015 µm^2^) in the back-end-of-line (BEOL) of 350 nm CMOS microchips. The devices show forming-free operation with low SET (~ 0.23 V) and RESET (~ − 0.1 V) voltages, as well as low cycle-to-cycle variability across 19 devices. The study demonstrates the potential for MoS_2_-based memristors to be incorporated into CMOS microchips for monolithic integration and processing-in-memory systems, which aligns with our translational outlook on neuromorphic hardware.

Despite these breakthroughs, challenges remain in achieving large-scale integration and long-term stability of 2D materials under ambient conditions. Recent advances in scalable synthesis methods, such as CVD and ALD, are addressing these limitations, ensuring consistent quality and reproducibility of neuromorphic components. Further exploration of passivation techniques and defect engineering will be key to enhancing device robustness for prolonged use in bio-implantable and wearable electronics. As the field progresses, the synergistic integration of 2D neuromorphic devices into flexible, low-power platforms is expected to revolutionize healthcare, wearable technology, and adaptive computing at the edge. These applications are closely linked to the intrinsic flexibility, low operating power, and biocompatibility of 2D materials, which make them particularly attractive for portable and bio-integrated neuromorphic systems.

### Brain–Machine Interfaces: The Potential of Emerging Materials in Developing Interfaces that Closely Mimic Biological Neurons

BMIs have witnessed transformative potential with the advent of 2D materials like TMDs, h-BN, and BP, offering an unparalleled ability to replicate synaptic plasticity and neural dynamics. Devices such as memristors and memtransistors based on MoS_2_, WSe_2_, and heterostructures enable fine-tuned STDP, facilitating bidirectional signal transduction between neural networks and electronic systems. These 2D devices, when incorporated into BMIs, exhibit high spatial resolution, ultra-low power requirements, and biocompatibility, paving the way for advanced neuroprosthetics capable of restoring motor and sensory functions with precision.

The optical and electrical tunability of 2D neuromorphic devices further enables high-performance neural signal decoding and processing within BMIs. Optoelectronic synapses based on WS_2_ and heterobilayers demonstrate exceptional signal-to-noise ratios in decoding spike trains, which are essential for seamless brain-to-machine communication. By exploiting localized in-memory computing, these systems reduce the dependence on external processing, offering real-time response critical for applications such as adaptive neurostimulation and closed-loop cognitive enhancement systems. Recent developments integrating these devices into soft, flexible substrates have enabled minimally invasive implants that mimic the brain’s elasticity and adaptability.

Despite significant progress, scaling these interfaces for robust, long-term performance remains a challenge. Emerging fabrication techniques, including wafer-scale growth and defect passivation strategies, have shown promise in ensuring material consistency and device stability under physiological conditions. Advanced encapsulation methods are being explored to mitigate degradation in biofluids, further supporting their long-term deployment in BMIs. As research continues to push the boundaries, the convergence of 2D neuromorphic devices with BMIs is poised to redefine neurotechnology, offering unprecedented possibilities in neuroprosthetics, cognitive enhancement, and the treatment of neurological disorders. The ability of 2D neuromorphic devices to emulate synaptic plasticity, multilevel conductance, and low-power signal processing makes them highly suitable for next-generation BMIs.

### Quantum Neuromorphic Systems: Exploring the Role of Quantum Properties in Future 2D-material-based Neuromorphic Architectures

Quantum neuromorphic systems aim to utilize quantum effects, such as tunnelling, coherence, and spin-related phenomena, to enhance the capabilities of neuromorphic architectures beyond classical limits. 2D materials, with their atomic precision and tuneable electronic properties, are uniquely positioned to bridge the gap between quantum mechanics and neuromorphic computing. For instance, TMDs and vdW heterostructures exhibit quantum tunnelling and interlayer coupling, enabling non-volatile switching with minimal energy dissipation. These properties are particularly relevant for quantum synaptic devices capable of multilevel conductance, a requirement for achieving complex neural behaviours like STDP and PPF.

Hybrid quantum–classical neuromorphic systems have been explored by integrating 2D materials with quantum computing platforms. For example, quantum dots in WSe_2_ or MoSe_2_ offer single-photon emission capabilities that enable spike-based quantum signal processing. Similarly, spintronic properties in materials like CrI_3_ provide opportunities for spin-based neuromorphic networks with ultrafast switching dynamics. Besides enhancing the efficiency of spiking networks, these systems also introduce quantum parallelism, allowing for high-dimensional computations that classical systems struggle to handle. This hybrid approach may provide a basis for quantum SNNs capable of solving optimization problems with improved robustness to noise.

The integration of quantum properties into neuromorphic architectures presents significant material and fabrication challenges. Achieving uniformity in TMDs and heterostructures while preserving coherence requires precise control over synthesis techniques like CVD and ALD. Moreover, maintaining device stability under operational conditions necessitates advanced encapsulation strategies, such as h-BN passivation, to mitigate environmental decoherence. Despite these challenges, recent advances suggest that scalable quantum neuromorphic systems may find applications in real-time quantum AI, secure encryption, and decision-making tasks that leverage the unique properties of quantum systems. These quantum-inspired approaches further expand the role of 2D materials beyond conventional synaptic devices, highlighting their potential for future high-dimensional and energy-efficient computing.

### Challenges and Opportunities: Addressing Scalability, Reproducibility, and the Material Synthesis Challenges Ahead

A primary barrier remains the scalable synthesis of high-quality 2D materials with uniform thickness, defect-free surfaces, and reproducible electronic properties. Currently used techniques such as CVD and MBE have enabled substantial progress, yet maintaining inter-batch consistency, large-area uniformity, and cost-effectiveness remains a significant hurdle. Innovations in wafer-scale growth, advanced doping techniques, and defect passivation are critical to overcoming these bottlenecks and enabling commercial-scale production of neuromorphic devices [280]. Most 2D-material-based neuromorphic devices are still at an early stage of development, where demonstrations are largely limited to single devices, proof-of-concept memristors, memtransistors, photonic synapses, and small crossbar arrays. Progress toward practical applications will require wafer-scale synthesis, reproducible device characteristics, BEOL-compatible fabrication, and reliable CMOS integration. In addition, these physical vapour deposition methods are energy intensive, therefore causing substantial carbon emissions during the growth of 2D materials. Marraccini et al. [281] demonstrate the scalable, cost-effective fabrication of Ag/MoS_2_/Au memristors using a roll-to-roll mechanical exfoliation process coupled with inkjet printing. The resulting devices exhibit a resistance ratio between 10^2^ and 10^4^, with robust retention times exceeding 10^3^ s. Additionally, the authors demonstrate a virtual memristor crossbar array for neural network classification, showing high accuracy with both 3-bit (100%) and 4-bit (94%) quantized weights on MNIST. This paper supports our scalability/manufacturing discussion and links memristor fabrication directly to neural network applications.

Reproducibility across devices poses another significant challenge. Variability in material properties, particularly in TMDs and heterostructures, often causes inconsistencies in device performance. For example, non-uniformities in switching thresholds, conductance states, or interlayer coupling in vdW heterostructures can hinder large-scale integration. In addition to material-level variability, large-scale neuromorphic arrays also face important system-level challenges. Sneak current paths in dense crossbar architectures can distort read and write operations, making selector devices essential for suppressing leakage currents and improving array reliability. Peripheral circuitry, including ADCs, DACs, amplifiers, and control units, can also contribute substantial area and energy overhead, reducing the overall efficiency advantage of neuromorphic systems. Furthermore, IR drop across large interconnect networks may lead to non-uniform voltage distribution, while thermal accumulation during repeated switching can degrade device stability and endurance. Addressing this issue requires developing robust fabrication protocols that ensure statistical uniformity in device behaviour, supported by real-time process monitoring techniques and high-throughput characterization methods. Therefore, achieving large-scale neuromorphic integration requires not only improvements in material synthesis and reproducibility, but also careful co-optimization of device architecture, selector integration, peripheral circuitry, and thermal management. Furthermore, exploiting AI-driven material informatics can accelerate the discovery of 2D materials with tailored properties, optimizing device performance for specific neuromorphic applications.

Despite these challenges, the opportunities for 2D neuromorphic systems are transformative. The unique combination of quantum effects, optoelectronic tunability, and energy efficiency inherent to 2D materials positions them as pivotal enablers for next-generation computing. However, not all device platforms are at the same level of maturity. Single-device demonstrations, emerging materials such as tellurene, silicene, and Xenes, as well as proof-of-concept photonic and spintronic synapses, are still in the early stages of development. Small-scale crossbar arrays, wafer-grown h-BN and MoS_2_ memristors, and hybrid experimental-computational systems are relatively more advanced. In contrast, hybrid CMOS/memristor architectures and commercial neuromorphic processors such as Intel Loihi and IBM TrueNorth are much closer to large-scale implementation. In particular, hybrid CMOS/2D-material systems are likely to provide the most realistic near-term route toward commercialization, as fully 2D-material-only architectures still face major challenges in scalability, reproducibility, and large-area integration. By achieving breakthroughs in scalability and reproducibility, these systems can redefine computing paradigms, paving the way for sustainable, adaptive, and intelligent technologies. In addition, future progress in 2D-material-based neuromorphic systems will depend not only on discovering new materials, but also on improving material uniformity, defect control, wafer-scale synthesis, long-term reliability, and CMOS-compatible integration. In particular, balancing switching performance, scalability, and energy efficiency will be essential for translating laboratory-scale demonstrations into practical neuromorphic hardware.

## Conclusions and Perspectives

The integration of 2D materials into neuromorphic computing architectures marks a significant advancement in developing next-generation information technologies. By exploiting their unique atomic-scale thickness and exceptional electronic, optoelectronic, and quantum mechanical properties, materials such as TMDs, h-BN, BP, and emerging tellurene-based materials could potentially address the fundamental limitations of conventional von Neumann computing paradigms. These systems facilitate the realization of devices capable of closely mimicking neuronal and synaptic behaviour, demonstrating substantial promise for low-power, high-performance, real-time adaptive systems.

One of the most remarkable strengths of 2D materials lies in their inherent versatility and tuneable properties. Specifically, TMD-based devices have demonstrated significant synaptic plasticity through mechanisms such as chalcogen vacancy migration and controlled phase transitions, enabling both volatile and non-volatile memory functionalities crucial for neuromorphic systems. Furthermore, h-BN’s role as an insulating and dielectric layer in multilayered device structures has enhanced synaptic response speed and energy efficiency, crucial for implementing scalable neuromorphic circuits. BP’s anisotropic electronic and optoelectronic properties enable sophisticated functionalities such as optical synapses, further extending the application spectrum of neuromorphic computing toward advanced vision systems and dynamic pattern recognition.

Nevertheless, critical challenges persist that must be resolved before these laboratory achievements translate into viable products. Foremost among these is the reproducible large-scale synthesis of high-quality 2D materials. Although CVD and ALD processes have made considerable strides toward scalability and uniformity, further improvements in defect engineering and precise doping methods are necessary to consistently achieve atomic-scale uniformity and material quality. Ensuring defect-free interfaces, reliable doping techniques, and stable grain boundaries will be pivotal in moving from isolated device demonstrations to integrated large-scale arrays.

Additionally, the device-to-device uniformity of 2D-material-based neuromorphic devices under operational conditions remain major concern. Device longevity and robustness in varying environmental and operational contexts—such as fluctuations in temperature, mechanical stress, and ambient exposure—require continued focus on materials engineering and protective encapsulation strategies. Furthermore, quantum mechanical phenomena intrinsic to many 2D materials add layers of complexity to device fabrication and integration. To effectively harness quantum effects such as tunnelling and spin-dependent phenomena, rigorous control over synthesis conditions and advanced fabrication techniques must be developed to maintain coherence, reduce noise, and minimize decoherence, critical to quantum neuromorphic functionalities.

Furthermore, CMOS-compatible integration remains major barriers for practical deployment of 2D-material-based neuromorphic systems. Device-to-device variation can lead to non-uniform switching thresholds, conductance states, and synaptic responses across large arrays, while cycle-to-cycle fluctuations may reduce the precision and repeatability of learning operations. In addition, long-term conductance drift, retention instability, and stochastic switching behaviour can degrade the accuracy of stored synaptic weights over time. Environmental degradation caused by oxygen, moisture, temperature variation, and interfacial defects can further reduce device stability, particularly for air-sensitive materials such as BP and some vdW heterostructures. Therefore, future progress will require improved encapsulation, defect engineering, process control, and adaptive compensation algorithms to ensure reliable large-scale neuromorphic operation.

The future of neuromorphic computing anchored by 2D materials looks quite promising, particularly through integration with advanced machine-learning algorithms, quantum computational methodologies, and flexible electronic technologies. The convergence of these fields promises breakthroughs in edge artificial intelligence, autonomous robotics, brain–machine interfaces, personalized healthcare diagnostics, and environmental sensing. In particular, neuromorphic systems exploiting 2D materials could enable powerful localized computation with unprecedented energy efficiency, directly addressing urgent global demands for sustainable computing solutions.

To capitalize on these promising opportunities, multidisciplinary collaboration will be indispensable. Bridging expertise across materials science, physics, computational modelling, electronics, neurobiology, and artificial intelligence will facilitate comprehensive solutions addressing both fundamental material challenges and complex system-level integration tasks. Furthermore, an emphasis on algorithmic development specifically tailored for neuromorphic architectures could optimize the computational capabilities of these systems, ensuring seamless compatibility with existing computational infrastructures and facilitating widespread adoption.

Ultimately, addressing these challenges through targeted research and collaborative innovation will allow 2D materials to establish a new computing paradigm, inspired by the extraordinary efficiency and adaptability of biological systems. Such a paradigm shift could meet contemporary computational challenges and simultaneously redefine the capabilities of future technologies, significantly impacting multiple sectors of society. The continuous evolution and deeper integration of 2D materials into practical neuromorphic systems thus present a compelling frontier, poised to fundamentally reshape our technological landscape.
